# Mannose-Presenting “Glyco-Colicins” Convert the Bacterial Cell Surface into a Multivalent Adsorption Site for Adherent Bacteria

**DOI:** 10.1021/jacsau.4c00365

**Published:** 2024-06-12

**Authors:** Natasha E. Hatton, Joe Nabarro, Nicholas D. J. Yates, Alison Parkin, Laurence G. Wilson, Christoph G. Baumann, Martin A. Fascione

**Affiliations:** † Department of Chemistry, University of York, York, YO10 5DD, United Kingdom; ‡ Department of Physics, University of York, York, YO10 5DD, United Kingdom; § Department of Biology, University of York, York, YO10 5DD, United Kingdom

**Keywords:** neoglycoproteins, bioconjugation, mannose, colicins, FimH bacterial aggregation

## Abstract

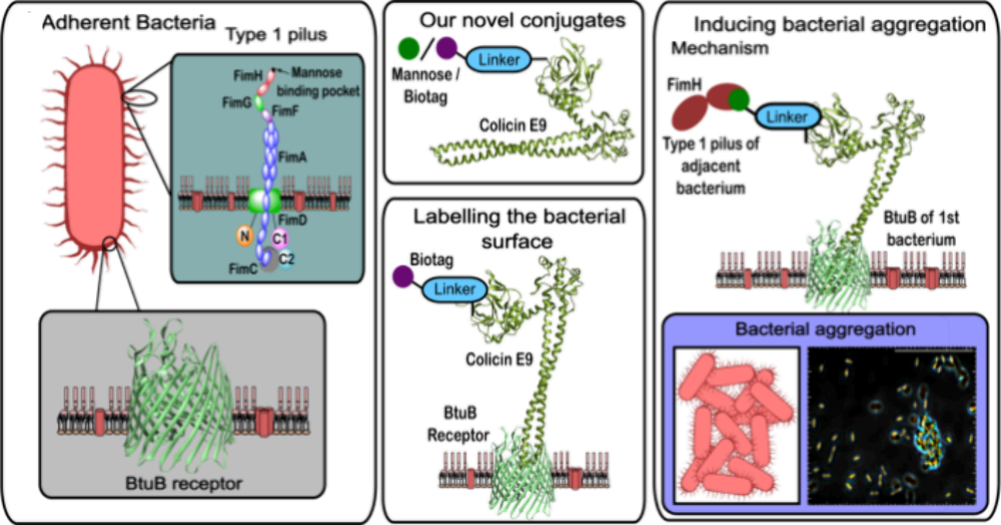

Biofilm formation is integral to the pathogenesis of numerous adherent bacteria and contributes to antimicrobial resistance (AMR). The rising threat of AMR means the need to develop novel nonbactericidal antiadhesion approaches against such bacteria is more urgent than ever. Both adherent-invasive *Escherichia coli* (AIEC, implicated in inflammatory bowel disease) and uropathogenic *E. coli* (UPEC, responsible for ∼80% of urinary tract infections) adhere to terminal mannose sugars on epithelial glycoproteins through the FimH adhesin on their type 1 pilus. Although mannose-based inhibitors have previously been explored to inhibit binding of adherent bacteria to epithelial cells, this approach has been limited by monovalent carbohydrate–protein interactions. Herein, we pioneer a novel approach to this problem through the preparation of colicin E9 bioconjugates that bind to the abundant BtuB receptor in the outer membrane of bacteria, which enables multivalent presentation of functional motifs on the cell surface. We show these bioconjugates label the surface of live *E. coli* and furthermore demonstrate that mannose-presenting “glyco-colicins” induce *E. coli* aggregation, thereby using the bacteria, itself, as a multivalent platform for mannose display, which triggers binding to adjacent FimH-presenting bacteria.

Many pathogenic bacteria, including *Escherichia coli*, *Pseudomonas aeruginosa*, and *Staphylococcus* aureus, have the ability to form biofilms,^1^ which can provide increased protection from antibiotics^2^ and the host immune system^3^ and are thus implicated in pathogenesis.^4^ Adhesion is one of the first steps in biofilm formation^5^ and is mediated by adhesins located on pili or fimbriae on the surface of bacteria. These hairlike appendages are able to recognize and bind to specific receptor moieties on the host cell.^6^ FimH, a two-domain protein located at the terminus of the type 1 pili,^7^ is an adhesin with a mannose-binding pocket located on the end of its lectin domain (Figure 1a).^8,9^ The FimH gene is detectable in 90% of *E. coli* strains,^10^ and FimH is used by both adherent-invasive *E. coli* (AIEC)^11^ and uropathogenic *E. coli* (UPEC)^12^ to adhere to host cells via terminal mannose units on epithelial glycoproteins.^7^ These *E. coli* strains are well-known pathogens; UPEC is responsible for over 80% of uncomplicated urinary tract infections (UTIs)^13^ with an estimated yearly health care cost greater than $1 billion,^14^ and AIEC has been implicated in inflammatory bowel disease in Crohn’s disease patients.^11^ Antibiotics remain the frontline treatment for these infections;^15^ however, increasing antibiotic resistance rates coupled with international guidelines advising against the use of antibiotics in Crohn’s sufferers^16^ means there is an urgent need to develop nonbactericidal treatments against these adhesive bacteria. This has led to a wealth of elegant research into the use of monomeric α-d-mannopyranoside glycans as antiadhesion therapies that bind to FimH.^17,18^ However, a fundamental limitation of many of these molecules is that they only participate in monovalent binding, whereas in nature, carbohydrate ligand presentation is predominately multivalent,^21−25^ and although impressively constructed mannose-based dendrimers typically have higher potencies than their monovalent counterparts,^23,26−28^ they can also be more challenging to access.^29,30^

**Figure 1:**
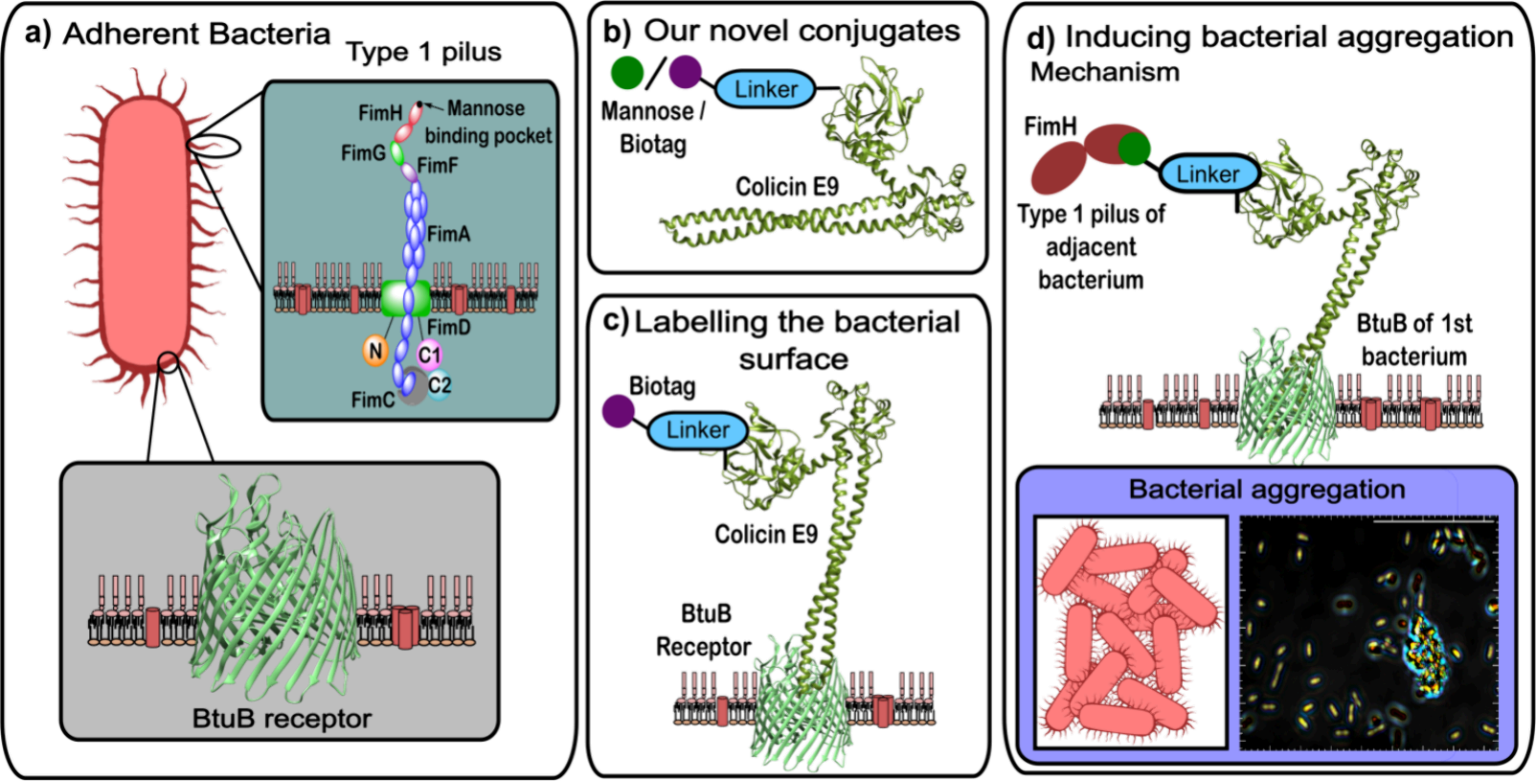
A depiction of (a) adherent *E. coli* highlighting both the type 1 pilus and the BtuB (PDB: 3RGM^19^) receptor on the surface of the bacteria and (b) the design of our novel colicin conjugates where a biologically relevant motif is attached by a linker to colicin E9 (PDB: 5EW5^20^). The application of these conjugates for (c) labeling of the bacterial surface with biologically relevant motifs and (d) the initiation of bacterial autoaggregation when using a mannose-linked conjugate.

We hypothesized that colicin proteins labeled with mannose sugars could form the basis of novel nonbactericidal agents against adhesive bacteria. Colicins are small proteins produced by some *E. coli* strains, which are toxic to others,^31^ and bind to their target receptors with high (i.e., nanomolar) affinity.^32^ They are promising agents for use in therapeutic contexts as their narrow targeting range could potentially facilitate single-strain targeting,^33^ while the lack of their target receptor on mammalian cells means off-target interactions are unlikely.^34^ Notably, while native colicins are used as offensive weapons against competing *E. coli* strains,^35^ Penfold et al. demonstrated that the installation of a “top lock” in the R-domain of colicin E9 inhibits toxicity while not adversely affecting the binding of colicin E9 to its target receptor (the BtuB outer membrane protein receptor),^32^ thereby allowing colicin E9 to serve as a nonbactericidal agent for labeling *E. coli*.^35,36^ Herein, we present the design and synthesis of colicin conjugates presenting functional motifs, such as (i) biotin, (ii) fluorescein, and (iii) α-d-mannopyranosides. We demonstrate that the conjugation of functional motifs to colicin E9 can be achieved by an organocatalyst-mediated protein aldol ligation (OPAL) (Figure 1b) and that appending functional motifs to colicin E9 does not prevent the binding of the protein to its target BtuB receptor (Figure 1c). Furthermore, we demonstrate the premise of using α-d-mannose-presenting colicin E9 neoglycoproteins as heterobiselective binding agents that can induce aggregation in samples of *E. coli* K12 substr. BW25113 via the simultaneous binding of FimH and BtuB motifs. We propose that the initial high-affinity binding of colicin units to constitutively expressed (∼500 per cell) BtuB receptors (Figure 1d) results in the labeling of *E. coli* surfaces with multiple α-d-mannose motifs, which are thereafter capable of multivalent binding to FimH adhesins on neighboring bacterial surfaces.

Use of colicin E9 to label *E. coli* with biologically relevant motifs necessitates the conjugation of the colicin to the desired motifs using a site-specific bioconjugation approach. The N-terminal T domain of colicin E9 has been shown to be a structurally disordered region with a high degree of flexibility and distal to the colicin E9 binding site.^32,37^ We therefore hypothesized that modification at this site would not preclude binding of colicin E9 to its receptor. Conveniently, the N-terminal residue of colicin E9 is a serine, and thus, the installation of a bio-orthogonal α-oxo aldehyde motif at this site was easily achieved through a mild treatment with NaIO_4_ (Figure 2c). This α-oxo aldehyde could subsequently be targeted through OPAL,^38^ a site-specific bioconjugation method previously developed in our lab that uses proline (tetrazole)-based secondary amines as organocatalysts to mediate cross-aldol ligation between an enolizable aldehyde-functionalized probe bearing a biologically relevant motif and a nonenolizable electrophilic α-oxo aldehyde, in this example enabling the desired chemical motif to be appended to the N-terminus of colicin E9 (Figure 2c). To demonstrate that OPAL conjugation to the N-terminus of colicin E9 was possible, a sample of α-oxo aldehyde-functionalized colicin E9 was subjected to OPAL with a biotin-presenting OPAL probe.^39,40^ Successful ligation was validated using both SDS-PAGE gel and Western blot analysis (Figure 2a). Because of the size of colicin E9 (61 kDa), the addition of the biotinylated OPAL probe only results in a subtle observable increase in the mass of the colicin E9 band in SDS-PAGE (Figure 2a,i), yet Western blot visualization using a streptavidin horseradish peroxidase-conjugate that binds biotin noncovalently with femtomolar affinity unequivocally demonstrated that the biotin had been successfully attached (see Figure 2a,ii). To prove that conjugation via OPAL does not prevent binding of colicin E9 to the BtuB receptor, a fluorescein-labeled colicin E9 conjugate was also prepared enabling BtuB binding to be visualized via fluorescence microscopy (Figure 2b). The successful labeling of colicin E9 with fluorescein was also confirmed via SDS-PAGE gel analysis (Figure 2b,i) in which a subtle mass increase can be observed after bioconjugation of the fluorescein-labeled OPAL probe confirmed by fluorescent visualization (Figure 2b,ii).

**Figure 2:**
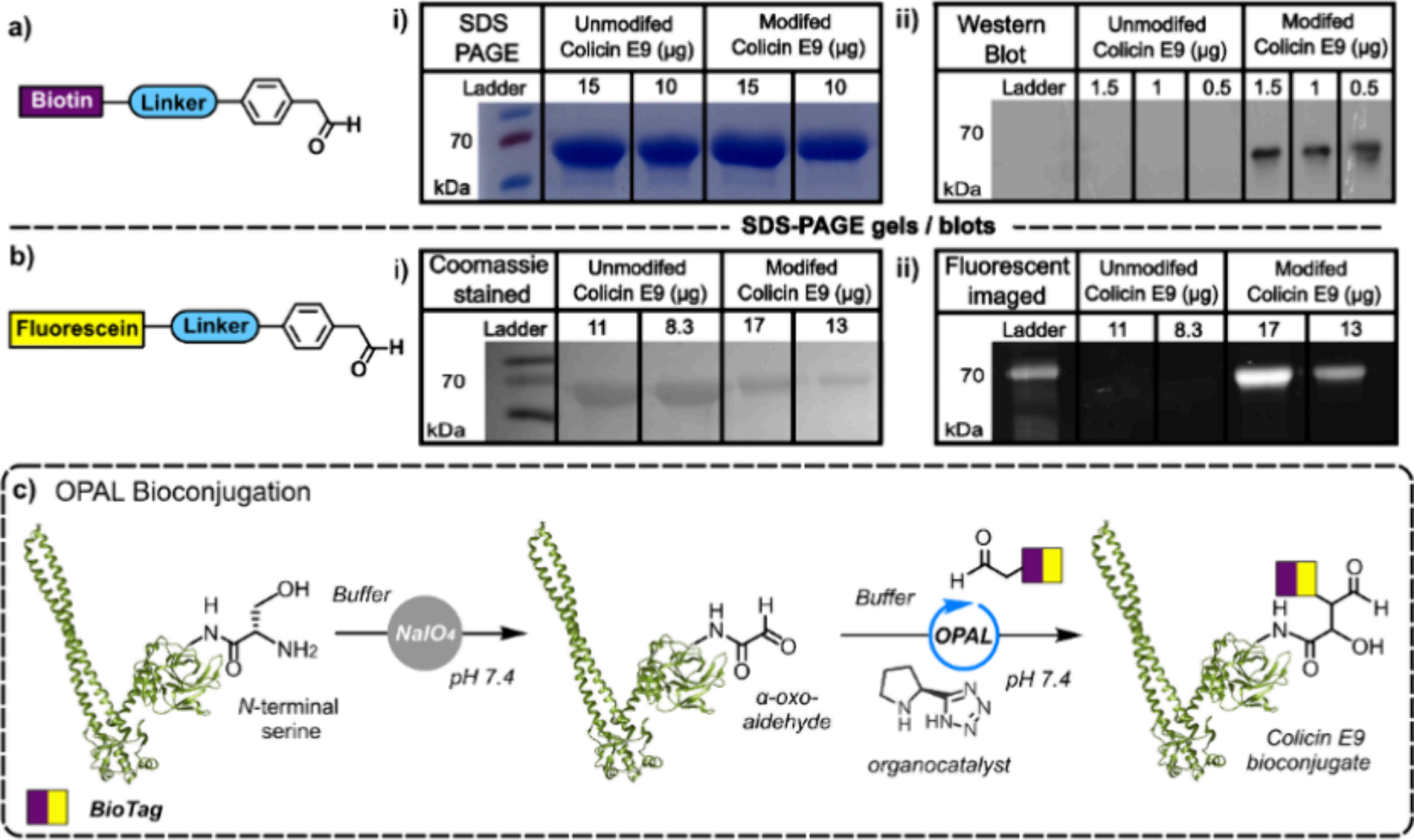
(a) A depiction of the biotin-linked OPAL probe, (i) SDS-PAGE analysis of biotin-labeled colicin E9, and (ii) Western blot analysis of biotin-labeled colicin E9. (b) A depiction of the fluorescein-linked OPAL probe and SDS-PAGE gel analysis of the fluorescein labeled colicin E9 conjugate visualized using either (i) Coomassie staining or (ii) fluorescent imaging. (c) The oxidation of the N-terminal serine residue of colicin E9 can be used to install an α-oxo aldehyde motif that can be site-selectively targeted using proline tetrazole OPAL to append a biologically relevant motif (BioTag) to the N-terminus of colicin E9.

The fluorescein-labeled conjugate was subsequently incubated with samples of wild-type *E. coli* BW25113 and BW25113 Δ*btuB*. These samples were then imaged via 3D-structured illumination microscopy (3D-SIM) and confocal fluorescence microscopy. Fluorescence signal was observed in wt cells (Figure 3a,c), while no significant fluorescence was observed in Δ*btuB* (Figure 3b). This shows specific fluorescein-labeled conjugate binding to BtuB outer membrane protein on the surface of *E. coli* cells while providing vital evidence that OPAL ligation does not preclude binding to surface BtuB receptor.

**Figure 3:**
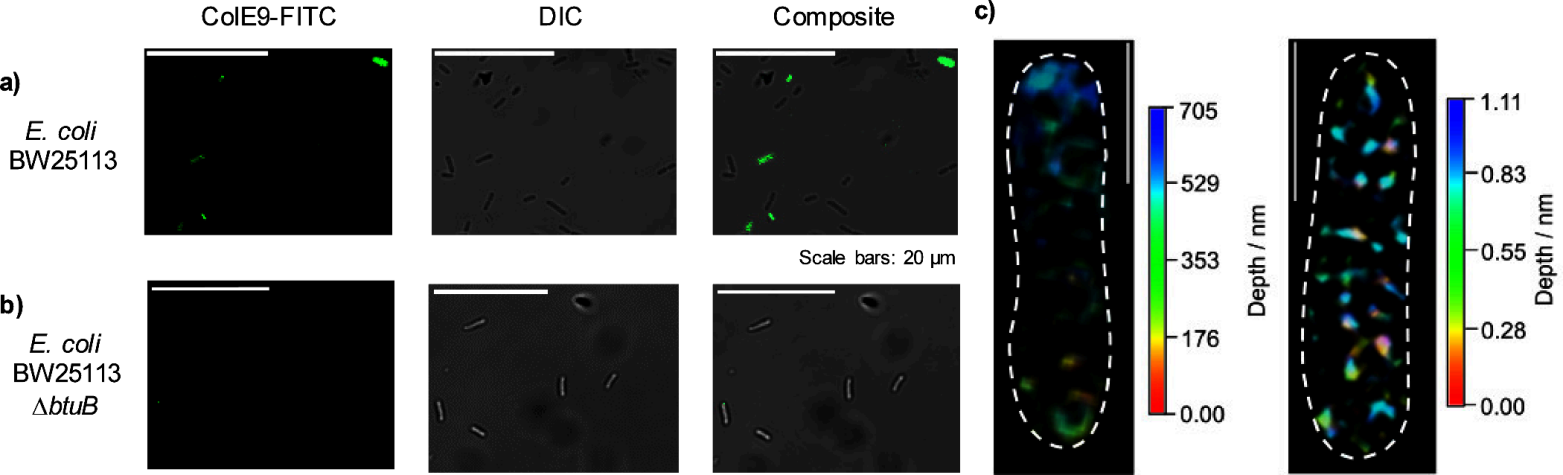
(a) Representative fluorescence and differential interference contrast (DIC) confocal microscopy images of a sample of BW25113 wt cells incubated with 500 nM fluorescein-labeled conjugate. (b) Representative fluorescence and DIC confocal microscopy images of a sample of BW25113 Δ*btuB* incubated with 500 nM fluorescein-labeled colicin E9. (c) Representative 3D-SIM images of depth-coded BW25113 cells labeled with 500 nM fluorescein-labeled colicin E9.

Following successful demonstration of colicin E9 OPAL labeling as a reliable method for appending biologically relevant molecules to the surface of *E. coli*, we hypothesized that a mannose-presenting colicin E9 conjugate could be used to label the surface of *E. coli* with multiple glycans, thereby effectively turning each bacterium into a mannose-presenting “glyco-dendrimer.” Surface-exposed mannose units may then bind FimH units on nearby *E. coli* cells in a trans-fashion, thereby resulting in cell-to-cell adhesion and potentially autoaggregation. To test this hypothesis a mannose-bearing OPAL probe first had to be synthesized. Many different mannose scaffolds have previously been investigated for use against UPEC, including biphenyl mannosides,^41−45^ (neo)thiazolylaminomannosides,^46,47^ septanose,^48^ and squarate mannosides.^19,49^ An α-mannose-presenting squarate mannoside scaffold was identified as suitable for appending to an OPAL probe as (i) mannose azide **4** could be synthesized relatively simply in four steps (Figure 4a), (ii) squarate mannosides have been shown in literature to have high affinity for FimH with analogues achieving IC_50_ values in the micromolar range,^50^ and (iii) the squarate ester scaffold allows for easy installation of bioconjugation handles through the substitution of the terminal squarate methoxy group with an appropriate amine.

**Figure 4:**
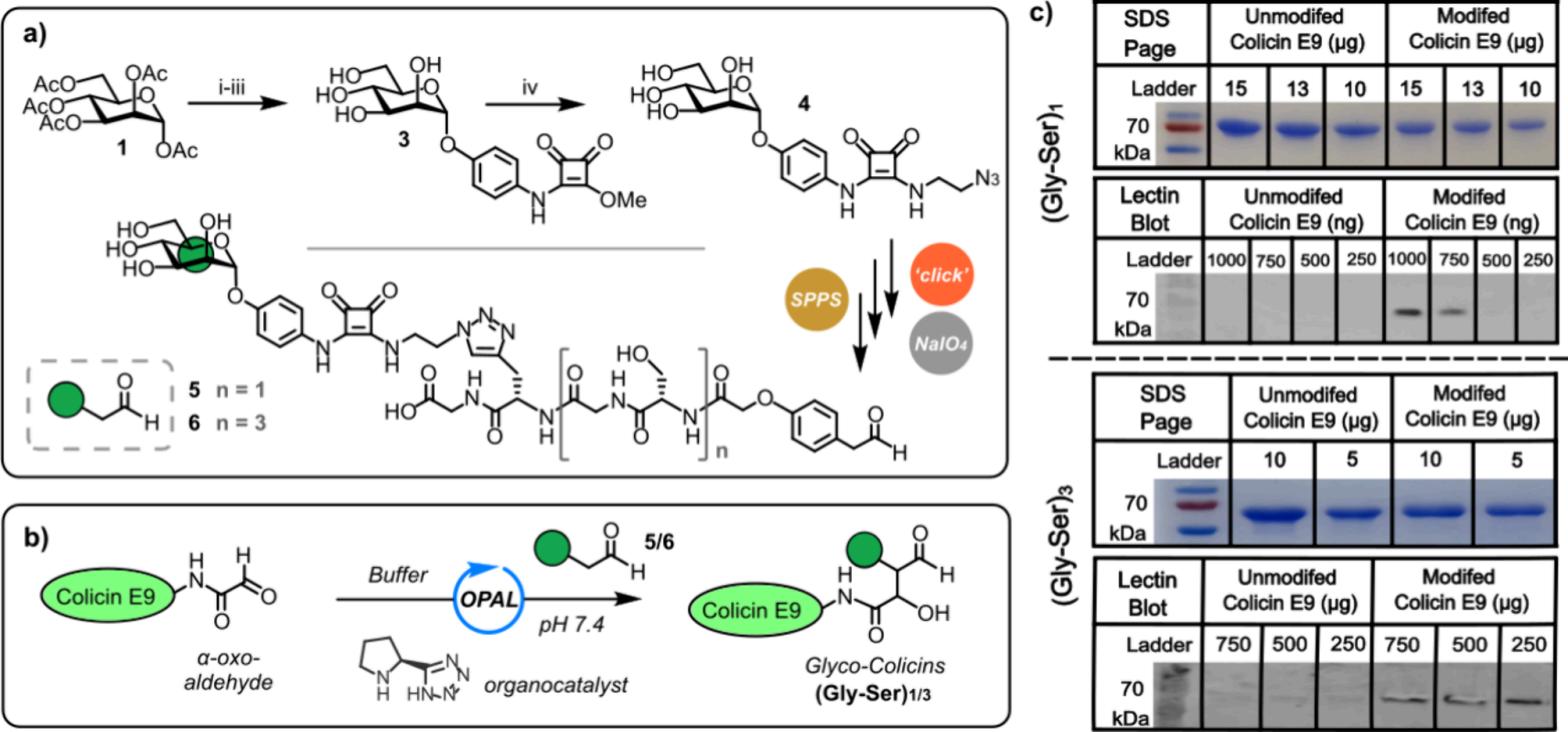
(a) The synthesis of novel mannoside azide **4** from mannose pentaacetate **1**. Reagents and conditions: (i) BF_3_·OEt_2_, 4-nitrophenol, dry DCM, 0 °C to rt, 60 h (43%); (ii) NaOMe, MeOH, rt, 20 min, then Pd/C, H_2_, dry MeOH, rt, 12 h, (over two steps 53%); (iii) dimethyl squarate, dry MeOH, rt, 4 h, (84%); (iv) 1-amino-2-azidoethane NEt_3_, MeOH, rt, 16 h, (67%). (b) A schematic depiction of proline tetrazole OPAL bioconjugation of α-oxo colicin E9 with mannose-bearing OPAL probes **5** and **6**. (c) SDS-PAGE gels and conconavalin A lectin blots analysis of the mannose-presenting colicin conjugates demonstrates successful bioconjugation.

Mannose azide **4** was synthesized via a BF_3_·OEt_2_-mediated glycosylation of d-mannose pentaacetate **1** with 4-nitrophenol to afford α-aryl mannoside **2** in a 53% yield following purification by recrystallization (Figure 4a). Subsequent deacetylation of this protected sugar was followed by the hydrogenation of the nitro group to an amine group, which was followed by squarate ester coupling to afford mannoside **3**. As the installation of a sugar into an OPAL probe had not previously been attempted, we chose to make our α-mannose-presenting squarate mannoside amenable to derivatization through azide–alkyne “click” reactions via the addition of 1-amino-2-azidoethane to afford the novel azide containing mannoside squarate **4**. Two OPAL probe precursor peptides with different serine-glycine Gly-Ser linker lengths (*n* = 1 or 3) were then synthesized via solid-phase peptide synthesis (SPPS) as we anticipated linker length and flexibility may influence the conformational entropy and, therefore, the change in free energy upon binding. The Gly-Ser spacer units were chosen as they enable subtle tailoring of linker length in SPPS while maintaining solubility. These precursor peptides featured an l-propargylglycine residue at their C-termini, which we selectively conjugated to our versatile mannose azide **4** prior to resin cleavage in an adaption of the on-resin Cu-catalyzed “click” method of Sewald and co-workers.^51^ Following cleavage from the resin, a stoichiometric quantity of NaIO_4_ was then used to cleanly and selectively convert the more reactive 1,2-amino alcohol motif present in the OPAL probe precursor peptides **S8** and **S9** to an aldehyde motif without oxidizing the 1,2-diol of the mannose motif,^52^ thereby affording two activated α-mannose-presenting OPAL probes **5** and **6** for bioconjugation (Figure 4a). After freshly installing N-terminal α-oxo aldehyde functionality into colicin E9, the OPAL probes **5** and **6** were used to construct two α-mannose-presenting glyco-colicins (Gly-Ser)_1_ and (Gly-Ser)_3_ with different length linkers (Figure 4b). The success of OPAL conjugation was confirmed through analysis by SDS-PAGE and lectin blotting with concanavalin A (a lectin that binds to terminal α-mannose units) (Figure 4c).^53^ OPAL bioconjugation of probes **5** and **6** to colicin E9 was evident in the lectin blots with no lectin binding to unmodified colicin E9 observed.

To investigate whether the glyco-colicins were capable of inducing aggregation of *E. coli*, the BW25113 wt strain was cultured in static, anaerobic conditions using glycerol as a primary carbon source in place of glucose to promote phase on transitions resulting in *fimH* expression. Incubation of the bacteria with increasing concentrations of both (Gly-Ser)_1_ and (Gly-Ser)_3_ mannose glyco-colicins was then performed with concentration-dependent bacterial aggregation observed by confocal microscopy for both linker lengths (Figure 5) with no aggregation observed when Δ*fimH* and Δ*btuB* knockout strains were used (Figure S63). The extent of cell aggregation in each sample was quantified by measuring aggregate particle dimensions, surface area and two cross-sectional measurements (termed “width” and height”) across three experimental replicates in wt, Δ*fimH*, and Δ*btuB* cells in the presence and absence of 1 mM of both mannose glyco-colicins (Figure 6a,b). Increasing levels of aggregation were observed in cells incubated with increasing concentrations (10 μM, 100 μM, and 1 mM) of both conjugates, in contrast to Δ*fimH* and Δ*btuB* where no significant aggregation was observed irrespective of conjugate concentration (Figures S63 and S64). The extent and BtuB/FimH dependence of aggregation when using 1 mM glyco-colicin is also clearly evident when the ratio of number of aggregates/total objects (aggregates + single cells) is counted and plotted (Figure 6c). Quantitative assessment of aggregation further reinforced this observation demonstrating unequivocally the efficacy of the two conjugates trigger aggregation in cells presenting FimH and BtuB at their surface with statistically significant positive shifts quantified via application of a Mann–Whitney U test to subject populations in the aggregate surface width, height, and area distributions (Tables S1–S3) observed for wt cells in the presence of 1 mM of both conjugates (Gly-Ser)_1_. In contrast, no statistically significant change in Δ*fimH* and Δ*btuB* aggregate surface area distributions was observed upon incubation with 1 mM glyco-colicin. Notably, a statistically significant larger aggregate width, height, and area size was also observed for the shorter (Gly-Ser)_1_ compared with the longer (Gly-Ser)_3_ linker, potentially indicative of a greater conformational entropy penalty when the more flexible (Gly-Ser)_3_ glyco-colicin is incorporated into an aggregate. Greater loss of entropic freedom might be anticipated for even longer linkers, which may also be able to bind BtuB and FimH on the same bacterium in a cis-fashion.

**Figure 5:**
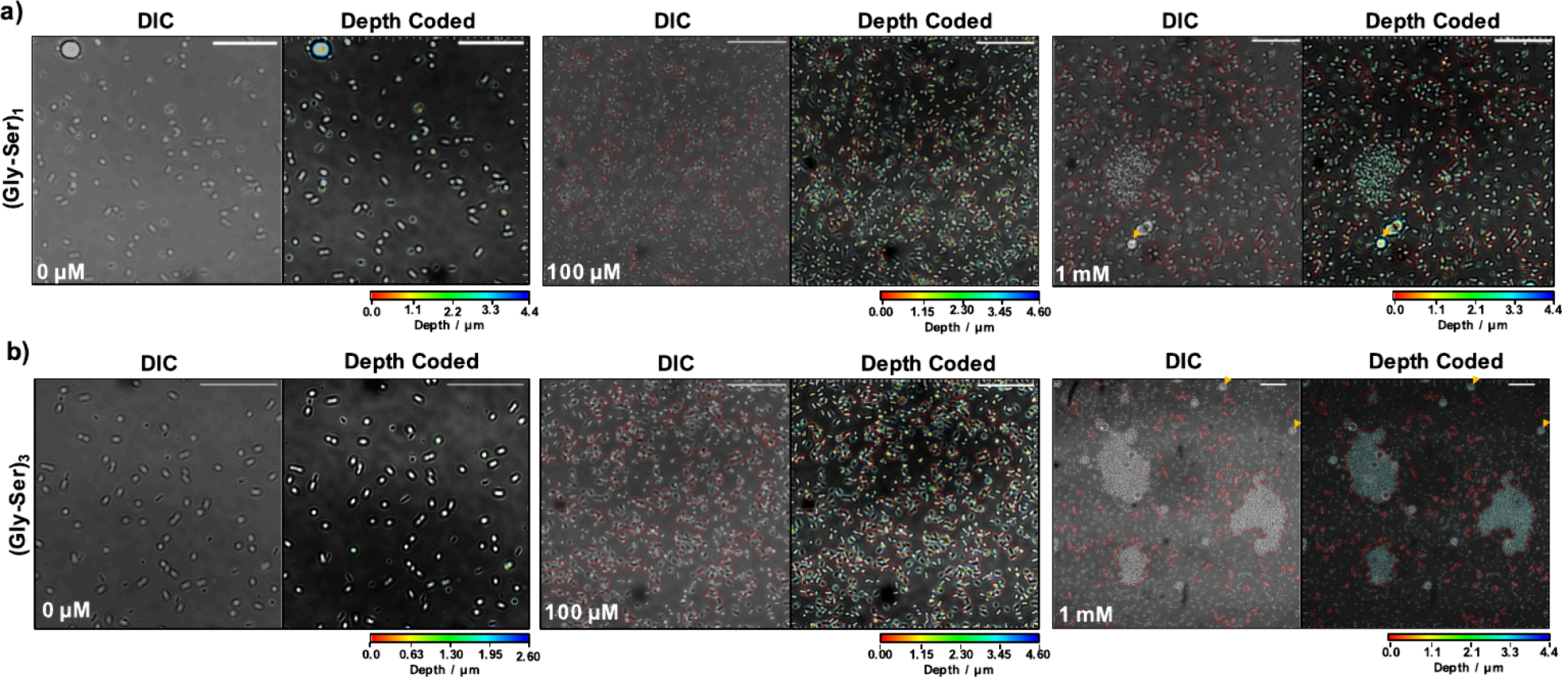
DIC and fluorescence confocal microscopy images of samples of *E. coli* BW25113 incubated with increasing concentration of mannose-presenting glyco-colicins (a) (Gly-Ser)_1_ or (b) (Gly-Ser)_3_ with aggregates outlined with red dotted lines. Scale bars, 20 μm; orange arrow = 5 μm silica spacer beads.

**Figure 6:**
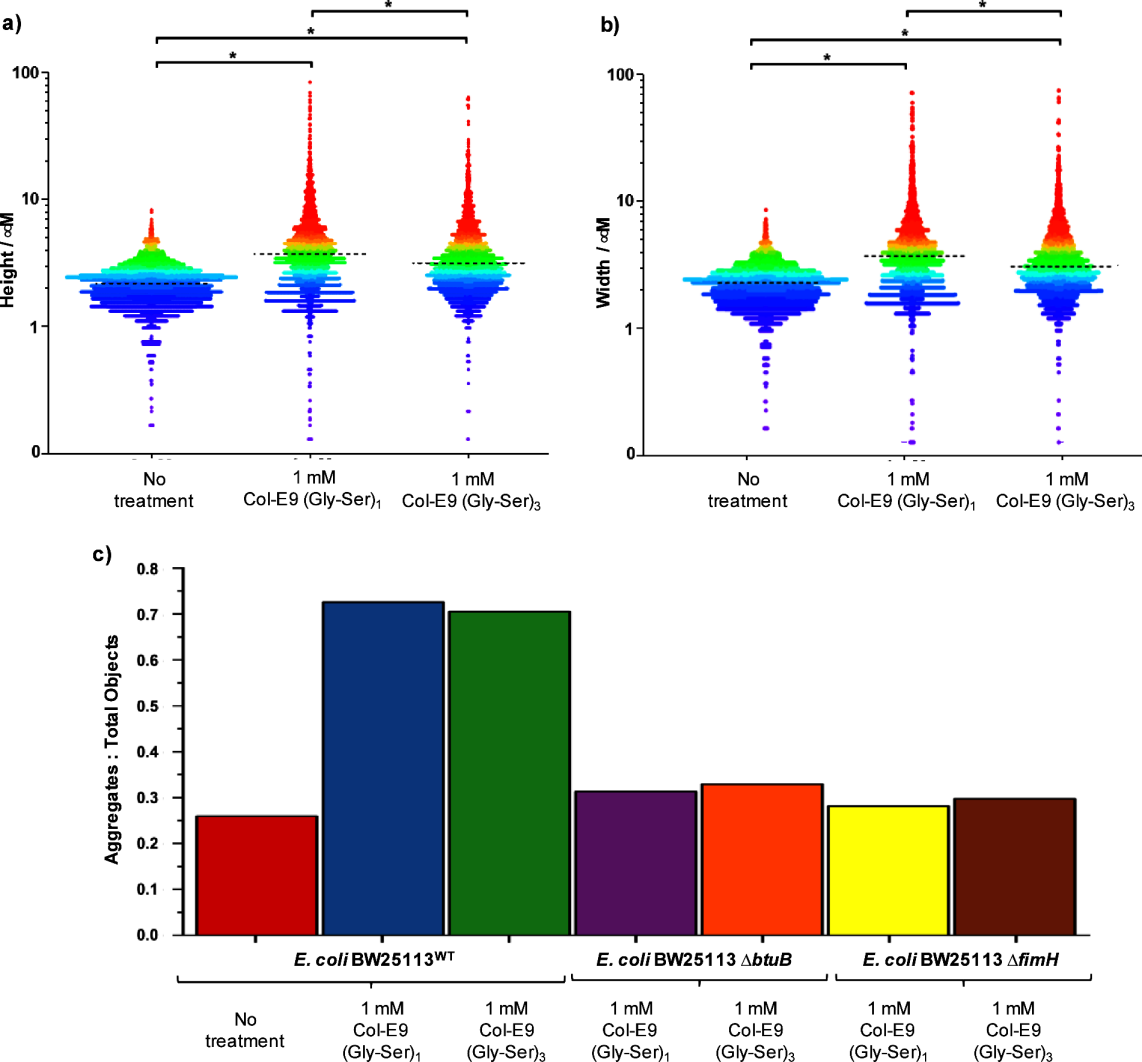
Swarm plots demonstrating incubation with 1 mM Man-Col-E9(Gly-Ser)_1_ or Man-Col-E9(Gly-Ser)_3_-induced aggregation in *E. coli* K12 substr. BW25113 wt cells. Incubation triggered statistically significant positive shifts in the cell/cell aggregate (a) height and (b) width distributions derived from analysis of fluorescence confocal microscopy images across three experimental replicates for each condition using ZEN Blue/ImageJ software (>10 images per replicate). *n*[0 mM Col-E9] = 3943, median height = 2.2 μm, median width = 2.3 μm; *n*[1 mM Col-E9(Gly-Ser)_1_] = 3648, median height = 3.7 μm, median width = 3.7 μm; *n*[1 mM Col-E9(Gly-Ser)_3_] = 3753, median height = 3.2 μm, median width = 3.1 μm. Each point plotted on the swarm plot represents an individual measurement with dotted lines representing median values for the relevant condition.*p < 0.05. (c) Bar chart depicting ratios of aggregates: total objects (single cells + aggregates) counted in confocal microscopy experiments for *E. coli* K12 substr. BW25113 wt cells, *E. coli* BW25113 *ΔbtuB*, and *E. coli* BW25113 *ΔfimH* when incubated with no treatment, 1 mM Col-E9(Gly-Ser)_1_, or 1 mM Col-E9(Gly-Ser)_3._.

In conclusion, we demonstrate that OPAL bioconjugation can be successfully used to append functional handles, such as biotin and fluorescein, to colicin proteins. Furthermore, OPAL ligation does not preclude the binding of colicin E9 to its target BtuB receptor on the surface of *E. coli*, as evidenced by fluorescent labeling of living bacterial surfaces characterized using fluorescence confocal microscopy and 3D-SIM. This work also establishes that mannose-presenting glyco-colicins can induce autoaggregation in an adherent *E. coli* strain, which was validated using fluorescence confocal microscopy and quantified statistical analyses of aggregate sizes. Bacterial autoaggregation is a complex process that occurs primarily through either a depletion aggregation mechanism, an entropy-driven process that results in characteristically ordered lateral alignment of bacteria, or a bridging aggregation mechanism where adhesins or other molecules bind neighboring cells together.^54,55^ The latter mechanism results in less-ordered aggregates composed of multiple layers of cells resembling a “stacked-brick” pattern and is characteristic of enteroaggregative *E. coli*,^55^ a diarrheagenic pathogenic human strain in which neighboring cells are held together by aggregative adhesive fimbriae.^56^ In this study, when using glyco-colicins, similar fimbriae-dependent “stacked-brick”-like disordered aggregation is observed, which we hypothesize is a result of simultaneous binding of conjugates to the BtuB outer membrane receptor on one *E. coli* bacterium and a FimH adhesin on an adjacent bacterium in a trans-fashion. Thus, highlighting how glyco-colicins can potentially mimic native bacterial autoaggregation mechanisms. Additionally, although formation of large aggregates was observed when using 1 mM glyco-colicins, microscopy studies provide evidence of aggregation at micromolar concentration, which are below the concentration required to reduce bacterial loads in mouse bladder studies using monovalent mannosides,^11^ thereby underlining potential future application of glyco-colicins as antiadhesion therapies. However, pharmacokinetics would need to be carefully considered if oral administration was envisioned, with mannose glycoside containing conjugates likely susceptible to the action of glycosidases.^57,58^ An alternative application for glyco-colicins may instead be as a catheter coating or pretreatment to combat catheter-associated infections, which currently account for 75% of all UTIs.^59^

## Supporting Information Available

Synthesis and bioconjugation methodologies, full compound characterization, NMR spectra, LCMS spectra, and microscopy data (PDF)
