# Supplementary material for: Mannose-Presenting “Glyco-Colicins” Convert the Bacterial Cell Surface into a Multivalent Adsorption Site for Adherent Bacteria
Source: JACS Au. 2024 Jun 12;4(6):2122–9. doi: 10.1021/jacsau.4c00365 (PMC11200225; doi:10.1021/jacsau.4c00365)
Supplement: Supplementary file 1 — au4c00365_si_001.pdf [file au4c00365_si_001.pdf]

Supporting information

Mannose Presenting 'Glyco-Colicins' Convert the Bacterial Cell Surface into a Multivalent Adsorption Site for Adherent Bacteria

Natasha E. Hatton,[a] Joe Nabarro,[a] Nicholas D. J. Yates,[a] Alison Parkin,[a] Laurence G. Wilson,[b] Christoph G. Baumann*[c] and Martin A. Fascione*[a]

[a] Department of Chemistry, University of York, York, YO10 5DD, UK; [b] Department of Physics, University of York, York, YO10 5DD, UK; [c] Department of Biology, University of York, York, YO10 5DD, UK

***Corresponding author:**

martin.fascione@york.ac.uk

christoph.baumann@york.ac.uk

General Appendix

Non-chapter specific experimental procedures/information

The use of dry solvents is stated in individual methodologies. All dry solvents used were dried according to standard methods and solvents used for flash chromatography purposes were not dried prior to use. All chemical synthesis reactions were carried out in oven-dried glassware. Thin layer chromatography was carried out on Merck silica gel 60 F_{254} pre-coated aluminium foil sheets and were visualised using UV light (254 nm) and stained with either a 5% sulphuric acid in EtOH stain (for sugars), Ninhydrin stain (for amines and BOC protected amines) and a PPh_3 stain followed by a Ninhydrin stain (for azides).

Spectroscopic and spectrometric instruments and standard practices

^{1}H-NMR (500 MHz) and ^{13}C-NMR (126 MHz) experiments were conducted using a Bruker AVIIIHD 500 instrument at The University of York Centre for Magnetic Resonance. ^{1}H-NMR (400 MHz) and ^{13}C-NMR (101 MHz) experiments were conducted using a JEOL 400 instrument at The University of York Centre for Magnetic Resonance. Me_4Si was often used as an internal standard at either 0.1% or 1% when using chloroform-d as a NMR solvent. Multiplicities are given as singlet (s), doublet (d), triplet (t), doublet of doublets (dd), doublet of doublet of doublets (ddd), triplet of doublets (td), quartet of doublets (qd) or multiplet (m). Resonances were assigned using HH-COSY and CH-HSQC. All NMR chemical shifts (δ) were recorded in ppm and coupling constants (J) are reported in Hz. Topspin 4.0.6 and MestReNovax64 were primarily used for processing the spectral data.

Fourier transform infrared (FTIR) spectra were recorded on a PerkinElmer UATR 2 spectrometer using the attenuated total reflectance (ATR) technique. Optical rotations were measured using a Bellingham and Stanley ADP 450 Automatic Digital Peltier Controlled Polarimeter equipped with a 589 nm LED. Concentration is denoted as "c" and was calculated as grams per 100 millilitres (g / 100 mL) whereas the solvent is indicated in parenthesis (c, solvent).

ESI-MS experiments used for the characterisation of chemically synthesised molecules were conducted using a Bruker micrOTOF mass spectrometer coupled to an Agilent 1200 series LC system. More details regarding the ESI-LC/MS analysis of OPAL probes and enzymatically synthesised sugars can be found below.

LCMS Procedures

ESI-LC/MS of OPAL probes

LC-MS analysis was performed on a Dionex UltiMate® 3000 Ci Rapid Separation LC system equipped with an UltiMate® 3000 photodiode array detector probing at 250-400 nm coupled to a HCT ultra ETD 11 (Bruker Daltonics) ion trap spectrometer, using Chromeleon® 6.80 SR12 software (ThermoScientific), Compass 1.3 for esquire HCT build 581.3, esquire Control version 6.2, Build 62.24 software (Bruker Daltonics) and Bruker compass HyStar 3.2-SR2, HyStar version 3.2, Build 44 software (Bruker Daltonics) at The University of York Centre of Excellence in Mass Spectrometry (CoEMS). Data analysis was performed using ESI compass 1.3 DataAnalysis, Version 4.4 software (Bruker Daltonics). All peptide/protein mass spectrometry was conducted in positive ion mode unless otherwise stated. Samples made for LCMS were made using a 50% (v/v) HPLC grade water/acetonitrile, 1% formic acid solution.

General analysis of protected and active OPAL probes (minus probe protected-(Gly-Ser_3)-OPAL probe)
Samples were analysed using an Accucore™ C18 HPLC Columns 2.6 µm 2.1 x 150 mm reverse-phase column. Water + 0.1% formic acid by volume (solvent A) and acetonitrile + 0.1% formic acid (solvent B) were used as a mobile phase at a flow rate of 300 µL min^{-1} at RT. A multi-step gradient of 7.5 min was programmed as follows: 95% A for 1.0 min, followed by a linear gradient to 95% B over 6.5 min, followed by 95% B for an additional 1.0 min. A linear gradient to 95% A was used to equilibrate the column.

Other samples (notably probe protected-(Gly-Ser_3)-OPAL probe)
These samples were analysed without the use of a column. Analysis was performed at RT, HPLC-grade water with 0.1% (v/v) formic acid (solvent A) and acetonitrile with 0.1% (v/v) formic acid (solvent B) were used as the mobile phase at a 1:1 ratio over the course of 3 min as follows: 50 µL min^{-1} to 250 µL min^{-1} for 1 min, 250 µL min^{-1} for 1 min, followed by 1000 µL min^{-1} for 1 min.

SDS-PAGE gel and lectin blotting protocols

SDS-PAGE gel protocol

10% SDS-PAGE gels were used to analyse bioconjugation. These 10% SDS-PAGE gels were poured in-house using a specialised kit, and were made using the following protocol: H_2O (4 mL) was mixed with 2.5 mL of resolving buffer (1.5 M Tris-HCl, 0.4% SDS, pH 8.8). To the resultant mixture was added 30% acrylamide (3.3 mL), 20% ammonium persulfate solution (50 µL) and tetramethylethylenediamine (10 µL). The resultant mixture was briefly gently agitated to ensure mixing, and then poured into the mould and allowed to set. Once the gel had set a stacking gel was allowed to set on top of the main gel, with a comb inserted to create sample lanes. The protocol for mixing the stacking gel is as follows: H_2O (3.2 mL) was mixed with 1.3 mL of resolving buffer. To the resultant mixture was added 30% acrylamide (0.5 mL), 20% ammonium persulfate solution (12.5 µL) and tetramethylethylenediamine (8 µL). Once the SDS-PAGE gels were fully prepared, complete with stacker gel and sample lanes, the SDS-PAGE gels were ready to be loaded with samples. Unless otherwise stated, samples were mixed with a 5 × concentrated reducing buffer (10% SDS, 10 mM 2-mercaptoethanol, 20% glycerol, 200 mM Tris-HCl pH 6.8, 0.05% bromophenol blue) and boiled for 5 min prior to running on the SDS-PAGE gel. The molecular weight markers used were PageRuler™ Plus Prestained Protein Ladder (ThermoFisher Scientific). Each gel was run at 200 V for 30-80 min in SDS running buffer (25 mM Tris, 192 mM Gly, not pH adjusted). After being run, SDS-PAGE gels were fixed via gel emersion in a fixing solution (40% water, 50% EtOH, 10% AcOH) which was gently rocked for 60 minutes. For experiments in which the SDS PAGE gel was subsequently stained with Coomassie stain, the fixed gels were then immersed in a solution of 0.1% Coomassie Brilliant Blue R-250 (in 40% water, 50% EtOH, 10% AcOH), and the solution brought to the boil in a microwave, before being gently rocked at room temperature for a further 20 min. The Coomassie stained gels would then be destained via emersion in a destaining solution (50% water, 40% EtOH, 10% AcOH), which was periodically replaced with dye-free destaining solution as the destaining process progressed.

Lectin blot protocol
The blots were assembled using 12 layers of blotting paper and one layer of nitrocellulose membrane which has previously been soaked in transfer buffer (25 mM Tris-HCl pH = 8.3, 192 mM glycine, 20% MeOH) for 10 minutes and an SDS-PAGE gel which had been run but not stained. The blots were assembled in the following order - 6 layers blotting paper, nitrocellulose membrane, 10% SDS-PAGE

gel and 6 layers of blotting paper. The gels were transferred to the nitrocellulose membranes using a Trans-Blot®Turbo™ transfer system running at 1.3 A constant; up to 25 V for 30 minutes. The membranes were then incubated in 1 x PBS and 2% tween® 20 for 5 minutes with gentle rocking followed by two further washes with 1 x PBS (5 minutes with gentle rocking). The membranes were then incubated with 10 mL 1 x PBS containing 0.05% tween® 20 (Sigma Aldrich), 1 mM $CaCl_2$, 1 mM $MnCl_2$ and 1 mM $MgCl_2$ and 5 pM lectin peroxidase (Sigma Aldrich). The blots were incubated at RT for 16 h and then incubated with 4 mL Amersham (Cytiva) for five minutes. Finally the blots were imaged using a Syngene G:BOX Chemi XRQ equipped with a Synoptic 4.0 MP camera, with GeneSyn software (Version 1.5.70).

Colicin E9 sequence, production and purification

Plasmid sequence

Y324C L447C K469C colicin E9

```
MSGGDGRGHN TGAHSTSGNI NGGPTGIGVS GGASDGSGWS SENNPWGGGS GSGIHWGGGS
GRGNGGGNGN SGGGSGTGGN LSAVAAPVAF GFPALSTPGA GGLAVSISAS ELSAAIAGII
AKLKKVNLKF TPFGVVLSSL IPSEIAKDDP NMMSKIVTSL PADDITESPV SSLPLDKATV
NVNVRVVDDV KDERQNISVV SGVPMSVPVV DAKPTERPGV FTASIPGAPV LNISVNDSTP
AVQTLSPGVT NNTDKDVRPA GFTQGGNTRD AVIRFPKDSG HNAVYVSVSD VLSPDQVKQR
QDEENRRQQE WDATHPVEAA ERNCERARAE LNQANEDVAR NQERQAKAVQ VYNSRKSELD
AANKTLADAI AEIKQFNRFA HDPMAGGHRM WQMAGLKAQR AQTDVNNKQA AFDAAAKEKS
DADAALSAAQ ERRKQKENKE KDAKDKCAME SKRNKPGKAT GKGKPVGDCW LDDAGKDSGA
PIPDRIADKL RDKEFKSFDD FRKAVWEEVS KDPELSKNLN PSNKSSVSKG YSPFTPKNQQ
VGGRKVYELH HDKPISQGGE VYDMDNIRVT TPKRHIDIHR GK*
```

Protein information

Number of amino acids: 582

Molecular weight: 61,451 g/mole

Theoretical pI: 8.46

Extinction coefficient at 280 nm (oxidised): 46,075 $M^{-1}cm^{-1}$

Protein production and purification

Two 500 mL cultures were grown and combined for purification.

An *E. coli* BL21 (DE3) (New England Biolabs) transformant harbouring the expression plasmid was grown at 37 °C in 5 mL of Luria broth (LB) media (1% NaCl, 1% tryptone and 0.5% yeast extract) containing 100 µg/mL ampicillin and 0.4% w/v D-glucose for 7 h with shaking at 150 rpm. 50 mL of LB media containing 100 µg/mL ampicillin and 0.4% w/v D-glucose was inoculated with the starter culture and grown overnight at 37 °C with shaking at 150 rpm. 500 mL of LB media containing 100 µg/mL ampicillin and 0.4% w/v D-glucose was inoculated with the overnight culture and was grown to an OD_{600} = 0.6. The expression was induced by 1 mM isopropyl β-D-thiogalactopyranoside and was incubated for 18 h at 37 °C with shaking at 150 rpm. The cells were harvested by centrifugation 6000 rpm for 12 minutes at 4 °C. The resulting cell pellet was resuspended in 35 mL of 20 mM K-phosphate pH 7.0, 0.5 M NaCl, 1 mM PMSF and lysozyme (add ~10 mg/35 ml) and disrupted by sonication (soniprep 150) (30 s pulses for 10 minutes). The supernatant was collected by centrifugation 20,000 × g for 30 minutes at 4 °C and filtered through a 0.45 µm pore syringe and applied to a HisTrap HP column (5 mL, Cytiva) equilibrated with 20 mM K-phosphate pH 7.0, 0.5 M NaCl, 5 mM imidazole using an AKTA start (Cytiva). The protein was eluted using a linear gradient of 20 mM K-phosphate pH 7.0, 0.5 M NaCl, 5 mM imidazole and 20 mM K-phosphate pH 7.0, 0.5 M NaCl, 6 M guanidine HCl. Fractions containing recombinant protein (colicin E9) were pooled, dialyzed against 20 mM K-phosphate pH 7.0, 0.5 M NaCl and concentrated (Sartorius vivaspin6 30,000 MWCO). The recombinant protein was further purified using gel filtration (Hiload 16/600, superdex 200pg, Cytiva) equilibrated with 20 mM K-phosphate pH 7.0, 0.5 M NaCl. The fractions containing recombinant protein (colicin E9) were

pooled and concentrated (Sartorius vivaspin6 30,000 MWCO). The protein concentration was determined spectrophotometrically at 280 nm using an extinction coefficient of 46,075. The procedure yielded 4.78 mg of purified colicin E9.

Formation of a biotin-linked colicin E9 conjugate

Synthesis of a biotin-linked colicin E9 conjugate

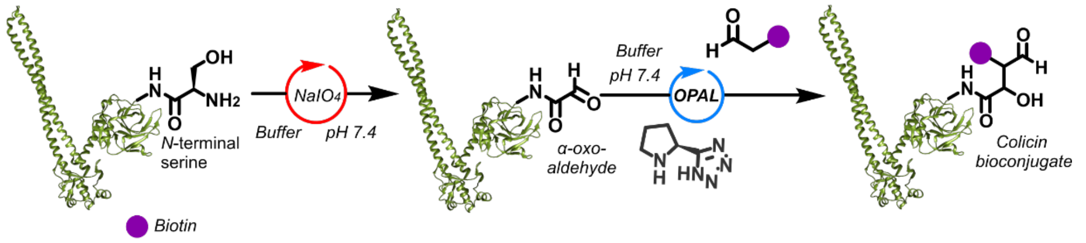

Scheme S1. Depiction of initial $NaIO_4$ oxidation of colicin E9 (PDB; 5EW5[1]) followed by OPAL ligation to a biotin probe

A solution of colicin E9 (100 µL of 52.6 µM stock in 25 mM PB pH 7.5) was charged with L-methionine (0.5 µL of 66 mM stock in 0.1 M PB, 0.1 NaCl, pH 7.0) and $NaIO_4$ (0.5 µL of 33 mM stock in 0.1 M PB, 0.1 NaCl, pH 7.0). The solution was mixed by gentle pipette tip swirling and allowed to sit on ice in the dark for 4 minutes. The reaction mixture was immediately purified using a PD SpinTrap G25 desalting column (GE Healthcare Life Sciences), eluting into 100 µL of 25 mM PB pH 7.5. The reaction was charged with (S)-(-)-5-(2-pyrrolidinyl)-H-tetrazole (2.5 µL of 200 mM stock in 25 mM PB pH 7.5) and biotin OPAL probe[2, 3] (5 µL of 4 mM stock in 25 mM PB pH 7.5). The solution was mixed via pipette tip swirling and incubated for 1 h at 37 °C. The reaction mixture was purified using a PD SpinTrap G25 desalting column (GE Healthcare Life Sciences), eluting into 20 mM K phosphate, 500 mM NaCl pH 7.0 for analysis and further manipulation.

Raw SDS-PAGE gel and Western blot

SDS-PAGE

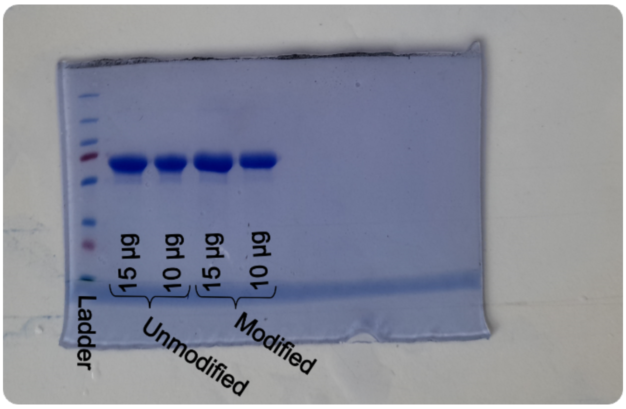

Figure S1. SDS PAGE gel analysis of the biotin-linked colicin E9 conjugate

Western blot

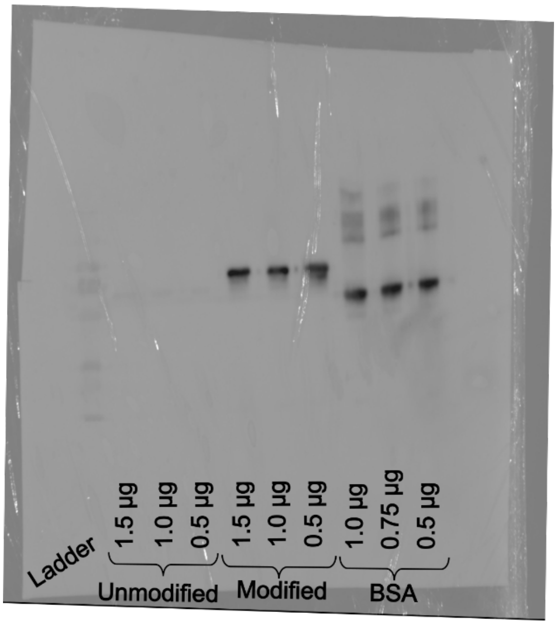

Figure S2. Western blot analysis of the biotin-linked colicin E9 conjugate. Note a bovine serum albumin (BSA) standard was used in this gel

Formation of a Fluorescein-linked colicin E9 conjugate

Synthesis of the Fluorescein OPAL probe

General scheme

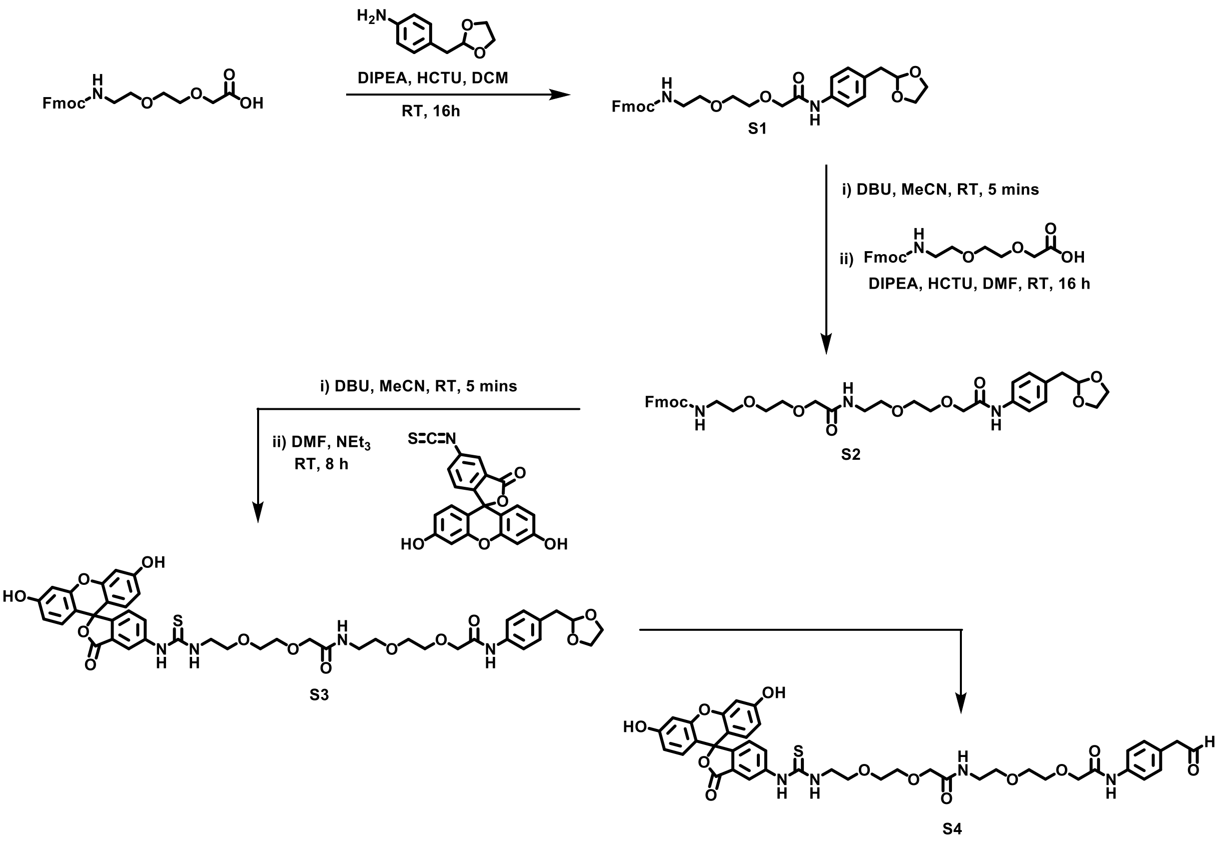

Scheme S2. Synthesis scheme for 2-((1-((3',6'-dihydroxy-3-oxo-3H-spiro[isobenzofuran-1,9'-xanthen]-5-yl)amino)-10-oxo-1-thioxo-5,8,14-trioxa-2,11-diazahexadecan-16-yl)oxy)-N-(4-(2-oxoethyl)phenyl)acetamide **S4**

Step 1. Synthesis of (9H-fluoren-9-yl)methyl (2-(2-(2-((4-((1,3-dioxolan-2-yl)methyl)phenyl)amino)-2-oxoethoxy) ethoxy)ethyl)carbamate S1

To a solution of {2-[2-(Fmoc-amino)ethoxy]ethoxy}acetic acid (193 mg, 0.50 mmol) in DCM (5 mL) was added DIPEA (172 µL, 128 mg, 1.00 mmol) and HCTU (0.206 g, 0.50 mmol) were added. The resultant solution was briefly agitated, and 2-(4-aminobenzyl)-1,3-dioxolane[2] (0.104 g, 0.58 mmol) was then added. The resultant solution was stirred overnight at rt in darkness. After this time the reaction mixture was dry-loaded onto Celite® prior to being partially purified via flash column chromatography (SiO_2, hexane → EtOAc). This yielded a pale green oil containing **S1** and a small quantity of 1,1,3,3-tetramethylurea (0.166 g, approx. 61%).

^{1}H-NMR (400 MHz, $CDCl_3$) δ_H 8.52 (br s, 1H), 7.75 (d, *J* = 7.55 Hz, 2H), 7.56 (dd, *J* = 7.45, 0.45 Hz, 2H), 7.52-7.45 (m, 2H), 7.39 (dd, *J* = 7.45, 7.45 Hz, 2H), 7.29 (ddd, *J* = 7.55, 7.45, 0.60 Hz, 2H), 7.24-7.19 (m, 2H), 5.13 (br t, *J* = 5.07 Hz, 1H), 5.00 (t, *J* = 4.72 Hz, 1H), 4.37 (d, *J* = 6.90 Hz, 2H), 4.18 (t, *J* = 6.90 Hz, 1H), 4.10 (s, 2H), 3.95-3.84 (m, 2H), 3.84-3.65 (m, 6H), 3.62 (t, *J* = 5.08 Hz, 2H), 3.47-3.37 (m, 2H), 2.90 (d, *J* = 4.71 Hz, 2H).

^{13}C-NMR (101 MHz, $CDCl_3$): δ_c 168.0, 156.6, 144.0, 141.4, 135.9, 132.6, 130.5, 127.8, 127.2, 125.2, 120.1, 120.0, 104.6, 71.3, 70.8, 70.4, 70.2, 66.9, 65.1, 47.3, 40.9, 40.3.

FT-IR (ATR) (υmax/cm^{-1}): 3330 (N-H stretch), 3207, 2888 (C-H stretch), 1694 (C=O stretch), 1530, 1133 (C-O stretch, ether), 833 (C-H bend, aromatic).

(ESI)HRMS: Found 569.2266, $C_{31}H_{34}N_2NaO_7^+$ requires 569.2258.

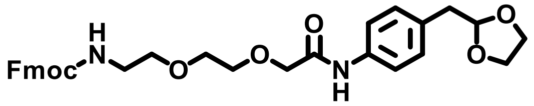

Figure S3. Structure of (9H-fluoren-9-yl)methyl (2-(2-(2-((4-((1,3-dioxolan-2-yl)methyl)phenyl)amino)-2-oxoethoxy) ethoxy)ethyl)carbamate **S1**

Hydrogen NMR Spectrum for (9H-fluoren-9-yl)methyl (2-(2-(2-((4-((1,3-dioxolan-2-yl)methyl)phenyl)amino)-2-oxoethoxy) ethoxy)ethyl)carbamate **S1**

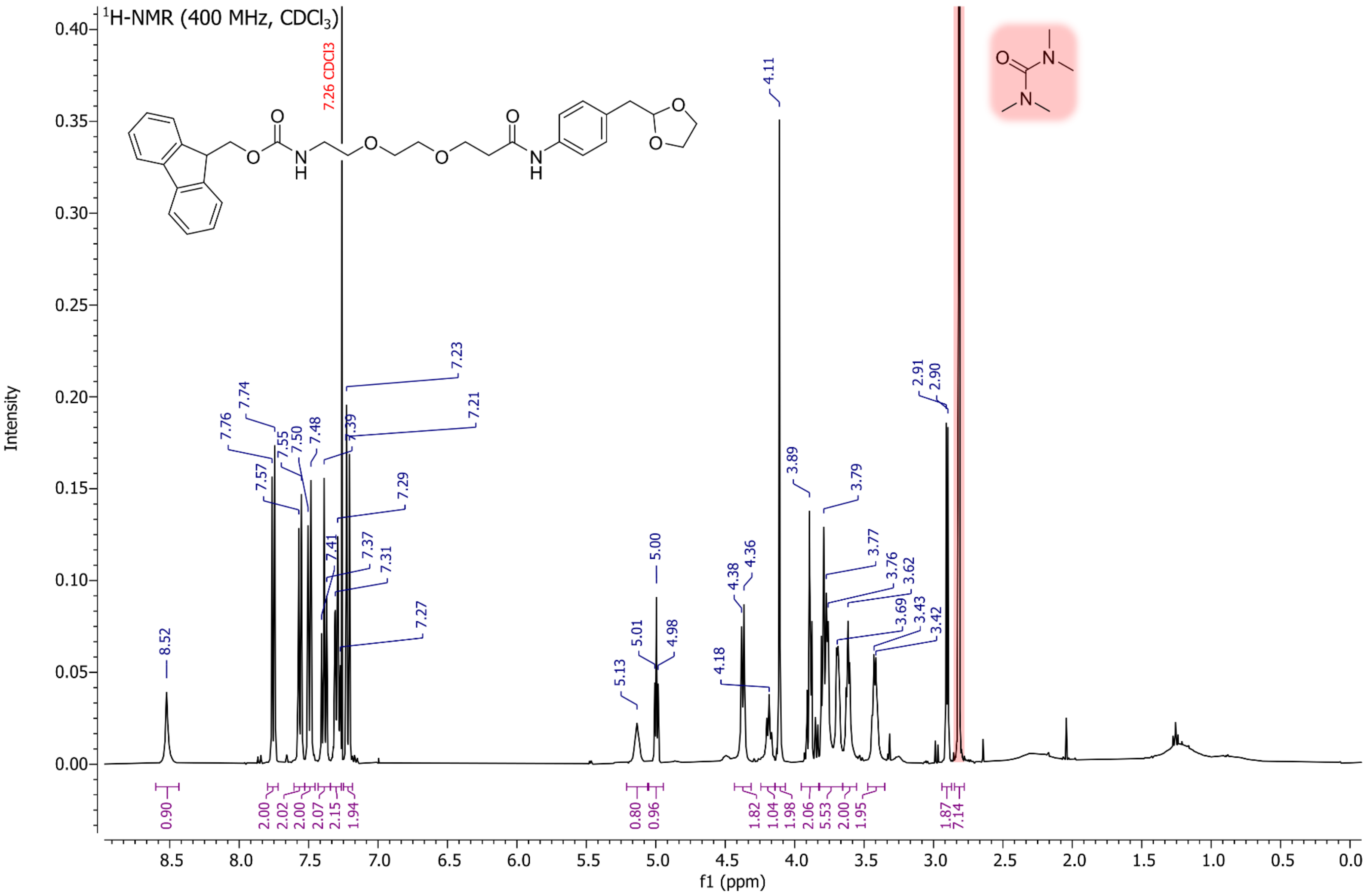

Figure S4. Hydrogen NMR of **S1**

Carbon NMR Spectrum for (9H-fluoren-9-yl)methyl (2-(2-(2-((4-((1,3-dioxolan-2-yl)methyl)phenyl)amino)-2-oxoethoxy) ethoxy)ethyl)carbamate **S1**

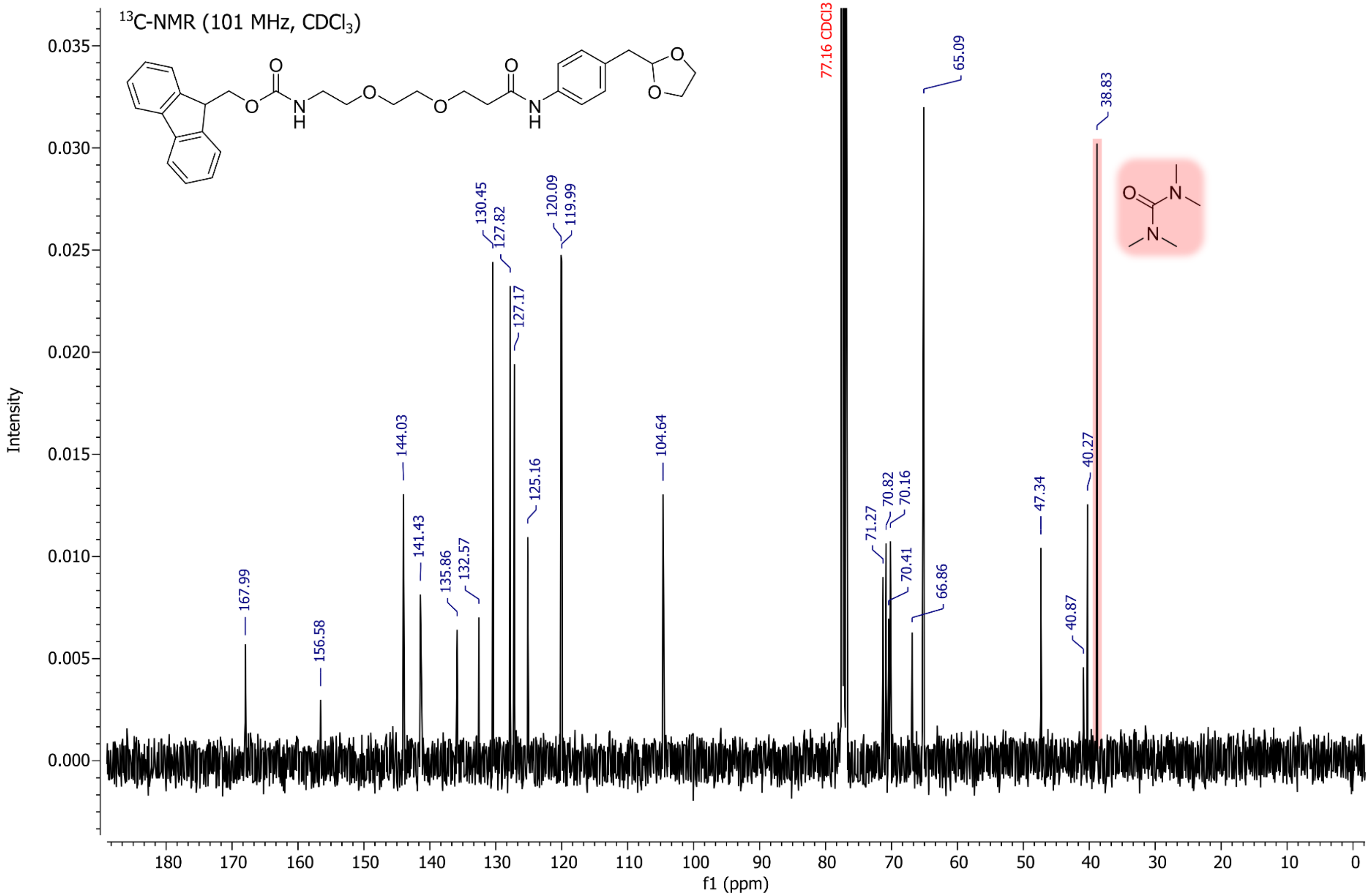

Figure S5. Carbon NMR of **S1**

Mass Spectrum for (9H-fluoren-9-yl)methyl (2-(2-(2-((4-((1,3-dioxolan-2-yl)methyl)phenyl)amino)-2-oxoethoxy) ethoxy)ethyl)carbamate **S1**

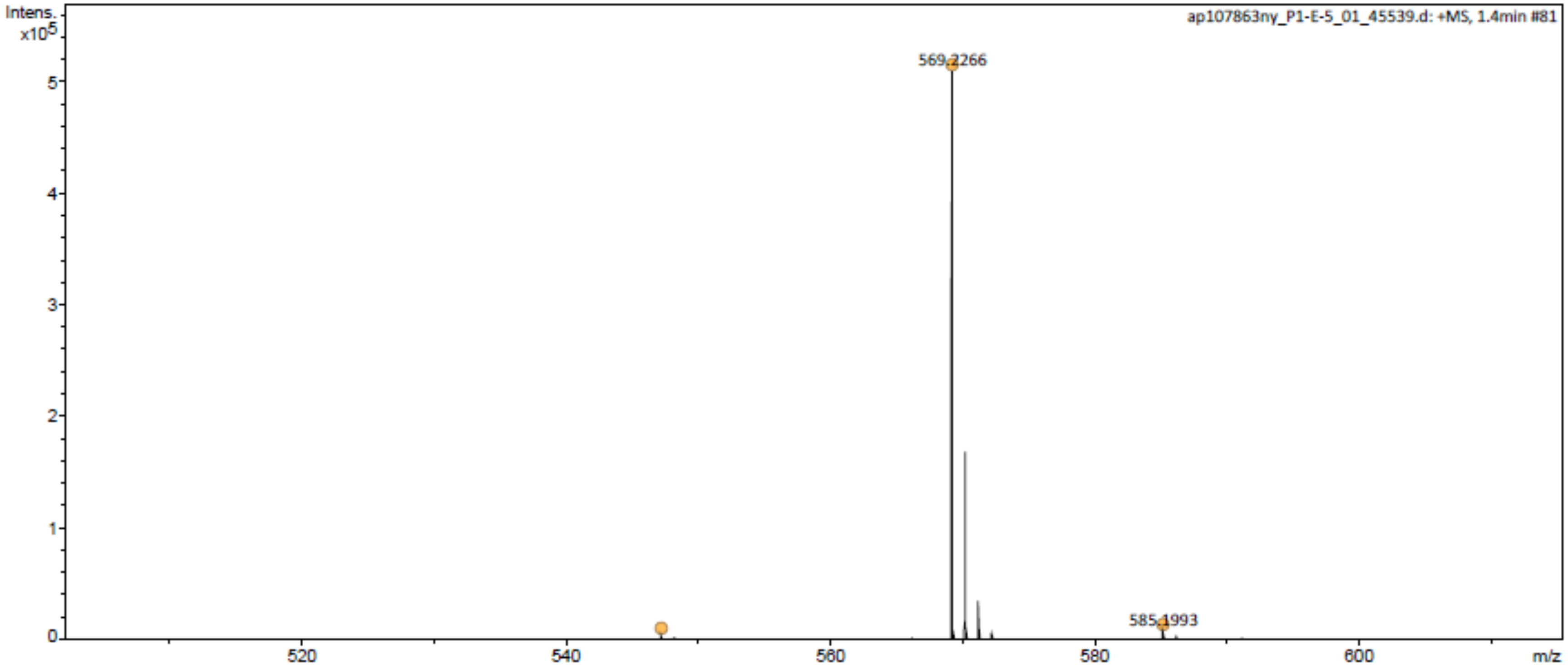

Meas. m/z	#	Ion Formula	m/z	err [ppm]	err [mDa]	mSigma	Mean err [ppm]
547.2459	1	C31H35N2O7	547.2439	-3.6	-2.0	42.3	-1.1
569.2266	1	C31H34N2NaO7	569.2258	-1.4	-0.8	9.3	-1.7
585.1993	1	C31H34KN2O7	585.1998	0.7	0.4	43.3	-1.2

Figure S6. Mass spectrum of **S1**

FT-IR (ATR) Spectrum for (9H-fluoren-9-yl)methyl (2-(2-(2-((4-((1,3-dioxolan-2-yl)methyl)phenyl)amino)-2-oxoethoxy) ethoxy)ethyl)carbamate **S1**

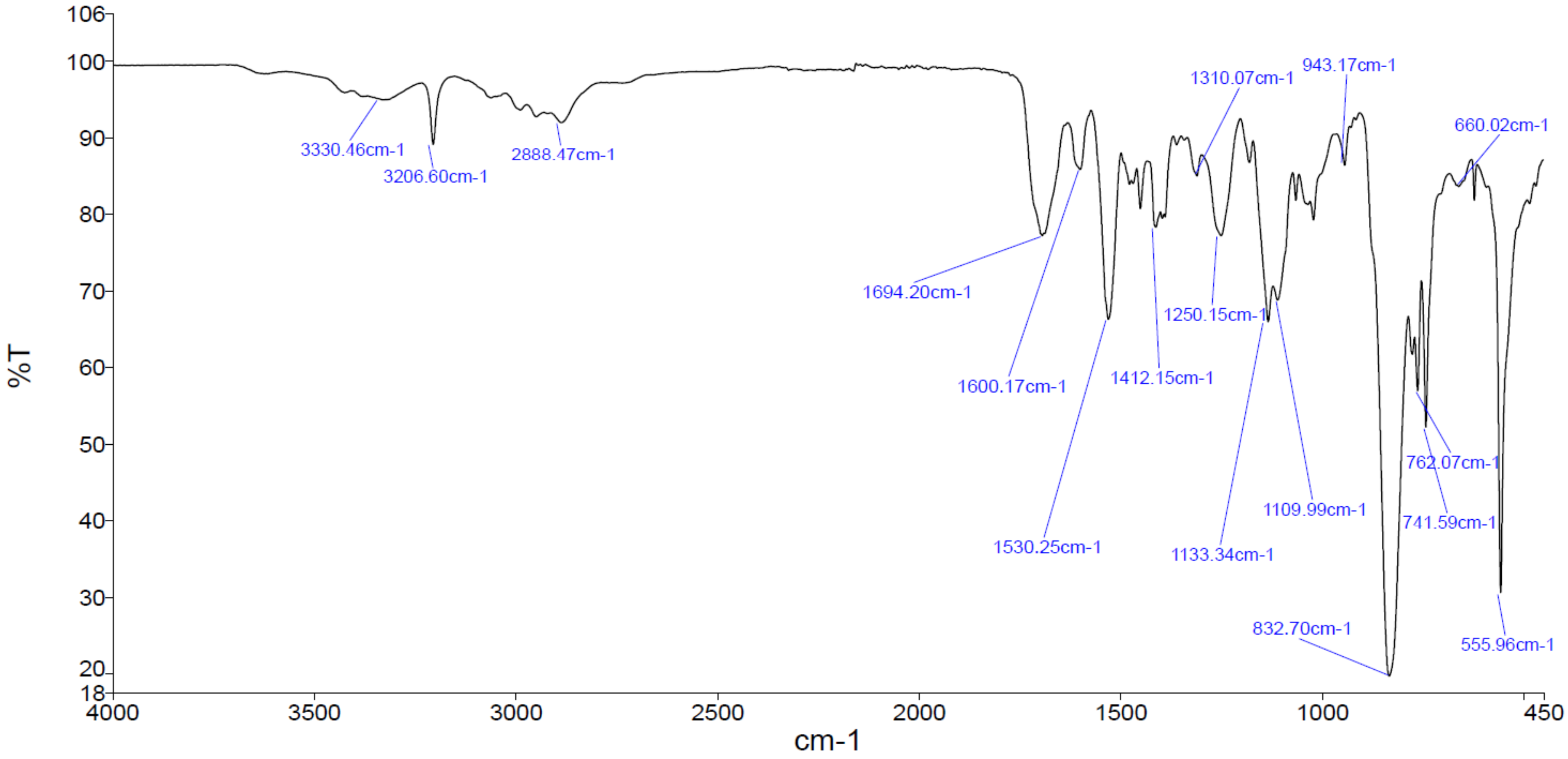

Figure S7. IR spectrum of **S1**

Step 2 Synthesis of (9H-fluoren-9-yl)methyl (17-((4-((1,3-dioxolan-2-yl)methyl)phenyl)amino)-8,17-dioxo-3,6,12,15-tetraoxa-9-azaheptadecyl)carbamate S2

To a solution of **S1** (273 mg, approx. 0.5 mmol) in MeCN (1 mL) inside a 15 mL Falcon™ tube was added DBU (100 µL, 102 mg, 0.54 mmol). The resultant solution was agitated at rt for 5 minutes, after which time diethyl ether (17 mL) was added, causing the solution to become milky due to the precipitation of an oil. Centrifugation was used to pellet the oil and the supernatant was discarded. The pelleted oil was rinsed with diethyl ether (4 mL) and was then dried *in vacuo*.

To a solution of {2-[2-(Fmoc-amino)ethoxy]ethoxy}acetic acid (193 mg, 0.50 mmol) in DMF (5 mL) was added DIPEA (172 µL, 128 mg, 1.00 mmol) and HCTU (0.206 g, 0.50 mmol) were added. The resultant solution was briefly agitated, and was then transferred to the falcon tube containing the oil derived from **S1**. The resultant solution was then agitated overnight at rt, after which time it was concentrated *in vacuo* and partially purified via flash column chromatography (SiO_2, DCM → 20% MeOH) to yield a semi-crude sample of **S2.**

^{1}H-NMR (400 MHz, Methanol-d_4) δ_H 7.78 (d, *J* = 7.53 Hz, 2H, Ar), 7.63 (d, *J* = 7.45 Hz, 2H, Ar), 7.52-7.45 (m, 2H, Ar), 7.42-7.34 (m, 2H, Ar), 7.34-7.36 (m, 2H, Ar), 7.24-7.17 (m, 2H, Ar), 4.96 (t, *J* = 4.70 Hz, 1H, dioxolane), 4.37 (d, *J* = 6.67 Hz, 2H), 4.18 (t, *J* = 6.72 Hz, 1H), 4.07 (s, 2H), 3.92 (s, 2H), 3.90-3.75 (m, 4H, dioxolane), 3.45-3.38 (m, 2H), 2.86 (d, *J* = 4.68 Hz, 2H).

(ESI)HRMS: Found 714.3011, $C_{37}H_{45}N_3NaO_{10}^+$ requires 714.2997.

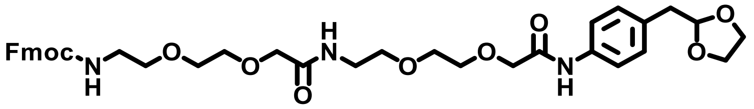

Figure S8. Structure of (9H-fluoren-9-yl)methyl (17-((4-((1,3-dioxolan-2-yl)methyl)phenyl)amino)-8,17-dioxo-3,6,12,15-tetraoxa-9-azaheptadecyl)carbamate **S2**

Hydrogen NMR Spectrum for (9H-fluoren-9-yl)methyl (17-((4-((1,3-dioxolan-2-yl)methyl)phenyl)amino)-8,17-dioxo-3,6,12,15-tetraoxa-9-azaheptadecyl)carbamate **S2**

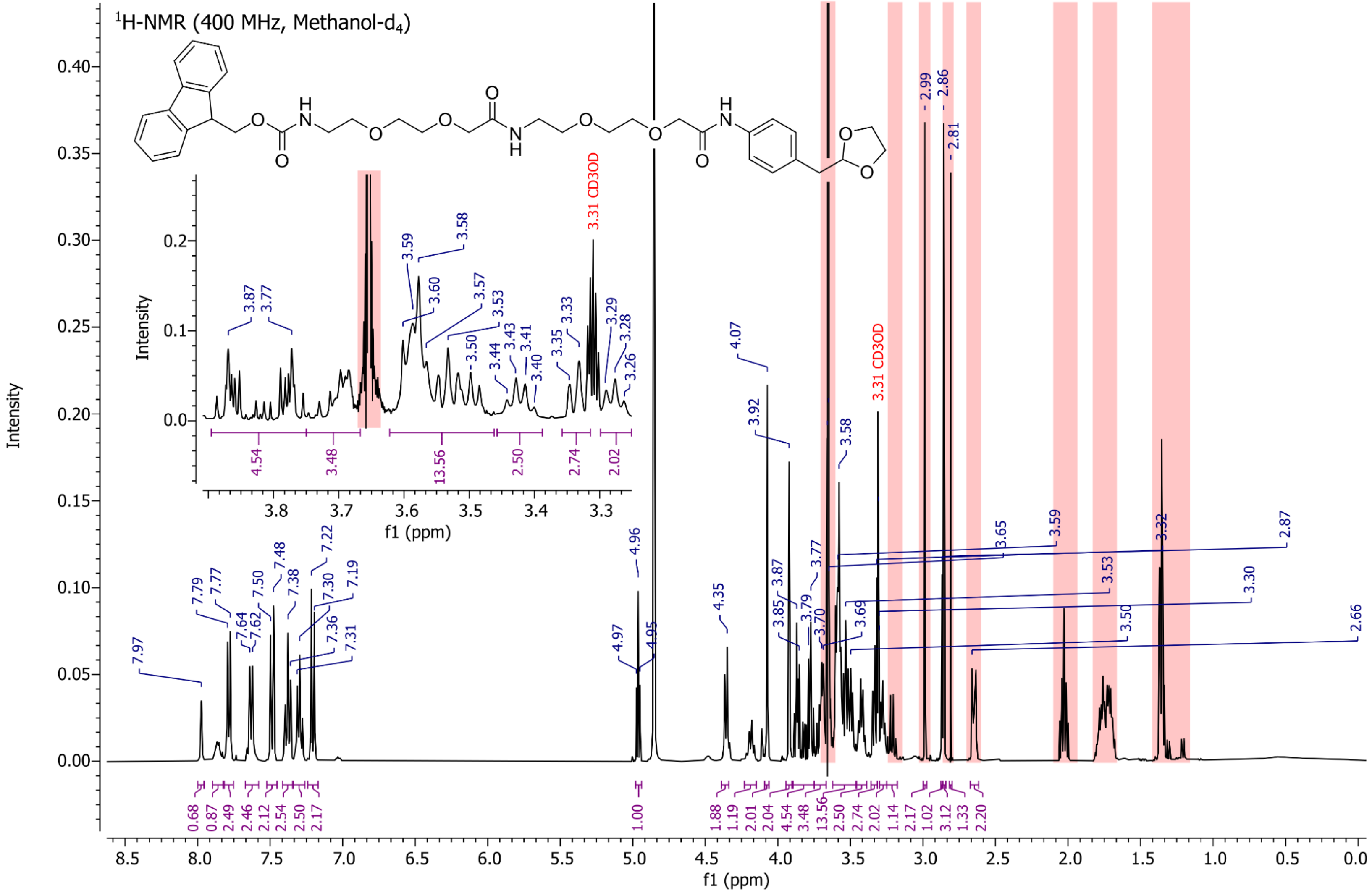

Figure S9. Hydrogen NMR of **S2**

Mass Spectrum for (9H-fluoren-9-yl)methyl (17-((4-((1,3-dioxolan-2-yl)methyl)phenyl)amino)-8,17-dioxo-3,6,12,15-tetraoxa-9-azaheptadecyl)carbamate **S2**

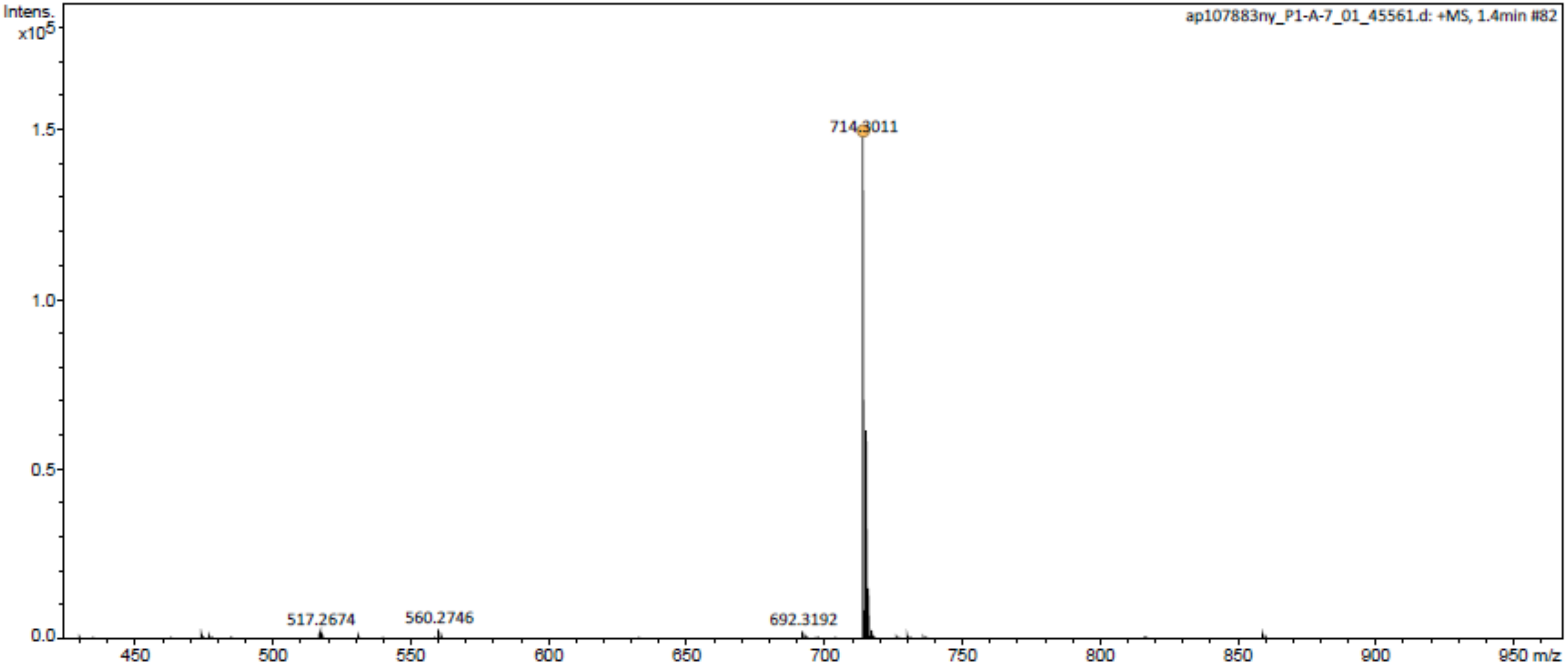

Meas. m/z	#	Ion Formula	m/z	err [ppm]	err [mDa]	mSigma	Mean err [ppm]
714.3011	1	C37H45N3NaO10	714.2997	-1.9	-1.4	3.2	-1.1

Figure S10. Mass spectrum of **S2**

Step 3. Synthesis of N-(4-((1,3-dioxolan-2-yl)methyl)phenyl)-2-((1-((3',6'-dihydroxy-3-oxo-3H-spiro[isobenzofuran-1,9'-xanthen]-5-yl)amino)-10-oxo-1-thioxo-5,8,14-trioxa-2,11-diazahexadecan-16-yl)oxy)acetamide S3

The semi-crude sample of **S2** was dissolved in MeCN (1 mL) inside a 15 mL Falcon™ tube. DBU (100 µL, 102 mg, 0.54 mmol) was then added, and the resultant solution was agitated at rt for 5 minutes. Diethyl ether (17 mL) was then added, causing the solution to become milky due to the precipitation of an oil. Centrifugation was used to pellet the oil and the supernatant was discarded. The pelleted oil was rinsed with diethyl ether (4 mL) and was then dried *in vacuo*. The oil was then dissolved in DMF (3 mL), whereupon triethylamine (140 µL, 101 mg, 1.00 mmol) and fluorescein 5-isothiocyanate (0.195 g, 0.50 mmol) were then added. The resultant solution was then agitated for 8 h at rt in darkness. After this time the solution was concentrated *in vacuo*, and re-dissolved in DCM + 10% acetic acid. The resultant solution was then purified via flash column chromatography using a RediSep Gold® Silica Gel Disposable Flash Column (DCM → 30 % MeOH) to yield **S3** as an orange powder (0.250 g, 62% across two steps).

^{1}H-NMR (400 MHz, Methanol-d$_4$) δ_H 8.16 (d, *J* = 1.81 Hz, 1H), 7.75 (dd, *J* = 8.15, 1.00 Hz, 1H), 7.51-7.44 (m, 2H), 7.24-7.17 (m, 2H), 7.11 (d, *J* = 8.15, 1H), 6.69 (d, *J* = 8.75 Hz, 2H), 6.67 (d, *J* = 2.38 Hz, 2H), 6.54 (dd, *J* = 8.75, 2.38 Hz, 2H), 4.97 (t, *J* = 4.70 Hz, 1H), 4.11 (s, 2H), 3.96 (s, 2H), 3.92-3.77 (m, 6H), 3.77-3.72 (m, 2H), 3.72-3.64 (m, 8H), 3.62 (t, *J* = 5.38 Hz, 2H), 3.45 (t, *J* = 5.42 Hz, 2H), 2.86 (d, *J* = 4.70 Hz, 2H).

^{13}C-NMR (101 MHz, Methanol-d$_4$): δ_c 182.9, 172.9, 171.2, 170.8, 161.9, 154.3, 144.2, 142.5 137.2, 134.0, 131.3, 130.4, 125.8, 125.8, 121.5, 119.8, 113.9, 111.6, 105.7, 103.5, 72.0, 72.0, 71.5, 71.2, 71.2, 71.0, 70.5, 65.9, 45.4, 41.0, 39.6.

FT-IR (ATR) (υmax/cm^{-1}): 3288 (O-H stretch), 2879 (C-H stretch), 1741 (C=O stretch), 1658 (C=O stretch), 1607, 1108 (C-O stretch, ether).

(ESI)HRMS: (Negative mode) Found 857.2705, $C_{43}H_{45}N_3O_{13}S^-$ requires 857.2709.

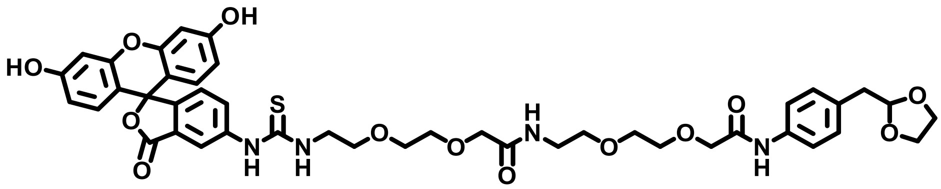

Figure S11. Structure of N-(4-((1,3-dioxolan-2-yl)methyl)phenyl)-2-((1-((3',6'-dihydroxy-3-oxo-3H-spiro[isobenzofuran-1,9'-xanthen]-5-yl)amino)-10-oxo-1-thioxo-5,8,14-trioxa-2,11-diazahexadecan-16-yl)oxy)acetamide **S3**

Hydrogen NMR Spectrum for N-(4-((1,3-dioxolan-2-yl)methyl)phenyl)-2-((1-((3',6'-dihydroxy-3-oxo-3H-spiro[isobenzofuran-1,9'-xanthen]-5-yl)amino)-10-oxo-1-thioxo-5,8,14-trioxa-2,11-diazahexadecan-16-yl)oxy)acetamide **S3**

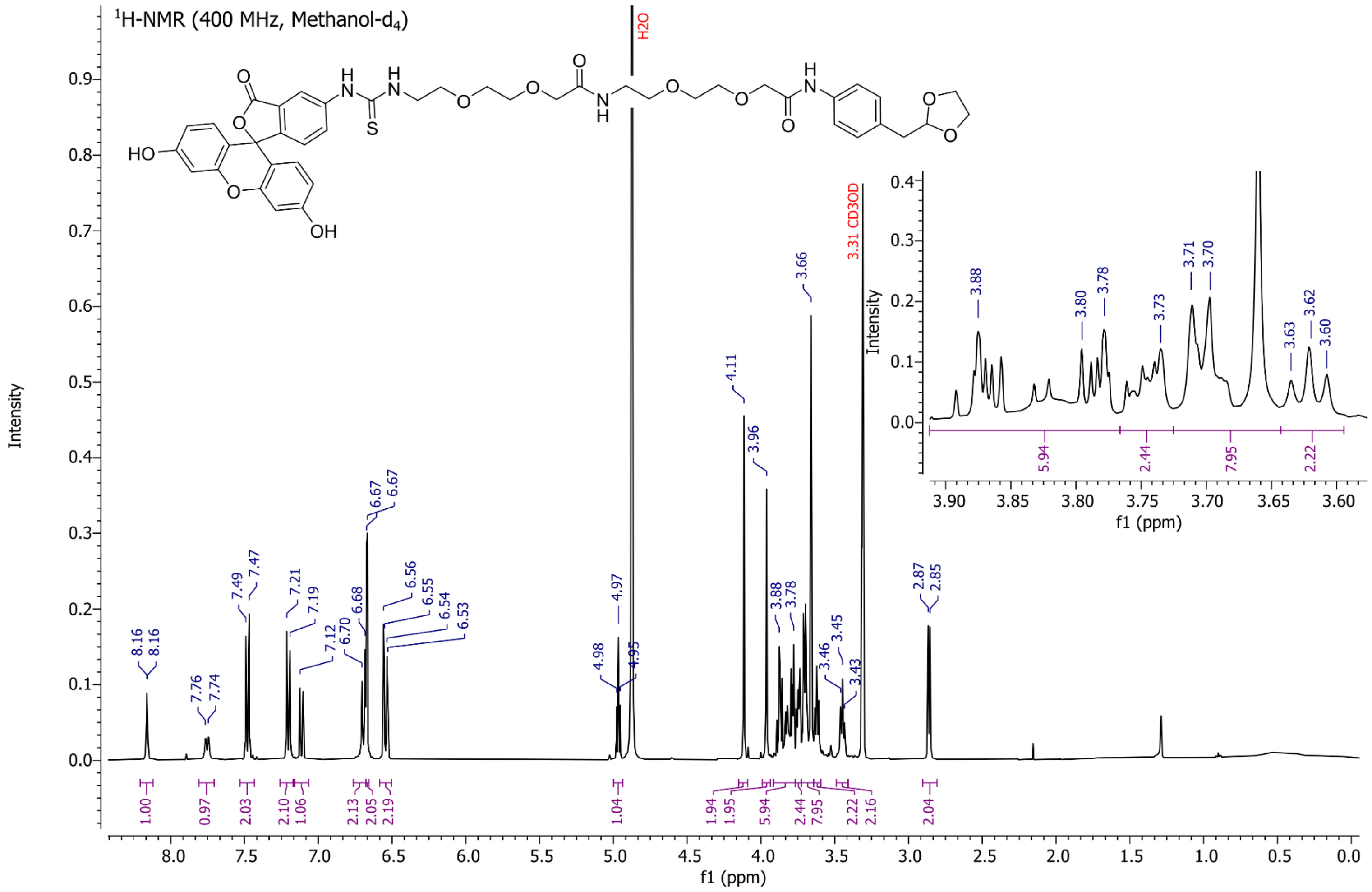

Figure S12. Hydrogen NMR spectrum of **S3**

Carbon NMR Spectrum for N-(4-((1,3-dioxolan-2-yl)methyl)phenyl)-2-((1-((3',6'-dihydroxy-3-oxo-3H-spiro[isobenzofuran-1,9'-xanthen]-5-yl)amino)-10-oxo-1-thioxo-5,8,14-trioxa-2,11-diazahexadecan-16-yl)oxy)acetamide **S3**

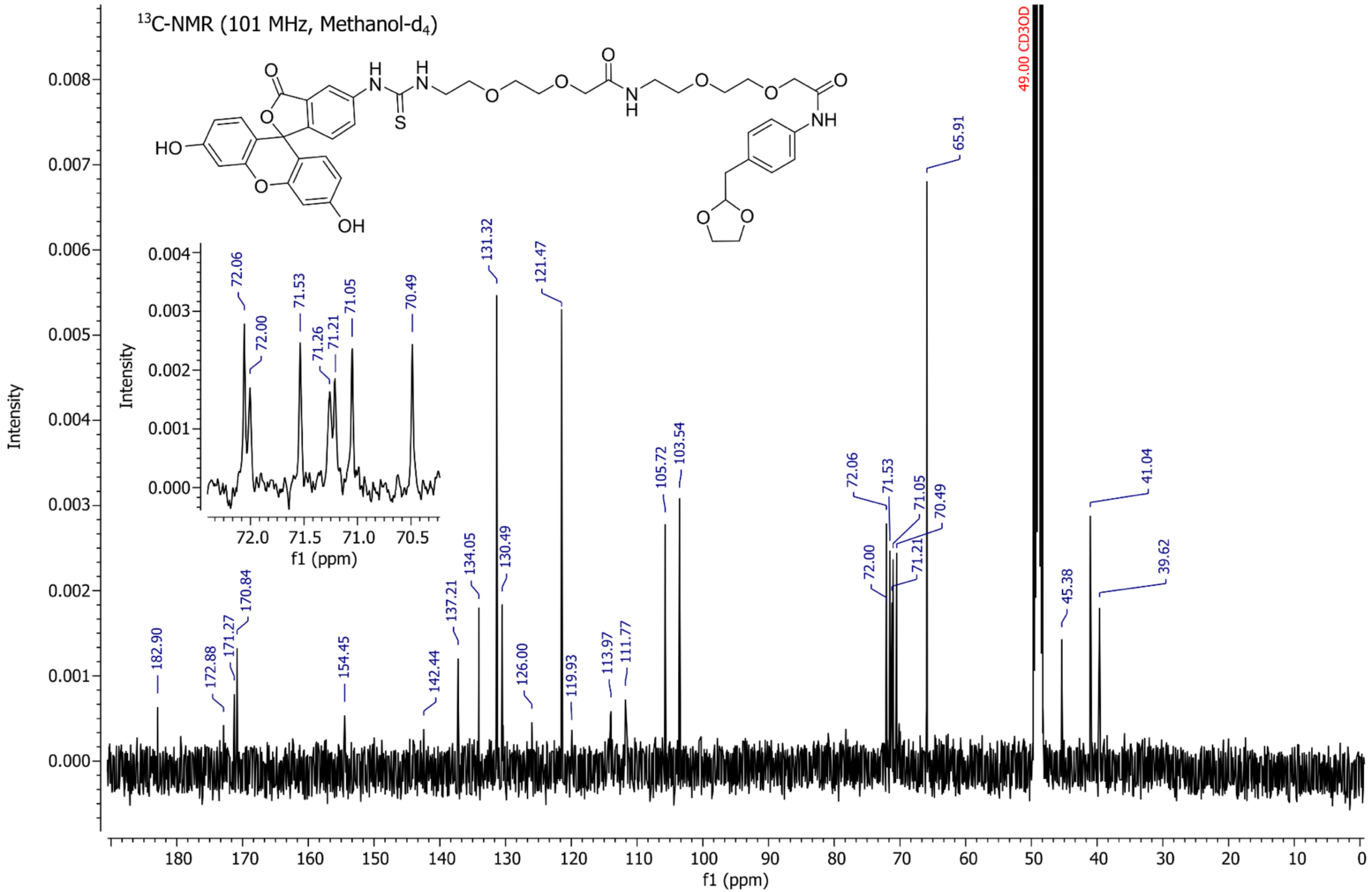

Figure S13. Carbon NMR of **S3**

Mass Spectrum for N-(4-((1,3-dioxolan-2-yl)methyl)phenyl)-2-((1-((3',6'-dihydroxy-3-oxo-3H-spiro[isobenzofuran-1,9'-xanthen]-5-yl)amino)-10-oxo-1-thioxo-5,8,14-trioxa-2,11-diazahexadecan-16-yl)oxy)acetamide **S3**

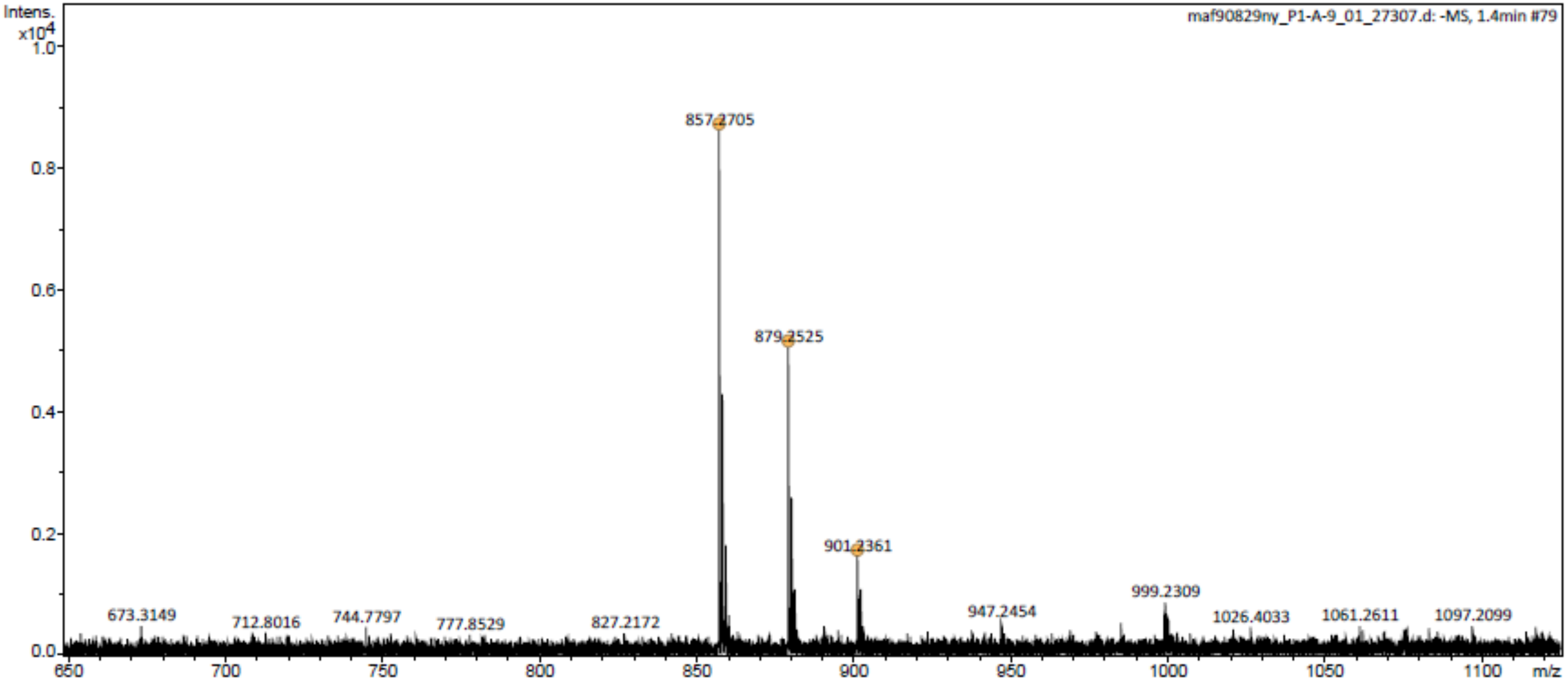

Meas. m/z	#	Ion Formula	m/z	err [ppm]	err [mDa]	mSigma	Mean err [ppm]
857.2705	1	C43H45N4O13S	857.2709	0.6	0.5	16.3	-2.9
879.2525	1	C43H44N4NaO13S	879.2529	0.4	0.4	20.3	3.4
901.2361	1	C43H43N4Na2O13S	901.2348	-1.5	-1.3	94.4	1.5

Figure S14. Mass spectrum of **S3**

FT-IR (ATR) Spectrum for N-(4-((1,3-dioxolan-2-yl)methyl)phenyl)-2-((1-((3',6'-dihydroxy-3-oxo-3H-spiro[isobenzofuran-1,9'-xanthen]-5-yl)amino)-10-oxo-1-thioxo-5,8,14-trioxa-2,11-diazahexadecan-16-yl)oxy)acetamide **S3**

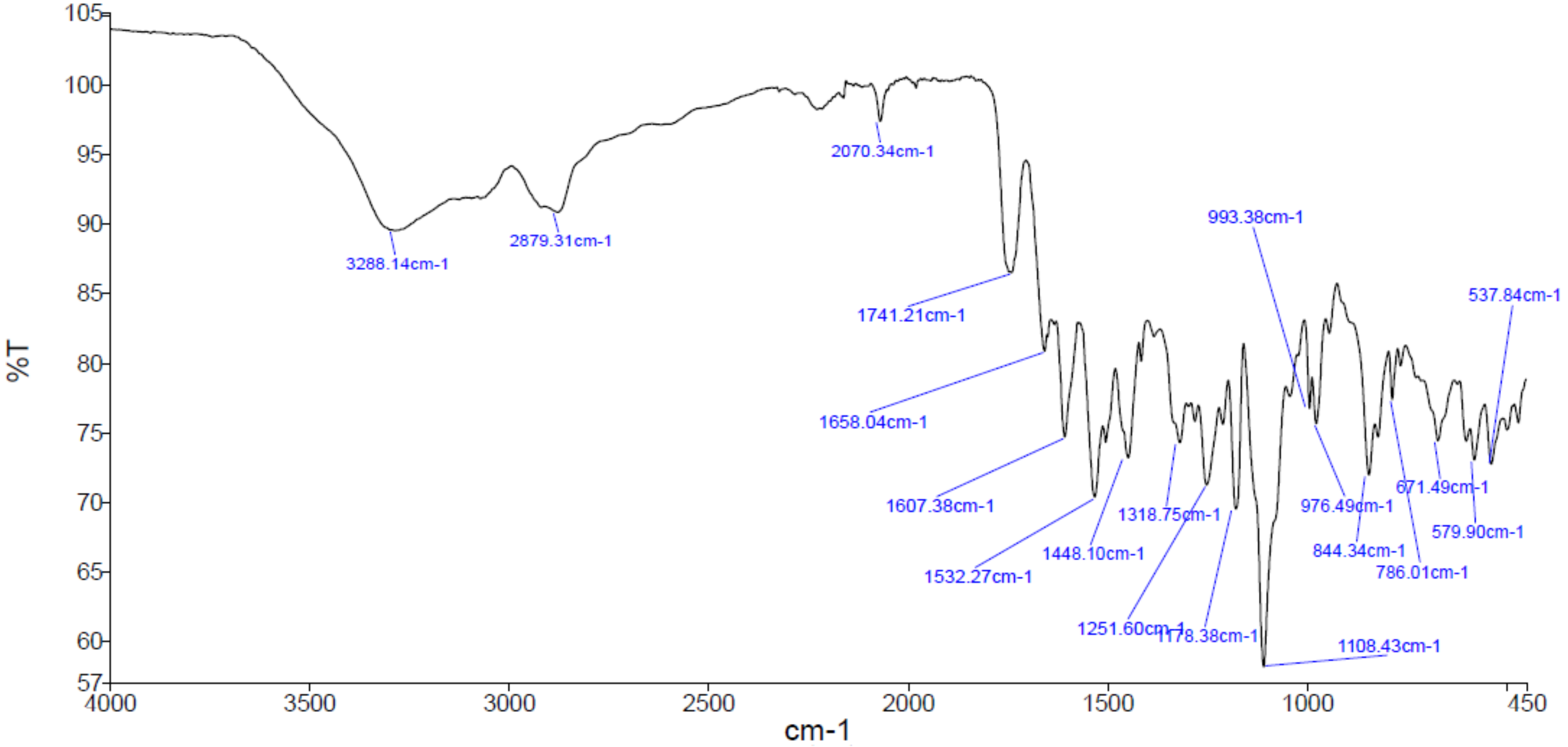

Figure S15. IR spectrum **S3**

Step 4 Synthesis of 2-((1-((3',6'-dihydroxy-3-oxo-3H-spiro[isobenzofuran-1,9'-xanthen]-5-yl)amino)-10-oxo-1-thioxo-5,8,14-trioxa-2,11-diazahexadecan-16-yl)oxy)-N-(4-(2-oxoethyl)phenyl)acetamide S4

A solution of **S3** (10 mg) in 1:1 MeCN:1M HCl (2 mL) was stirred at RT overnight in darkness. A small amount of NaCl (solid) was added to the solution and the solution was shaken to induce phase separation. The organic layer was extracted and diluted with additional MeCN and minimal methanol to afford a yellow solution. The solution was dried ($MgSO_4$) and concentrated using an N_2 stream affording a crude yellow solid. The solid was semi-purified using column chromatography eluting with a solvent system of 10% MeOH in DCM yielding a crude solid of **S4** (3 mg).

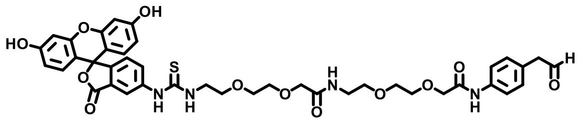

Figure S16. Structure 2-((1-((3',6'-dihydroxy-3-oxo-3H-spiro[isobenzofuran-1,9'-xanthen]-5-yl)amino)-10-oxo-1-thioxo-5,8,14-trioxa-2,11-diazahexadecan-16-yl)oxy)-*N*-(4-(2-oxoethyl)phenyl)acetamide **S4**

Synthesis of a fluorescein-linked colicin E9 conjugate

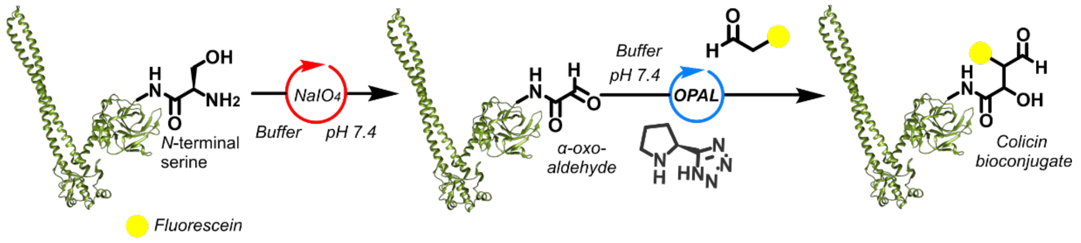

Scheme S3. Depicts initial oxidation of the N-terminus of colicin E9 (PDB; 5EW5[1]) followed OPAL conjugation to a fluorescein linked OPAL probe

A solution of colicin E9 (200 µL of 135 µM stock in 25 mM PB pH 7.5) was charged with L-methionine (3 µL of 66 mM stock in 0.1 M PB, 0.1 NaCl, pH 7.0) and $NaIO_4$ (3 µL of 33 mM stock in 0.1 M PB, 0.1 NaCl, pH 7.0). The solution was mixed by gentle pipette tip swirling and allowed to sit on ice in the dark for 3 minutes. The reaction mixture was immediately purified using a PD SpinTrap G25 desalting column (GE Healthcare Life Sciences), eluting into 200 µL of 25 mM PB pH 7.5. The reaction was charged with (S)-(-)-5-(2-pyrrolidinyl)-H-tetrazole (62 µL of 200 mM stock in 25 mM PB pH 7.5) and fluorescein OPAL probe **S4** (125 µL of 4 mM stock in 25 mM PB pH 7.5). The solution was mixed via pipette tip swirling and incubated for 1 h at 37 °C. The reaction mixture was purified using a PD SpinTrap G25 desalting column (GE Healthcare Life Sciences), eluting into 20 mM K phosphate, 500 mM NaCl pH 7.0 for analysis and further manipulation.

Raw SDS-PAGE gel and Western blot

Coomassie Stained SDS-PAGE gel

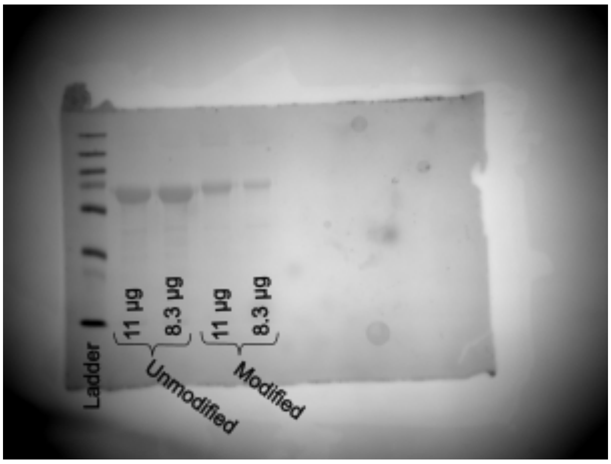

Figure S17. Coomassie stained SDS PAGE analysis of the fluorescein-linked colicin E9 conjugate

Fluorescent imaged SDS-PAGE gel

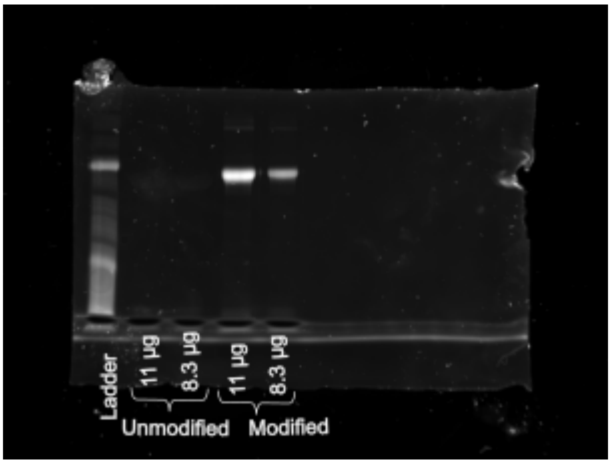

Figure S18. Fluorescent imaged SDS PAGE analysis of the fluorescein-linked colicin E9 conjugate

Binding microscopy using of a fluorescein labelled colicin conjugate

Methods

Col-E9-fluorescein screening

E. coli K12 substr. BW25113 and Δ*btuB* cells cultured in supplemented M9 minimal liquid medium, pH 7.2 to an OD_{600nm} of approximately 0.5 (mid exponential phase). Cells were then harvested via centrifugation (8000 x *g*, 3 min, 4 °C). Cell pellets were resuspended in freshly supplemented M9 to an OD_{600nm} of 1.0 and transferred to 2 mL microcentrifuge tubes. ColE9-FITC was then added to re-suspended samples to a final concentration of 500 nM. Suspensions were subsequently incubated on a slow-moving rotary wheel (30 min, 12 rpm, 80° incline relative to bench top, room temperature). Labelling suspensions were transferred to fresh 1.5 mL microcentrifuge tubes and pelleted via centrifugation. Cell pellets resuspended in M9 minimal medium, transferred to fresh tubes and pelleted via centrifugation. This washing step was repeated three times in total for each sample. Washed, labelled cell pellets resuspended to an OD_{600nm} of 2.0 in freshly supplemented, pre-chilled M9 media. 24 µL of samples were transferred to 500 µL microcentrifuge tubes. 0.5% (w/v) 5 µm silica bead slurry in unsupplemented M9 media added. Suspensions mixed via repeated pipetting. 10 µL pipetted onto centre of 1.0 – 1.2 mm clean glass slide and covered with 18 mm^2 number 1.5 high precision glass coverslip (Zeiss). Slides sealed with nail varnish bead and left to set for approximately to 10 min before imaging.

3D structured illumination microscopy (SIM) imaging was carried out an Zeiss Elyra 7 SRM microscope in lattice SIM mode equipped with Plan-Apochromat 63 x / 1.46 Na Korr oil immersion objective Var 2 lens and 488 nm solid state laser (2.0 – 2.5 %). Z-stack images collected for 3D image reconstruction (0.101 µm optical sectioning), 50 ms exposure time. SIM^2 image processing carried out in ZEN Black 3.0 SR software. 3D depth coded images produced in ZEN Blue software. Confocal fluorescence microscopy imaging was carried out using a Zeiss 910 LSM upright confocal microscope equipped with 63 x / 1. 46 Na oil immersion objective lens and 488 nm laser (power maintained at 1.0%). Pinhole set to 1 airy unit, electronic gain was set and maintained at 780. Images were saved in 16-Bit format and processed using FIJI / ImageJ.

Formation of a Mannose-linked colicin E9 conjugate

Synthesis of Mannose azide 4

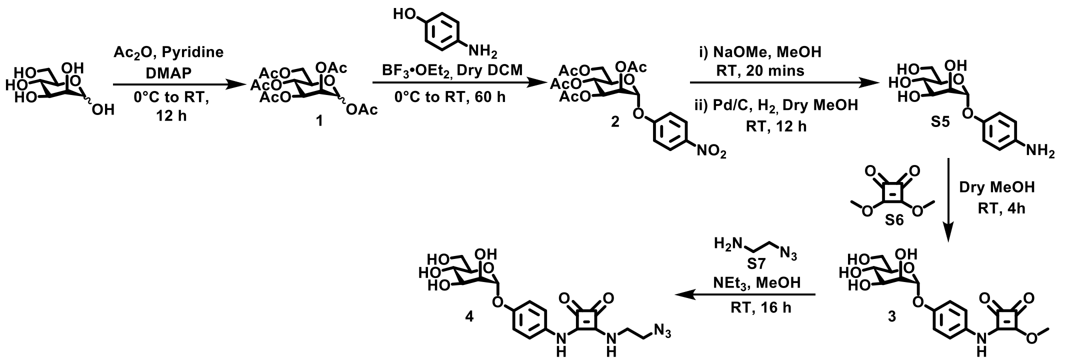

Scheme S3. Synthesis pathway for mannose azide **4**

Step 1. Synthesis of D-mannose pentaacetate 1[4]

Acetic anhydride (42 mL, 410 mmols) was added to a solution of D-mannose (20 g, 100 mmols) and DMAP (cat) in pyridine (42 mL) at 0 °C affording a cloudy white solution. The reaction mixture was warmed to RT and stirred for a further 12 h, affording a clear yellow solution. The solution was concentrated *in vacuo*, affording a caramel coloured oil, which was diluted with DCM (40 mL), washed with water (40 mL), 1 M HCl acid solution (40 mL) and saturated $NaHCO_3$ solution (40 mL). The solution was dried ($MgSO_4$) and concentrated *in vacuo* to afford a caramel oil of **1** which was used crude in following reactions (35 g, 89 mmols, 80%); R_f = 0.5 (2:1 Hexane : EtOAc).

^{1}H NMR (400 MHz, $CDCl_3$) δ 6.08 (d, $J_{1,2}$ = 2.0 Hz, 1H, H-1), 5.34 (m, 2H, H-3, H-4), 5.25 (dd, $J_{2,3}$ = 2.5 Hz, $J_{1,2}$ = 2.0 Hz, 1H, H-2), 4.28 (dd, $J_{6a,6b}$ = 12.4 Hz, $J_{5,6a}$ = 4.8 Hz, 1H, H-6a), 4.09 (dd, $J_{6a,6b}$ = 12.4 Hz, $J_{5,6b}$ = 2.5 Hz, 1H, H-6b), 4.05 (m, 1H, H-5), 2.17 (s, 3H, CH_3 (OAc)), 2.16 (s, 3H, CH_3 (OAc)), 2.09 (s, 3H, CH_3 (OAc)), 2.05 (s, 3H, CH_3 (OAc)), 2.00 (s, 3H, CH_3 (OAc)).

^{13}C NMR (101 MHz, $CDCl_3$) δ 170.8 (1C), 170.1 (1C), 169.9 (1C), 169.7 (1C), 168.2 (1C), (C=O), 90.8 (1C, C-1), 70.7 (1C, C-5), 68.8 (1C, C-3/C-4), 68.4 (1, C-2), 65.6 (1C, C-3/ C-4), 62.2 (1C, C-6), 21.0 (1C, CH_3), 20.9 (1C, CH_3), 20.9 (1C, CH_3), 20.8 (2C, CH_3).

(ESI)HRMS – $C_{16}H_{22}NaO_{11}^+$ ($[M+Na]^+$) Requires m/z 413.1060: Found m/z 413.1056

[α]D^{25} = + 29.26 (*c* 1, DCM)

FT-IR (ATR) – 2973 (C-H), 1748 (C=O), 1370 (C-O), 1217 (C-O)

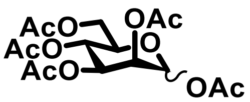

Figure S19. Structure of D-mannose pentaacetate **1**

Hydrogen NMR Spectrum of D-Mannose Pentaacetate **1**

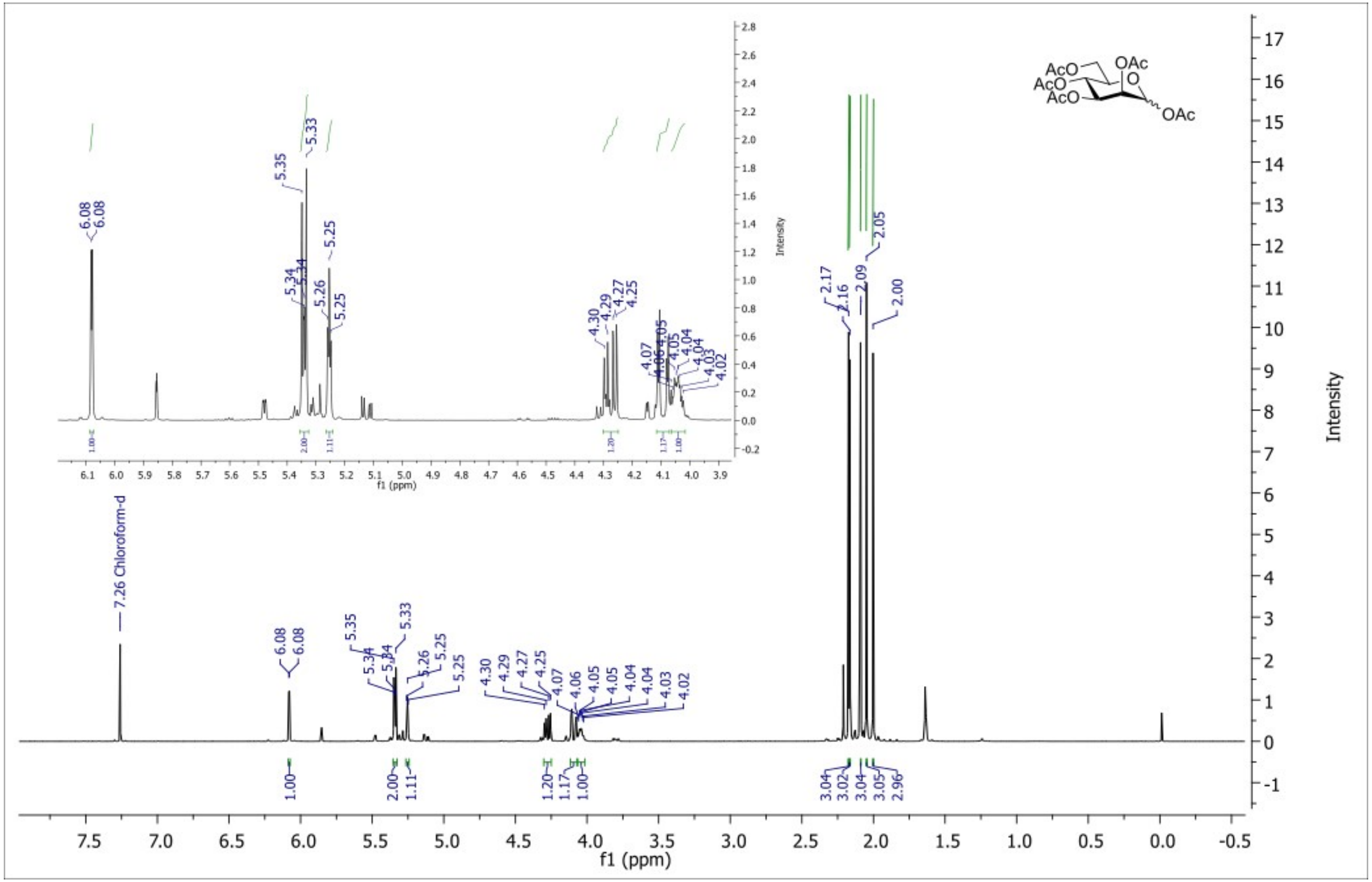

Figure S20. Hydrogen NMR spectrum of **1**

Carbon NMR Spectrum of D-Mannose Pentaacetate **1**

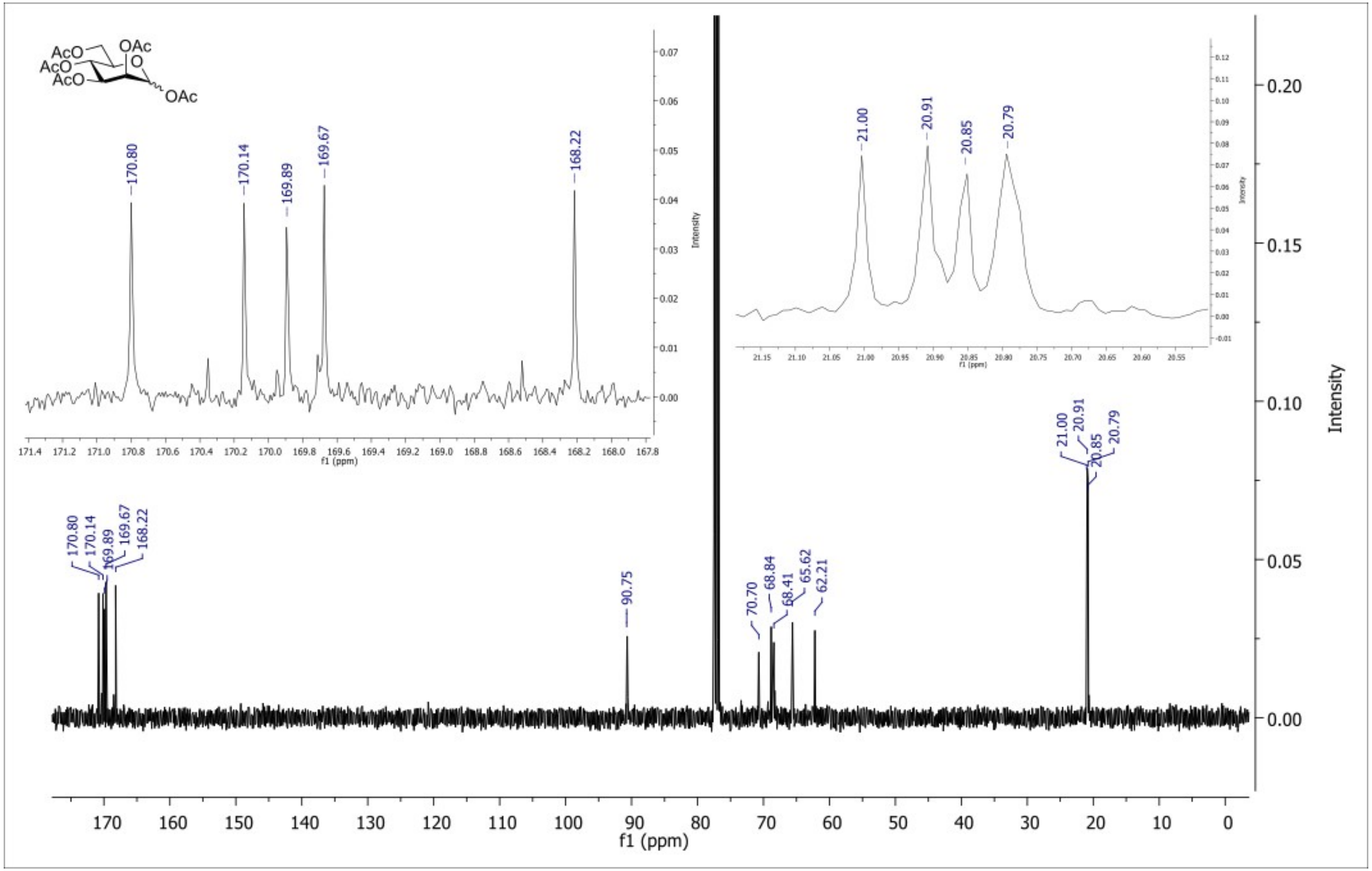

Figure S21. Carbon NMR spectrum of **1**

Mass Spectrum of D-Mannose Pentaacetate **1**

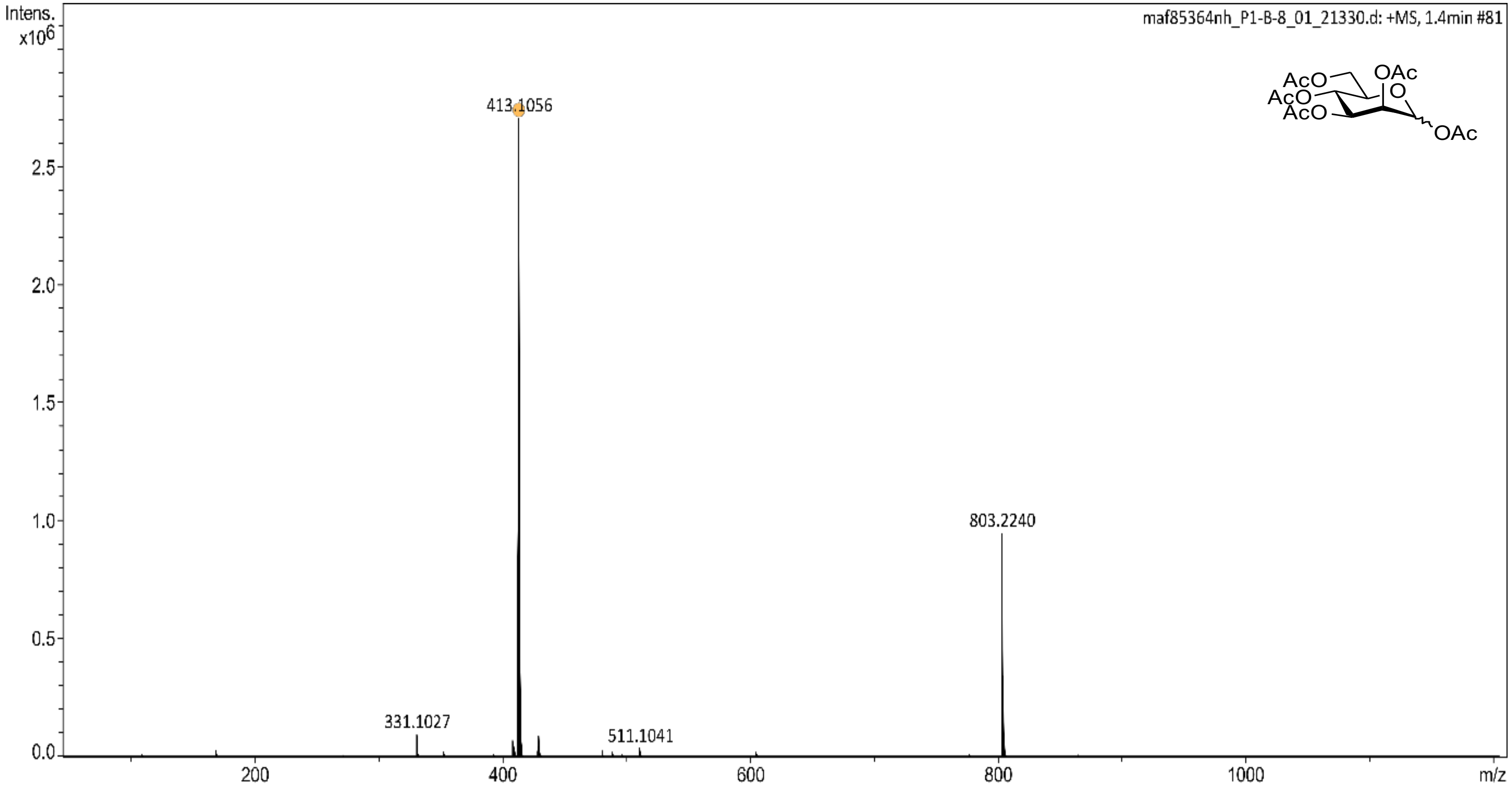

Meas. m/z	#	Ion Formula	m/z	err [ppm]	err [mDa]	mSigma	Mean err [ppm]
413.1056	1	C16H22NaO11	413.1054	-0.5	-0.2	1.7	-0.5

Figure S22. Mass spectrum of **1**

FT-IR (ATR) Spectrum of D-Mannose Pentaacetate **1**

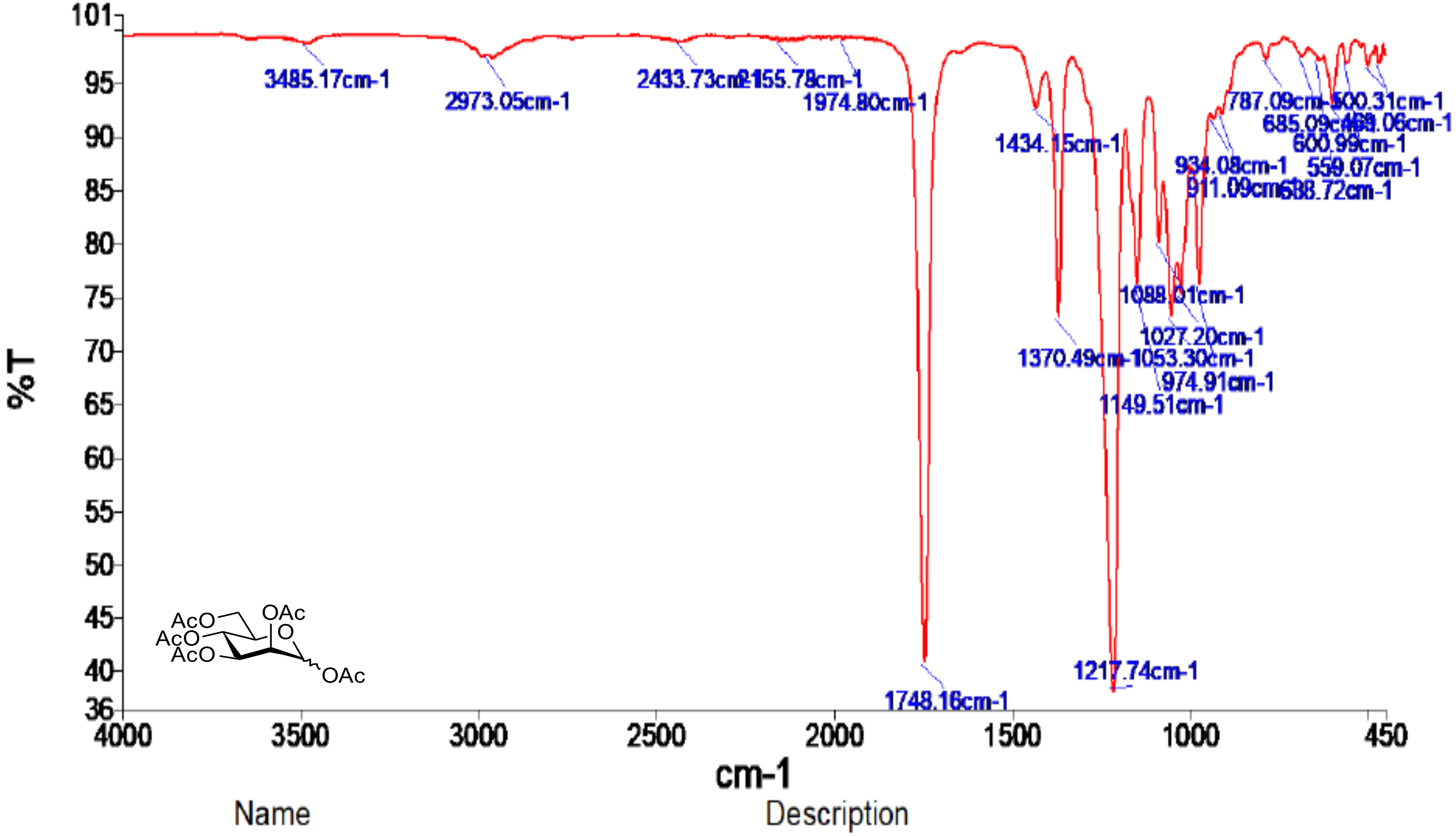

Figure S23. IR spectrum of **1**

Step 2. Synthesis of 4-Nitrophenyl 2,3,4,6-tetra-*O*-acetyl-α-D-mannopyranoside[5] 2

$BF_3 \cdot OEt_2$ (1.5 mL, 12 mmols, 3 eq) was added dropwise to a solution of **1** at 0 °C (1.6 g, 3.9 mmols) and 4-nitrophenol (1.1 g, 7.8 mmols, 2 eq) in dry DCM (10 mL) at RT under N_2. The reaction mixture was stirred for 2.5 days, affording an orange solution. The solution was quenched with saturated $NaHCO_3$ solution (10 mL) and the organic layer extracted, washed with saturated NaCl solution (10 mL), dried ($MgSO_4$) and concentrated *in vacuo* to yield an orange oil. This oil was purified using column chromatography, eluting with a graduated solvent system of 3 : 1 Hexane : EtOAc to 1 : 1 Hex : EtOAc. The semi-crude product was recrystallized from methanol, affording white crystals of **2** (0.8 g, 1.7 mmols, 43%); R_f = 0.58 (3:1 Toluene : EtOAc).

^{1}H NMR (500 MHz, $CDCl_3$) δ 8.21 (d, J_{HAr} = 9.30 Hz, 2H, HAr), 7.20 (d, J_{HAr} = 9.30 Hz, 2H, HAr), 5.62 (d, $J_{1,2}$ = 1.8 Hz, 1H, H-1), 5.52 (dd, $J_{3,4}$ = 10.0 Hz, $J_{2,3}$ = 3.4 Hz, 1H, H-3), 5.44 (dd, $J_{2,3}$ = 3.4 Hz, $J_{1,2}$ = 1.8 Hz, 1H, H-2), 5.37 (dd, $J_{3,4}$ = 10.0 Hz, $J_{4,5}$ = 10.0 Hz, 1H, H-4), 4.25 (dd, $J_{6a,6b}$ = 12.3 Hz, $J_{5,6a}$ = 5.5 Hz, 1H, H-6a), 4.06 (dd, $J_{6a,6b}$ = 12.3 Hz, $J_{5,6b}$ = 2.3 Hz, 1H, H-6b), 4.00 (ddd, $J_{4,5}$ = 10.0 Hz, $J_{5,6a}$ = 5.5 Hz, $J_{5,6b}$ = 2.3 Hz, 1H, H-5), 2.20 (s, 3H, CH_3), 2.04 (s, 3H, CH_3), 2.03 (s, 3H, CH_3), 2.01 (s, 3H, CH_3).

^{13}C NMR (126 MHz, $CDCl_3$) δ 170.5 (1C), 170.0 (1C), 170.0 (1C), 169.9 (1C), (C=O), 160.3, 143.3, 125.9, 116.6, (CAr), 95.8 (1C, C-1), 69.9 (1C, C-5), 69.0 (1C, C-2), 68.6 (1C, C-3), 65.7 (1C, C-4), 62.1 (1C, C-6), 20.9 (1C, CH_3), 20.8 (1C, CH_3), 20.7 (1C, CH_3), 20.7 (1C, CH_3).

(ESI)HRMS – $C_{20}H_{23}O_{12}NNa^+$ ($[M+Na]^+$) requires m/z 492.1118: found m/z 492.1121

[α]D^{25} = + 94.40 (*c* 1, DCM)

FT-IR (ATR) – 3127 (C-H), 2973 (C-H), 1954 (C-H), 1742 (C=O), 1592 (C=C), 1519 (N-O), 1343 (N-O), 1214 (C-O), 1025 (C-O).

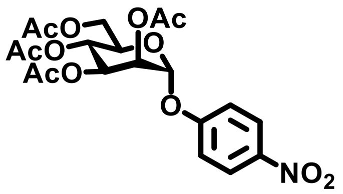

Figure S24. Structure of 4-Nitrophenyl 2,3,4,6-tetra-*O*-acetyl-α-D-mannopyranoside **2**

Hydrogen NMR Spectrum of 4-Nitrophenyl 2,3,4,6-tetra-O-acetyl-α-D-mannopyranoside **2**

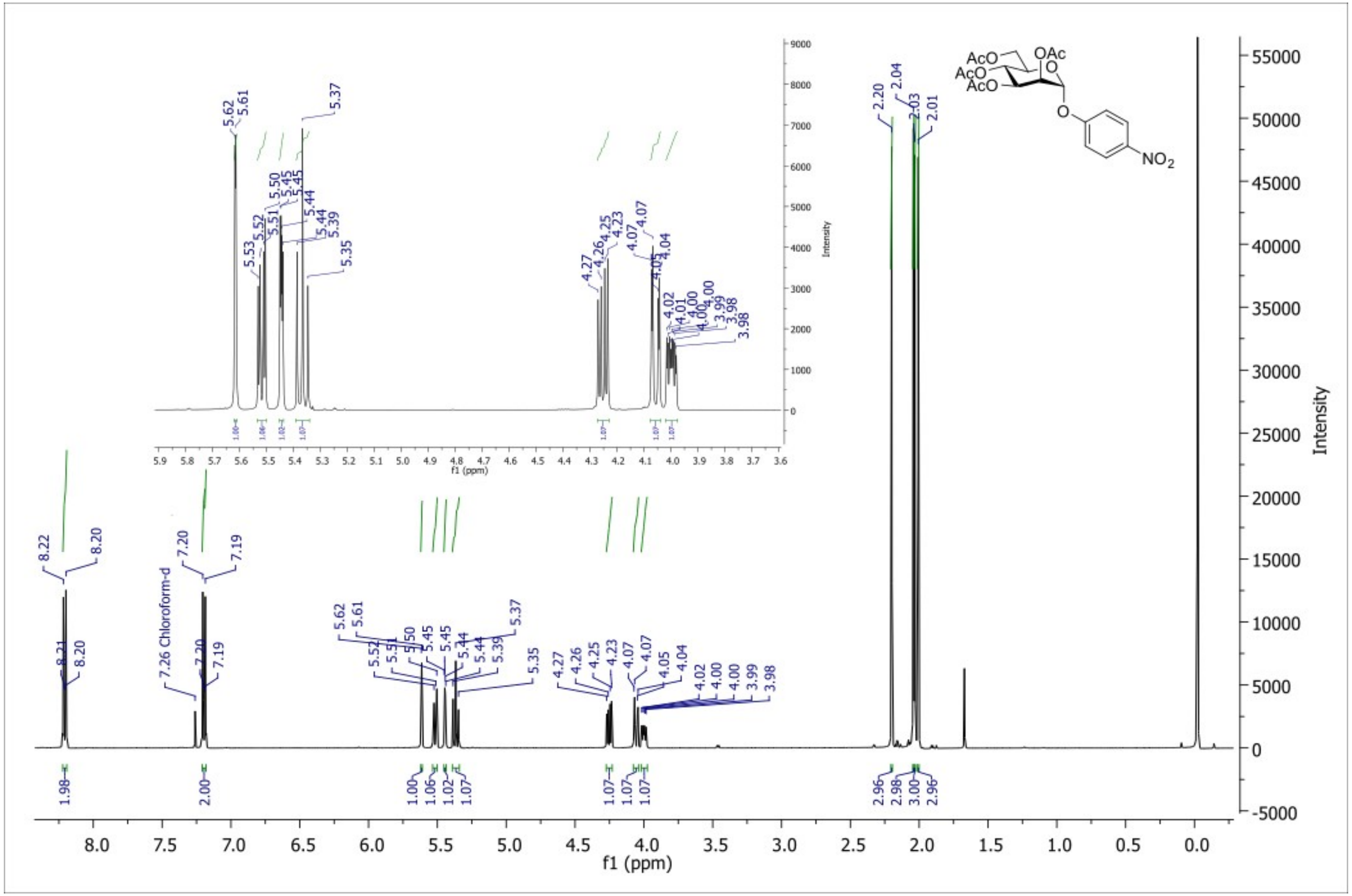

Figure S25. Hydrogen NMR spectrum of **2**

Carbon NMR Spectrum of 4-Nitrophenyl 2,3,4,6-tetra-O-acetyl-α-D-mannopyranoside **2**

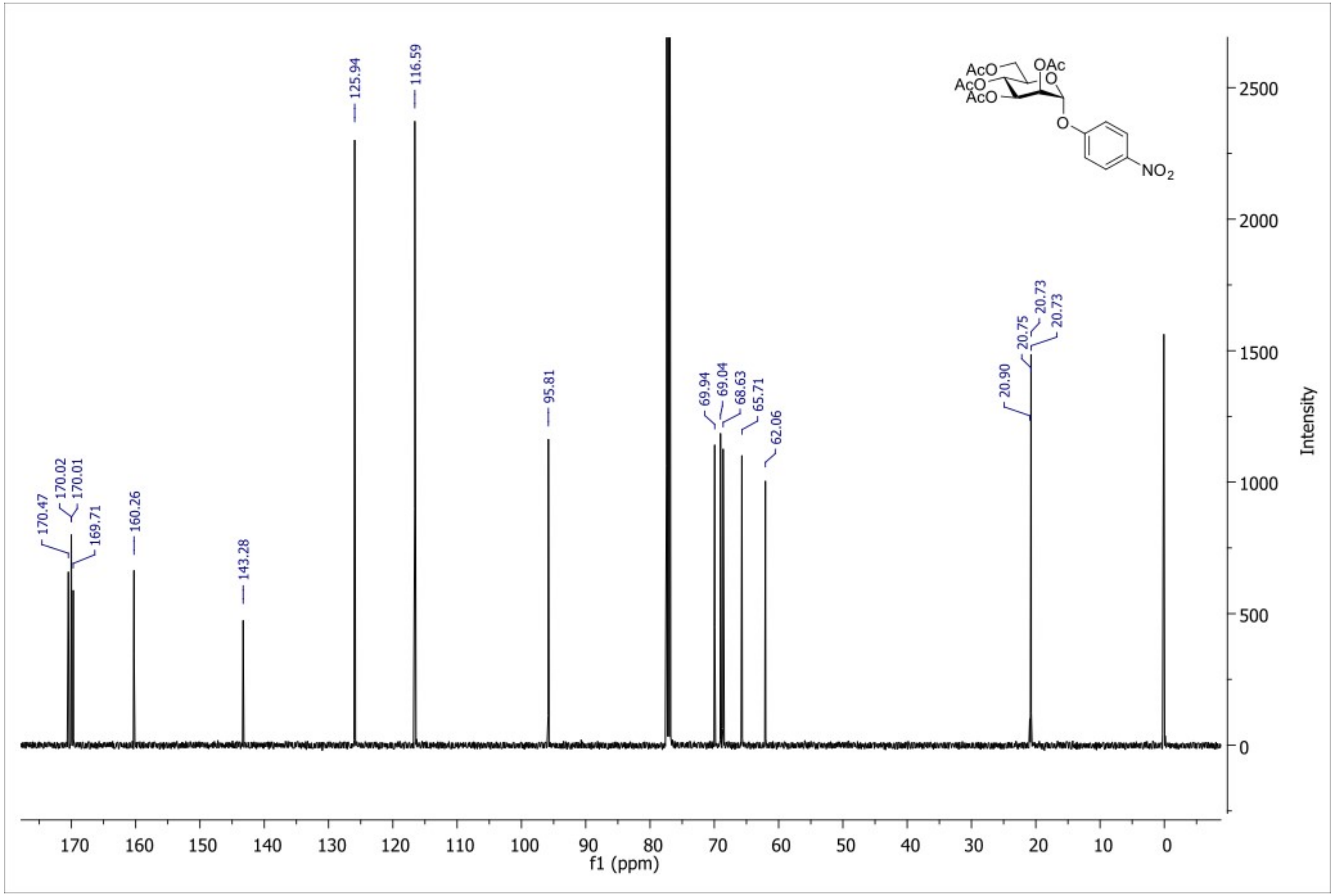

Figure S26. Carbon NMR spectrum of **2**

Mass Spectrum of 4-Nitrophenyl 2,3,4,6-tetra-O-acetyl-α-D-mannopyranoside **2**

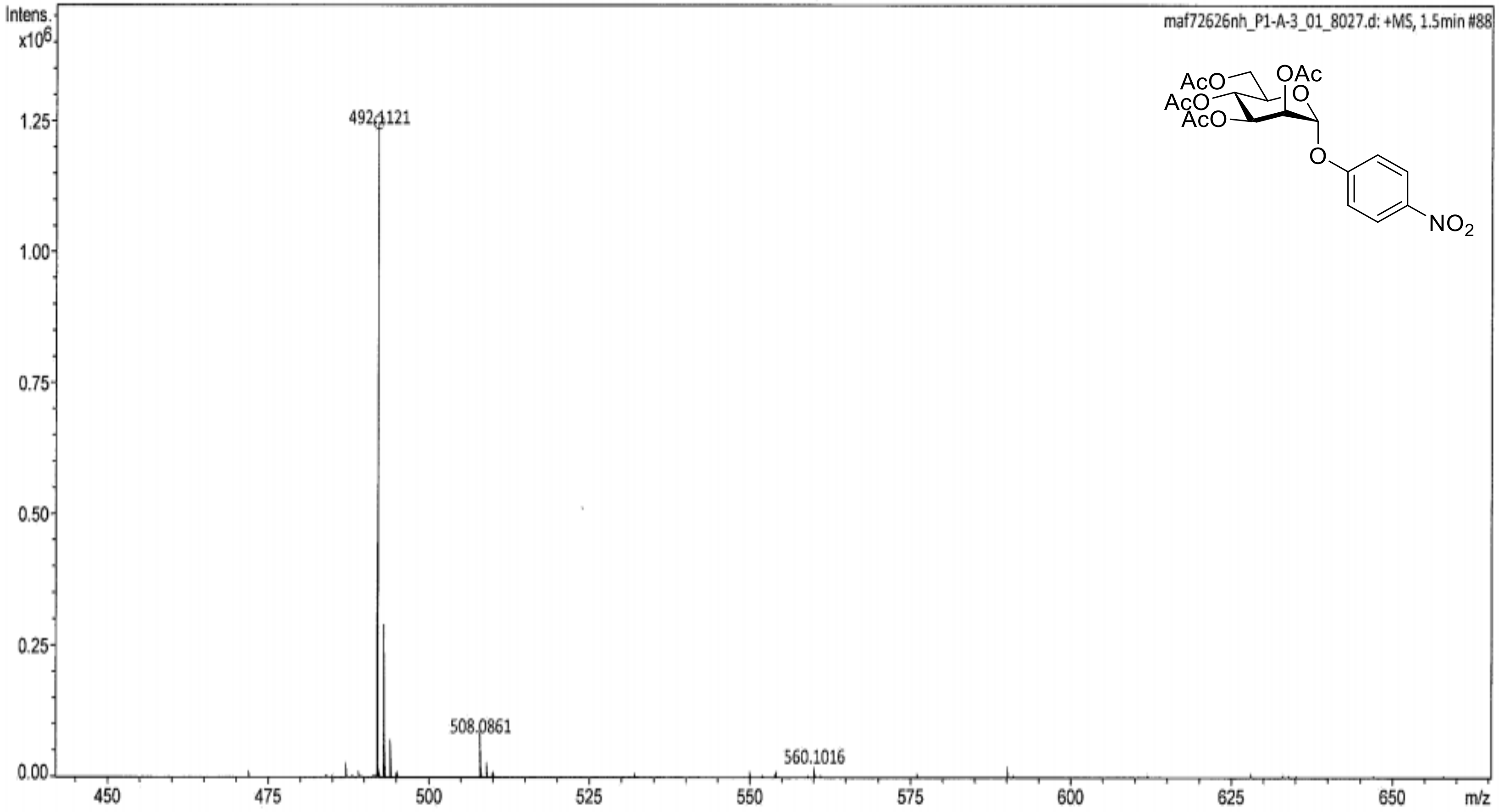

Meas. m/z	#	Ion Formula	m/z	err [ppm]	err [mDa]	mSigma	Mean err [ppm]
492.1121	1	C20H23NNaO12	492.1112	-1.7	-0.9	0.6	-2.1

Figure S27. Mass spectrum of **2**

FT IR (ATR) Spectrum of 4-Nitrophenyl 2,3,4,6-tetra-O-acetyl-α-D-mannopyranoside **2**

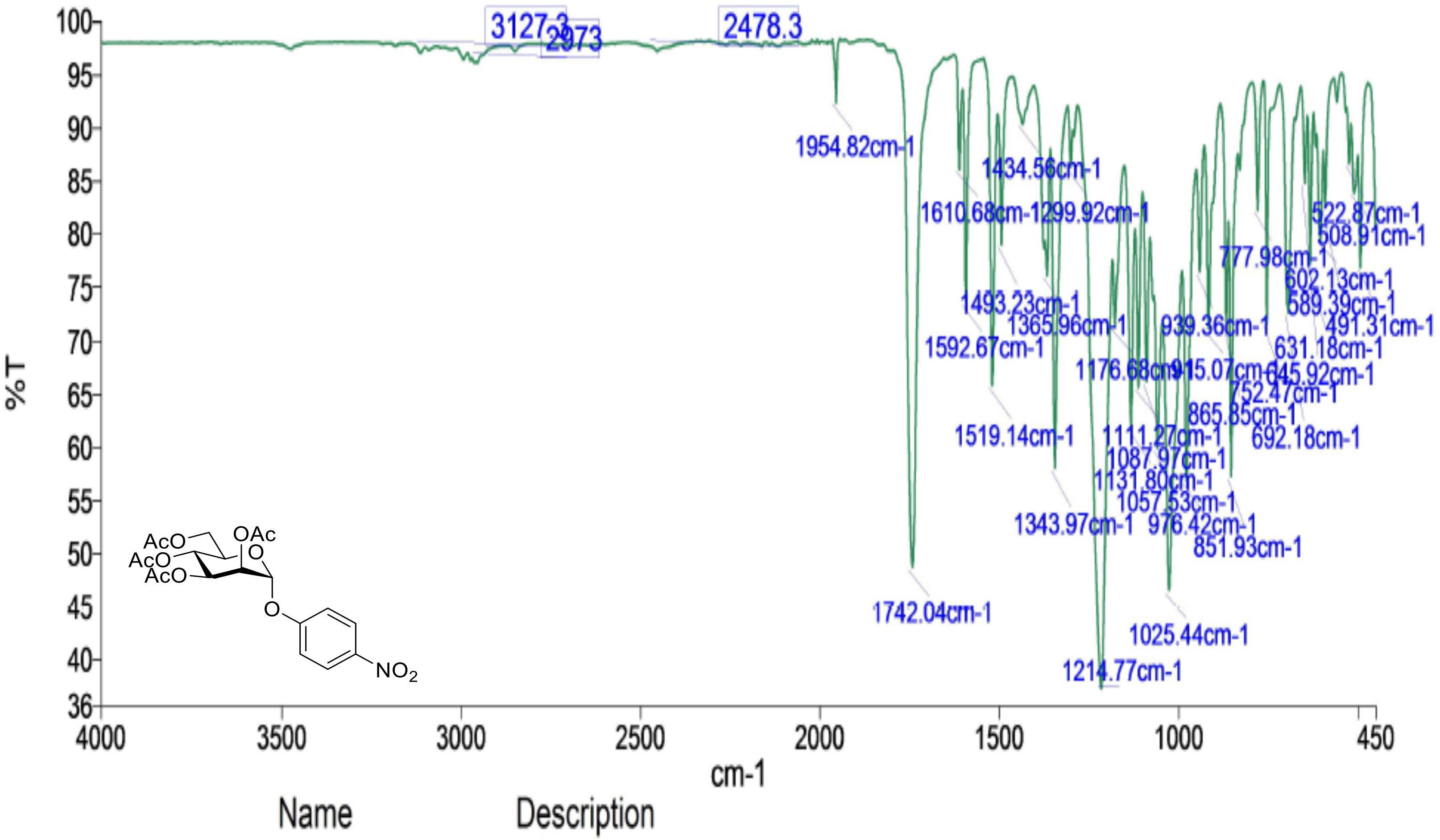

Figure S28. IR spectrum of **2**

Step 3. Synthesis of 4-Aminophenyl α-D-mannopyranoside S5[6]

A 30% NaOMe solution in MeOH was added dropwise to a solution of **2** (0.75 g 1.6 mmols) in MeOH (10 mL) until pH 10 was reached. The reaction mixture was stirred at RT for 20 minutes, affording a clear solution. The solution was neutralised with dowex which was subsequently removed *via* filtration to leave a clear solution. The solution was concentrated *in vacuo***,** affording a white solid which was dried *in vacuo* for 2 h. The solid was then dissolved in dry MeOH (10 mL) and Pd/C (cat) was added to the solution. The vessel was placed under nitrogen, and then degassed before backfilling with H_2. The reaction mixture was stirred at RT for 12 h then filtered through celite and concentrated *in vacuo* to yield a white solid of **S5** (0.24 g, 0.84 mmol, 53%).

^{1}H NMR (500 MHz, MeOD) δ 6.90 (d, J_{HAr} = 9.0 Hz, 2H, HAr), 6.69 (d, J_{HAr} = 9.0 Hz, 2H, HAr), 5.28 (d, $J_{1,2}$ = 1.80 Hz, 1H, H-1), 3.98 (dd, $J_{2,3}$ = 3.3 Hz, $J_{1,2}$ = 1.8 Hz, 1H, H-2), 3.87 (dd, $J_{3,4}$ = 9.2 Hz, $J_{2,3}$ = 3.3 Hz, 1H, H-3), 3.79-3.70 (m, 3H, H-4, H-6a, H-6b), 3.67 (ddd, *J* = 9.6, *J* = 4.7 Hz, *J* = 2.4 Hz, 1H, H-5).

^{13}C NMR (126 MHz, MeOD) δ 151.0, 143.5, 119.3, 117.8 (CAr), 101.3 (1C, C-1), 75.1 (1C, C-5), 72.4 (1C, C-3), 72.2 (1C, C-2), 68.4 (1C, C-4), 62.7 (1C, C-6).

(ESI)HRMS – $C_{12}H_{18}O_6NNa^+$ ($[M+Na]^+$) requires m/z 294.0954: found m/z 294.0940

[α]D^{25} = - 244.75 (*c* 1, MeOH)

FT-IR (ATR) – 3317 (O-H), 2939 (C-H), 2469 (C-H), 1510 (C=C), 1050 (C-O), 1019 (C-O).

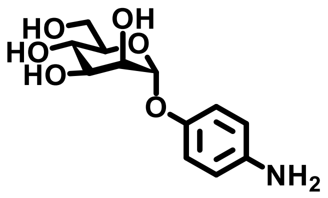

Figure S29. Structure of 4-Aminophenyl α-D-mannopyranoside **S5**

Hydrogen NMR Spectrum of 4-Aminophenyl α-D-mannopyranoside **S5**

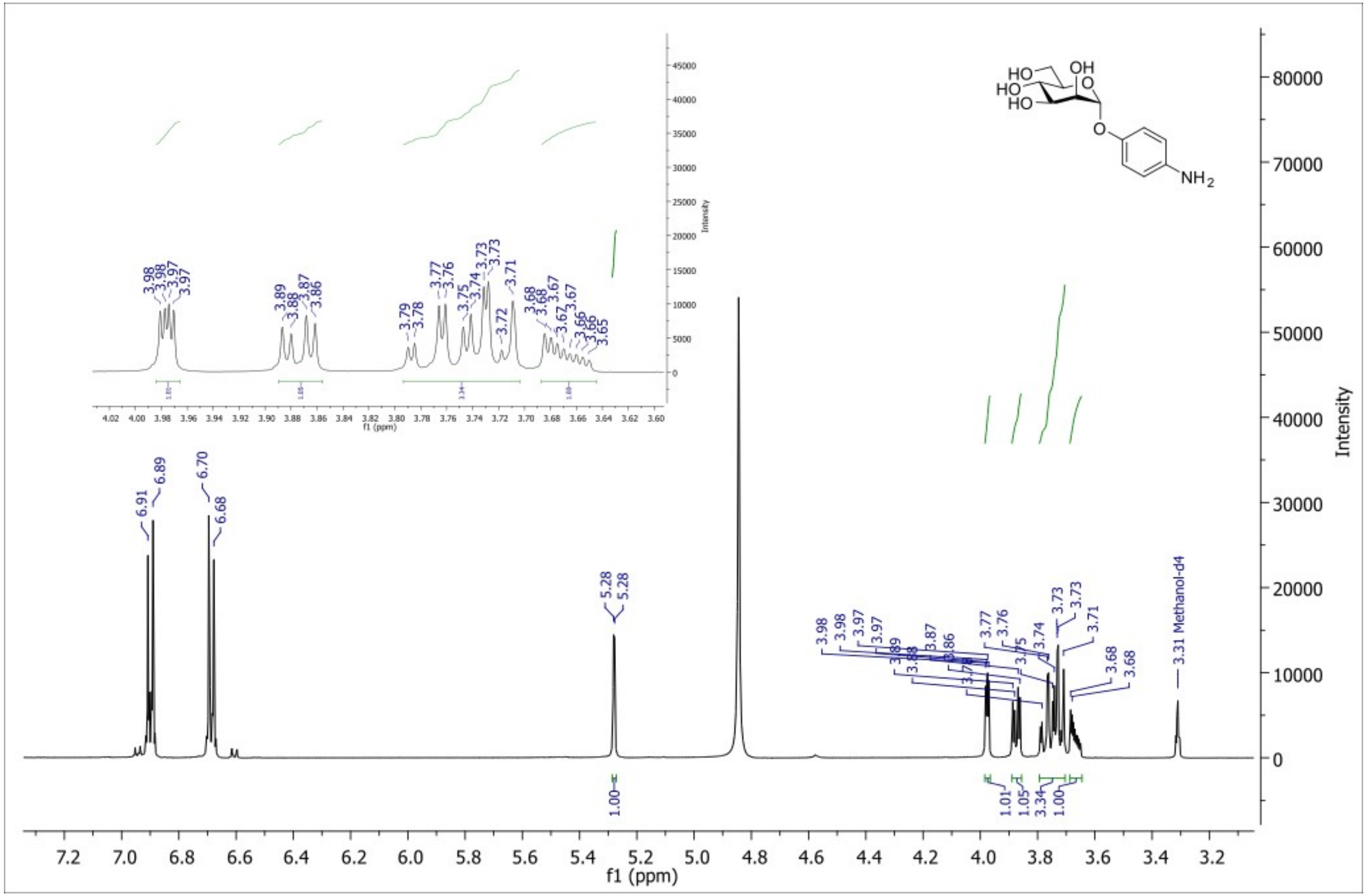

Figure S30. Hydrogen NMR of **S5**

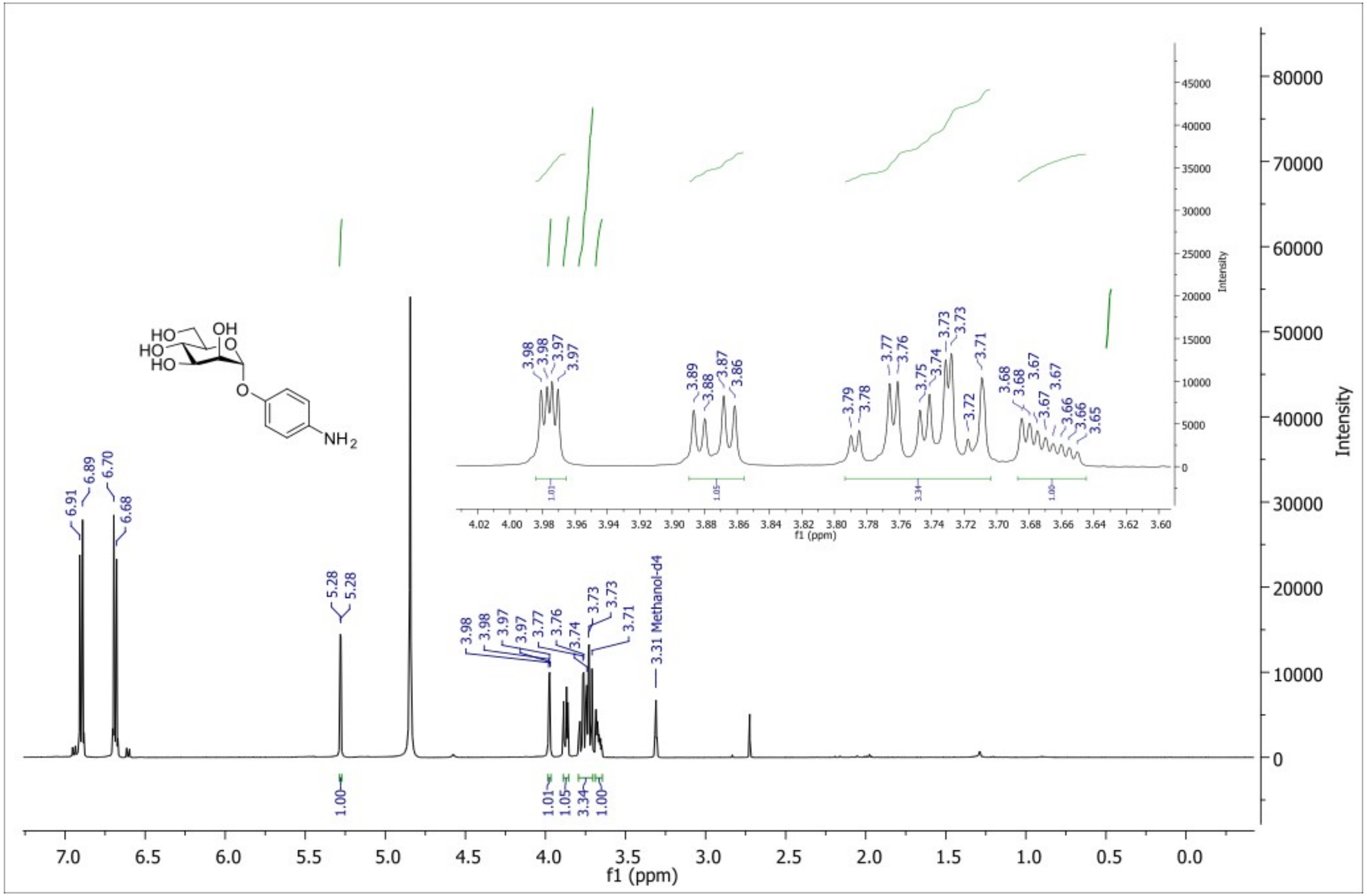

Figure S31. Hydrogen NMR of **S5**

Carbon NMR Spectrum of 4-Aminophenyl α-D-mannopyranoside **S5**

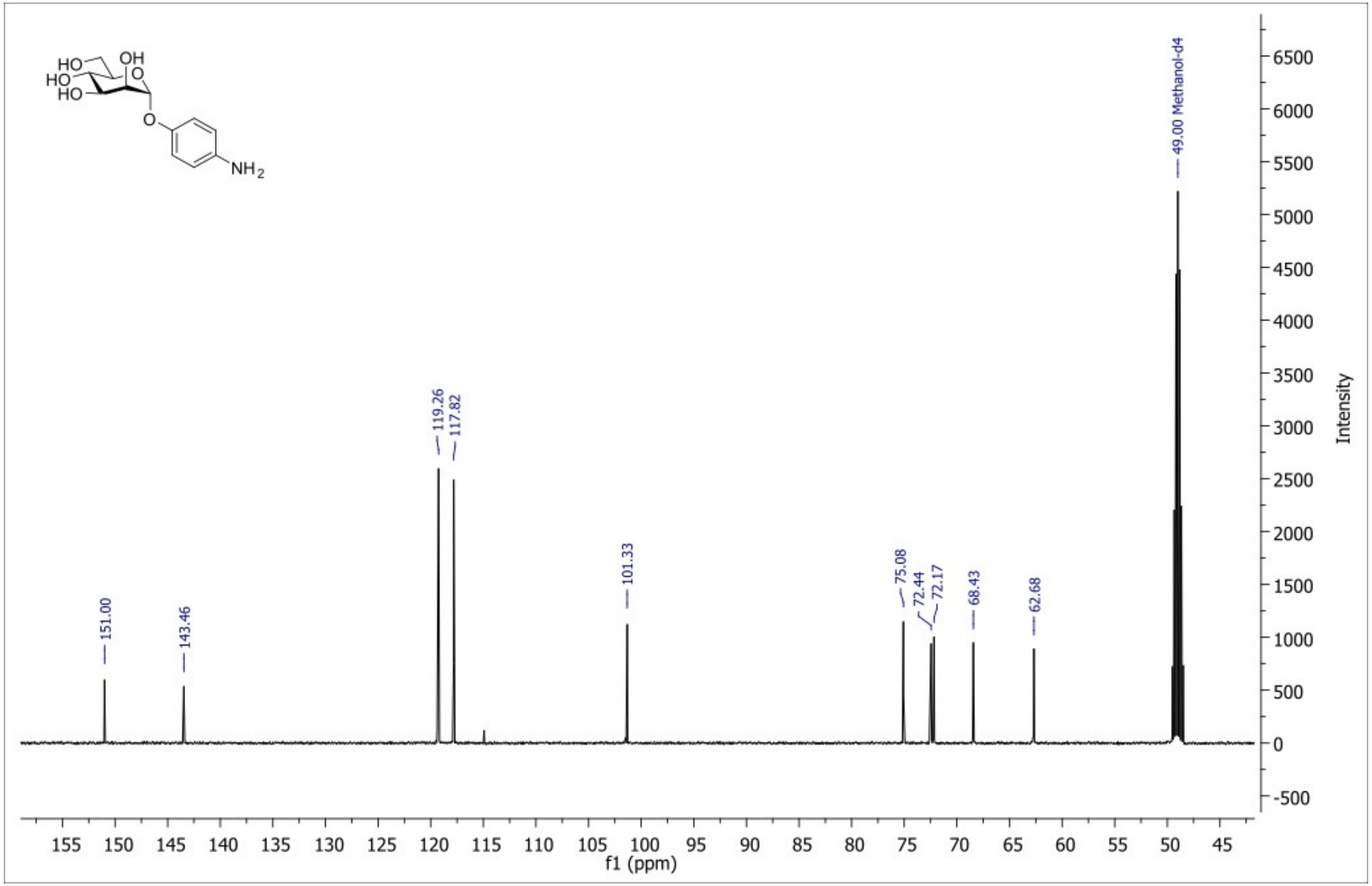

Figure S32. Carbon NMR of **S5**

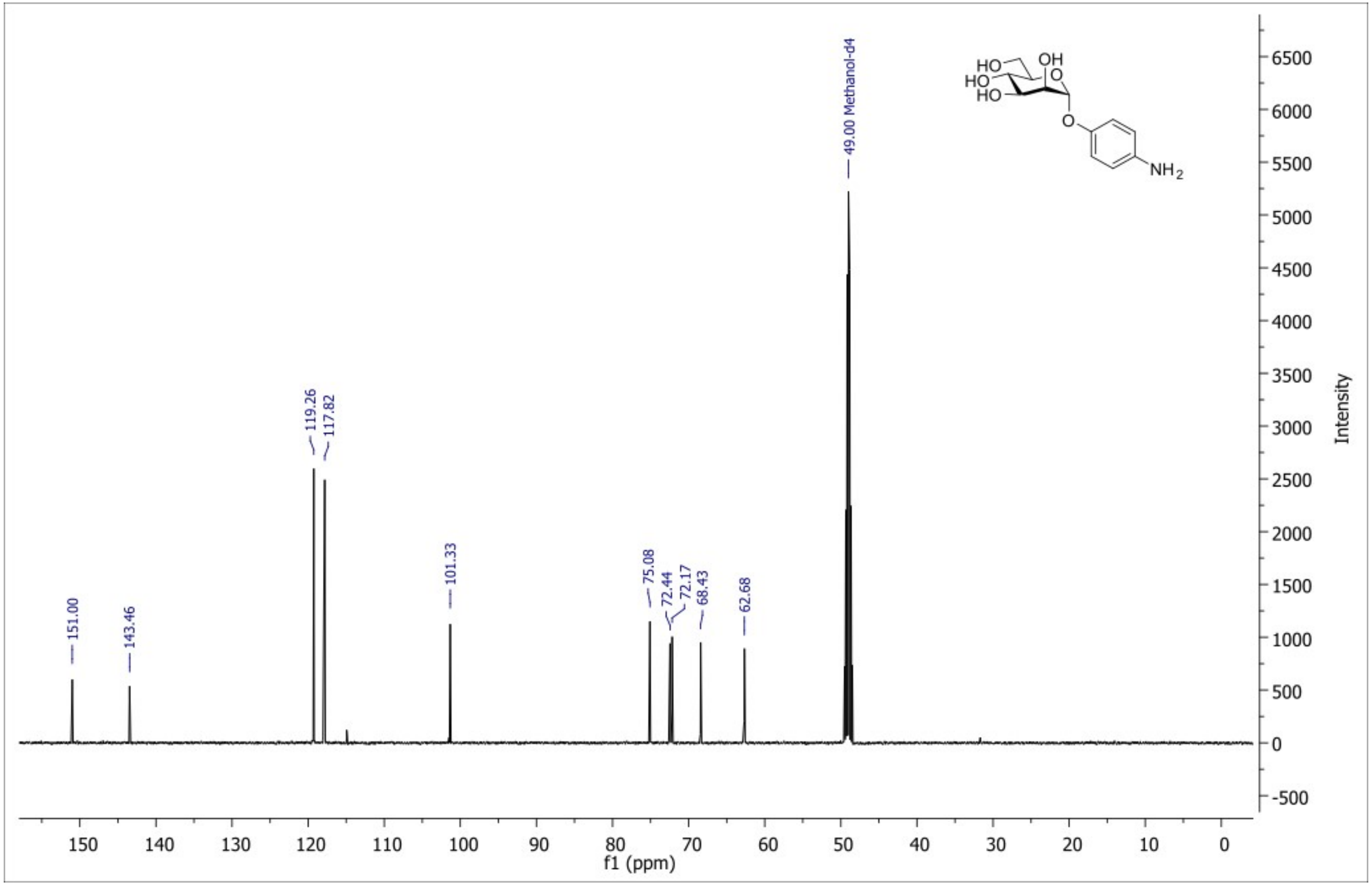

Figure S33. Carbon NMR of **S5**

Mass Spectrum of 4-Aminophenyl α-D-mannopyranoside **S5**

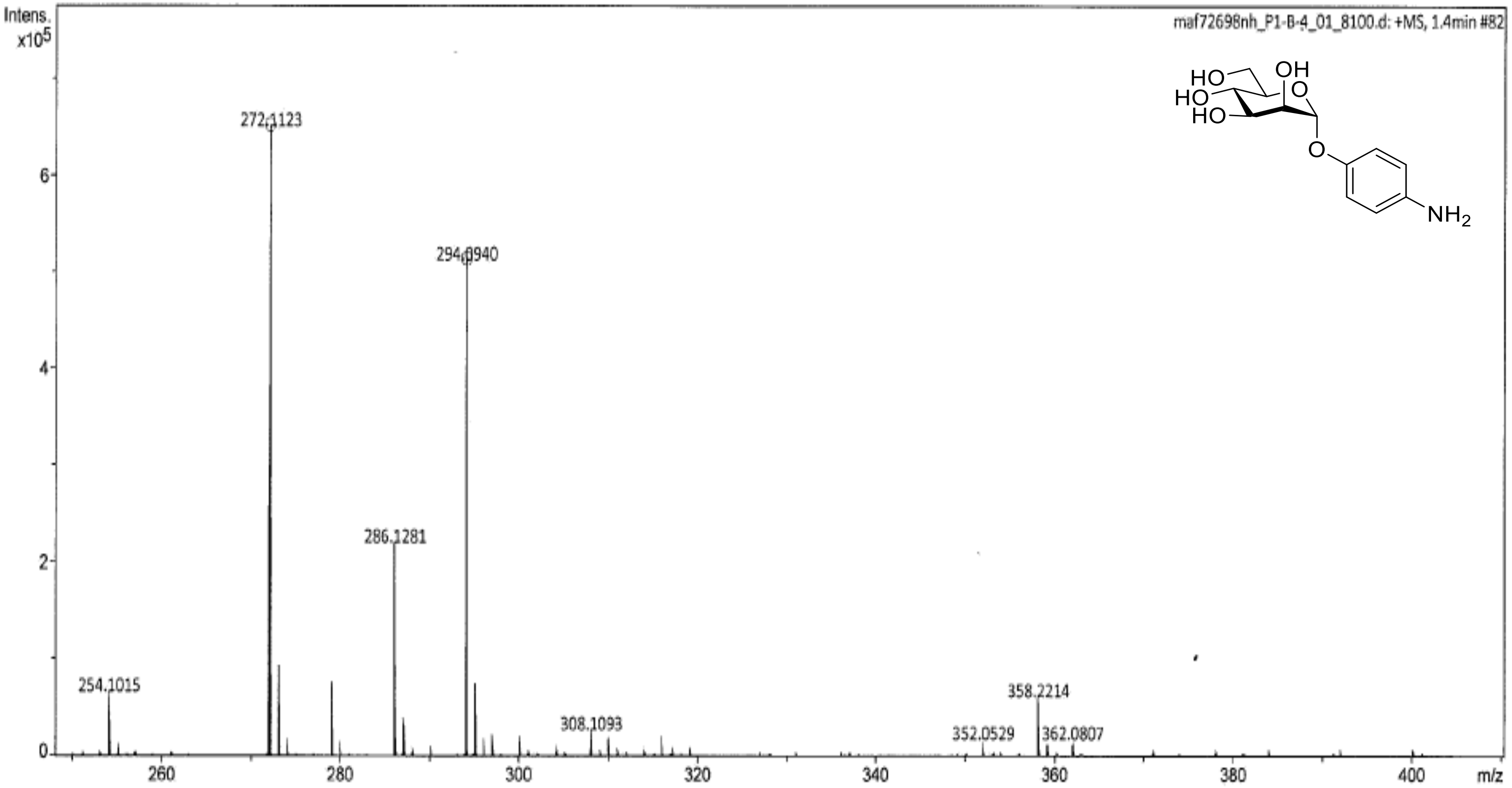

Meas. m/z	#	Ion Formula	m/z	err [ppm]	err [mDa]	mSigma	Mean err [ppm]
272.1123	1	C12H18NO6	272.1129	2.2	0.6	2.7	2.1
294.0940	1	C12H17NNaO6	294.0948	2.8	0.8	1.3	3.7

Figure S34. Mass spectrum of **S5**

FR-IR (ATR) Spectrum of 4-Aminophenyl α-D-mannopyranoside **S5**

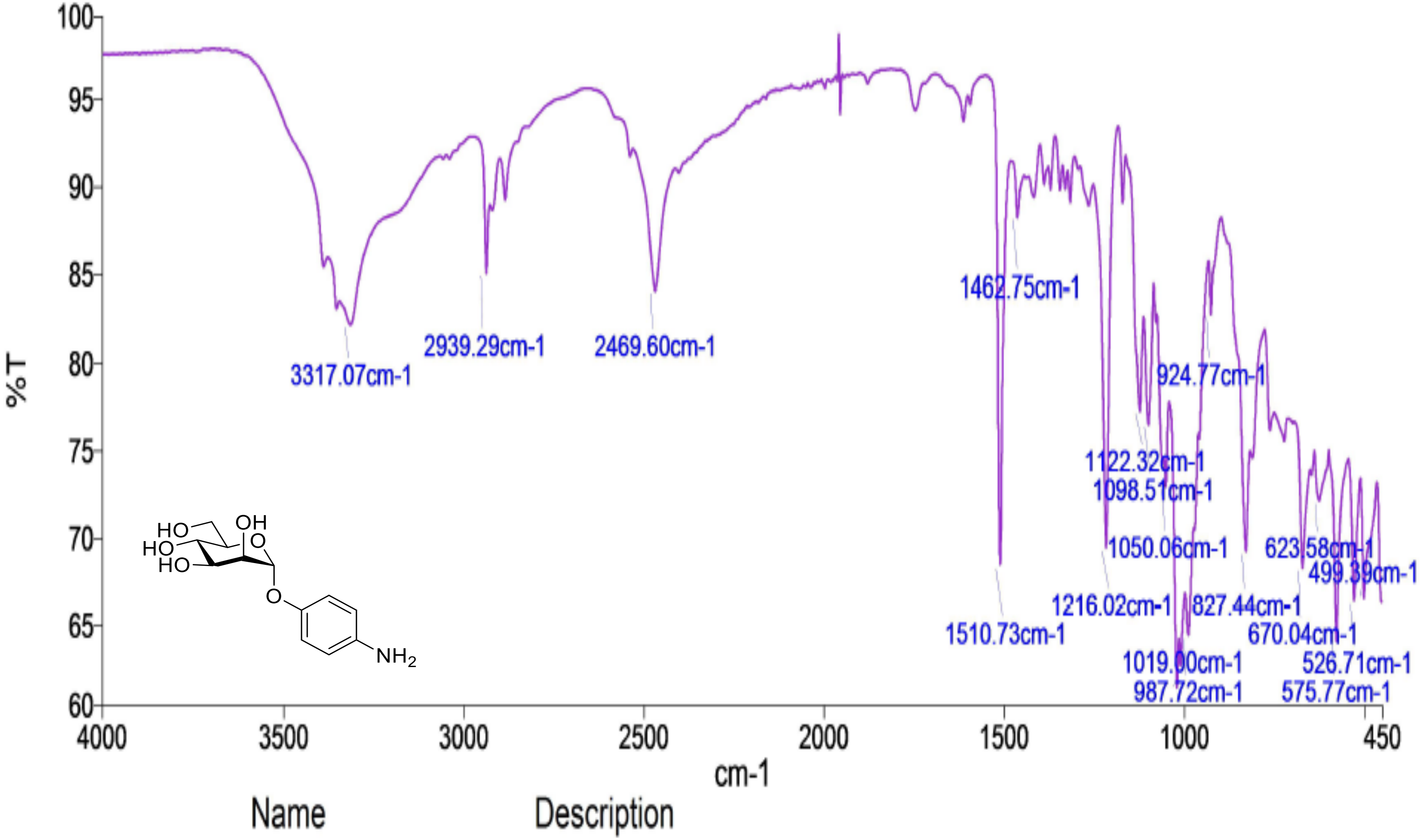

Figure S35. IR spectrum of **S5**

Step 4. Synthesis of p-[N-(2-Methoxy-3,4-dioxocyclobut-1-enyl)aminophenyl α-D-mannopyranoside 3[7]
3,4-dimethoxycyclobut-3-ene-1,2-dione **S6** (7.1 g, 0.05 mols) was added to **S5** (13.6 g, 0.05 mols, 1 eq) in dry methanol and the reaction mixture was stirred at RT for 4 h to afford a white cloudy solution containing a precipitate. The precipitate was isolated by filtration, and was dried *in vacuo* to yield a white solid of **3** (16.6 g, 0.043 mols, 87%).

^{1}H NMR (500 MHz, DMSO) δ 10.66 (s, 1H, NH), 7.25 (s, 2H, HAr), 7.07 (dd, J_{HAr} = 9.0 Hz, J_{HAr} = 1.92 Hz, 2H, HAr), 5.31 (d, $J_{1,2}$ = 1.5 Hz, 1H, H-1), 4.99 (d, $J_{2,OH}$ = 4.5 Hz, 1H, OH (H-2)), 4.81 (d, $J_{4,OH}$ = 5.6 Hz, OH (H-4)), 4.72 (d, $J_{3,OH}$ = 5.9 Hz, 1H, OH (H-3)), 4.44 (dd, $J_{6a,OH}$ = 6.0 Hz, $J_{6b,OH}$ = 6.0 Hz, OH (H-6)), 4.36 (s, 3H, CH_3), 3.81 (m, 1H, H-2), 3.68-3.63 (m, 1H, H-3), 3.60 (ddd, J_{6a} = 11.6 Hz, J_{6a} = 5.7 Hz, $J_{6a,5}$ = 1.7 Hz, 1H, H-6a), 3.50-3-43 (m, 2H, H-4, H-6b), 3.40 (ddd, J_5 = 9.2 Hz, J_5 = 5.9 Hz, $J_{5,6a}$ = 1.7 Hz, 1H, H-5).

^{13}C NMR (126 MHz, DMSO) δ 183.9 (1C, C=O), 169.3 (1C, C=O), 153.7 (1C, C), 132.7 (1C, C), 121.5, 119.0, 117.0, 115.0 (CAr), 99.7 (1C, C-1), 75.4 (1C, C-5), 71.1, (1C, C-3), 70.5 (1C, C-2), 67.2 (1C, C-4), 61.5 (1C, C-6), 60.9 (1C, CH_3).

(ESI)HRMS – $C_{17}H_{19}O_9NNa^+$ ($[M+Na]^+$) requires m/z 404.0958: found m/z 404.0947

[α]D^{25} = + 111.22 (*c* 1, DMSO)

FT-IR (ATR) – 3438, (O-H), 3264 (N-H), 3077 (C-H), 2940 (C-H), 1809 (C=O), 1711 (C=O), 1626 (C=C), 1594 (C=C), 1366 (C-O), 1088 (C-O).

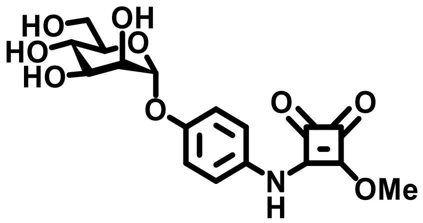

Figure S36. Structure of p-[N-(2-Methoxy-3,4-dioxocyclobut-1-enyl)aminophenyl α-D-mannopyranoside **3**

Hydrogen NMR Spectrum of Synthesis of p-[N-(2-Methoxy-3,4-dioxocyclobut-1-enyl)aminophenyl α-D-mannopyranoside **3**

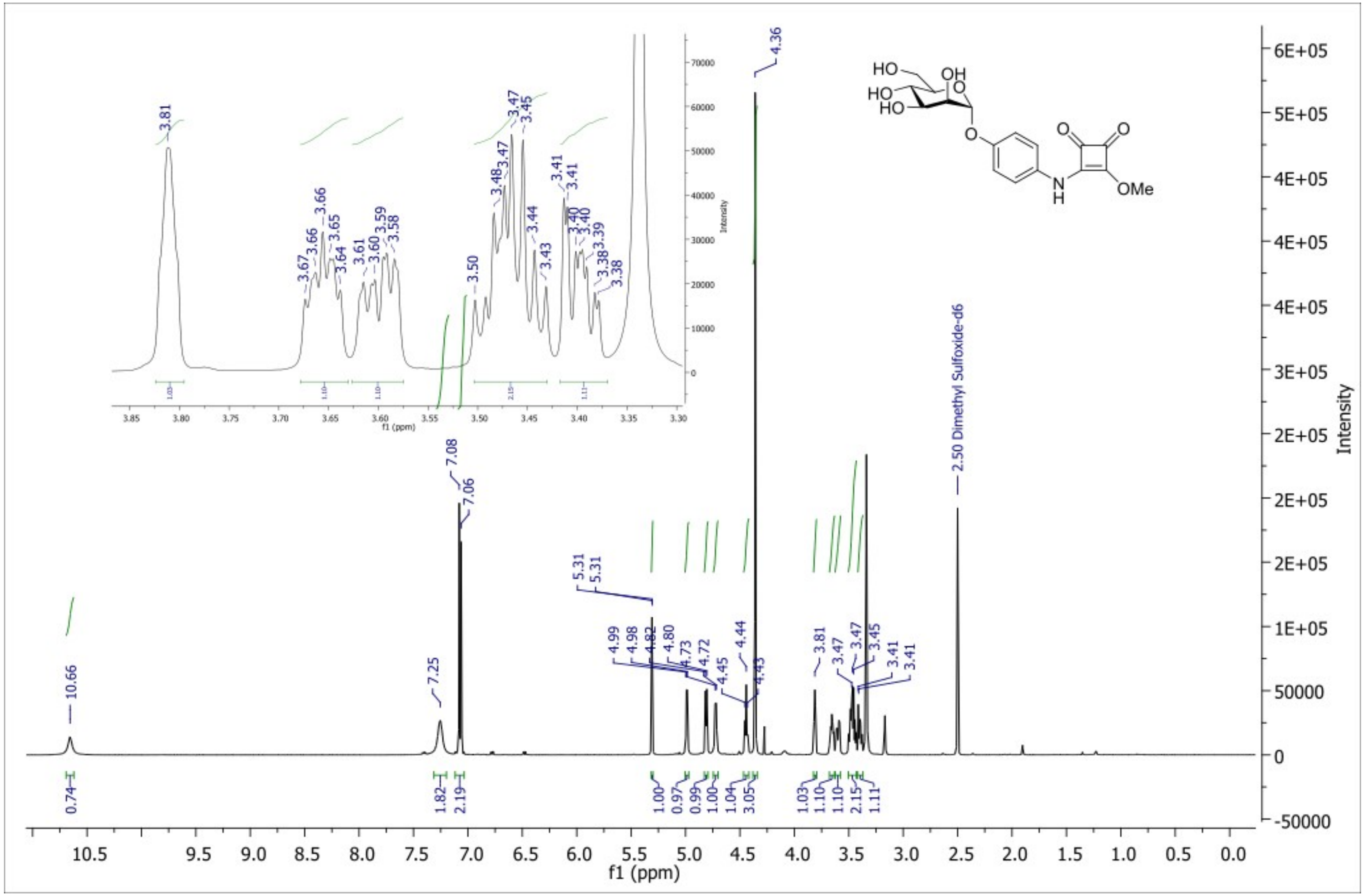

Figure S37. Hydrogen NMR spectrum of **3**

Carbon NMR Spectrum of Synthesis of p-[N-(2-Methoxy-3,4-dioxocyclobut-1-enyl)aminophenyl α-D-mannopyranoside **3**

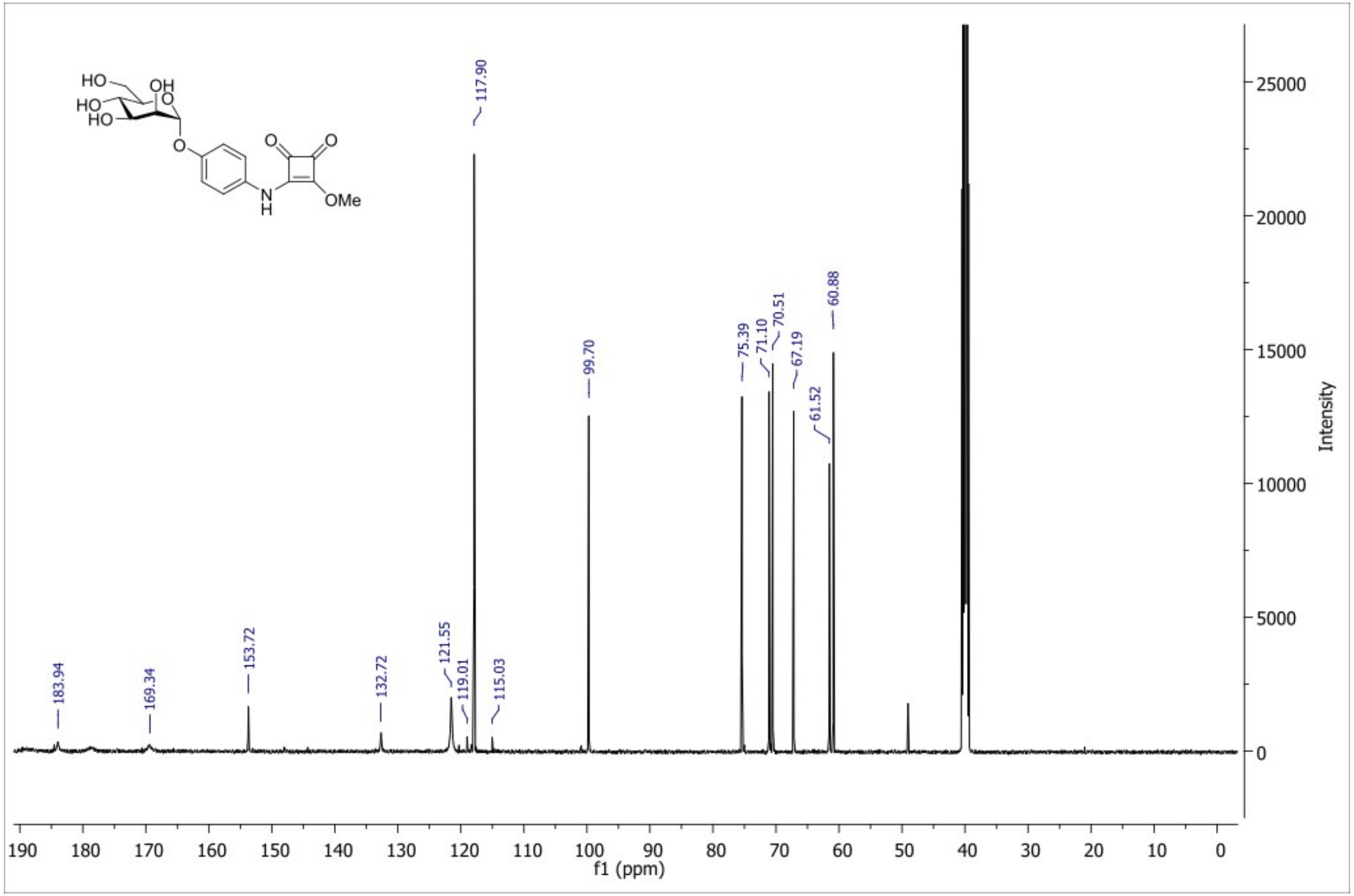

Figure S38. Carbon NMR spectrum of **3**

Mass Spectrum of Synthesis of p-[N-(2-Methoxy-3,4-dioxocyclobut-1-enyl)aminophenyl α-D-mannopyranoside **3**

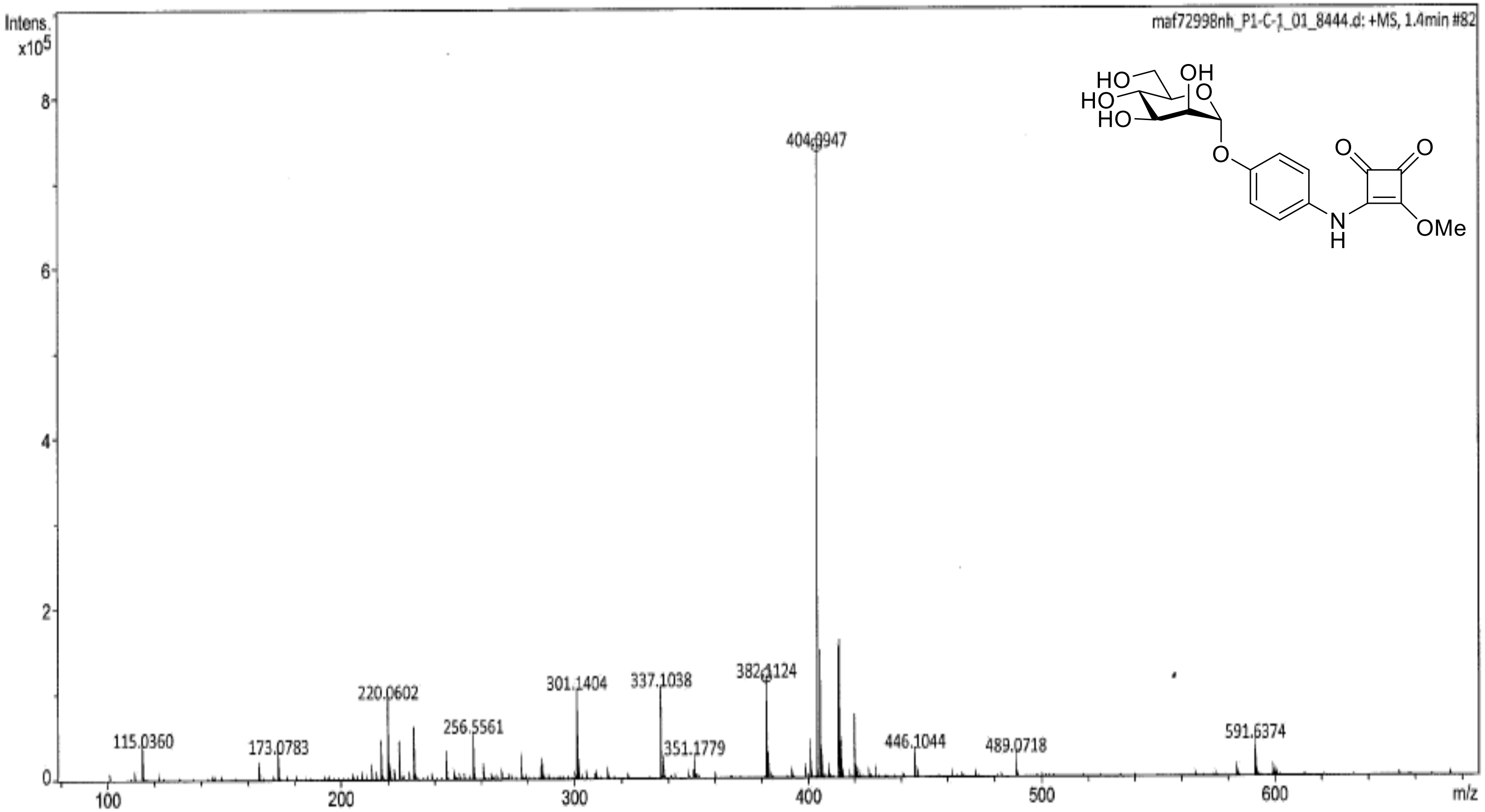

Meas. m/z	#	Ion Formula	m/z	err [ppm]	err [mDa]	mSigma	Mean err [ppm]
382.1124	1	C17H20NO9	382.1133	2.2	0.8	8.1	2.2
404.0947	1	C17H19NNaO9	404.0952	1.1	0.5	0.7	1.1

Figure S39. Mass spectrum of **3**

FT-IR (ATR) Spectrum of Synthesis of p-[N-(2-Methoxy-3,4-dioxocyclobut-1-enyl)aminophenyl α-D-mannopyranoside **3**

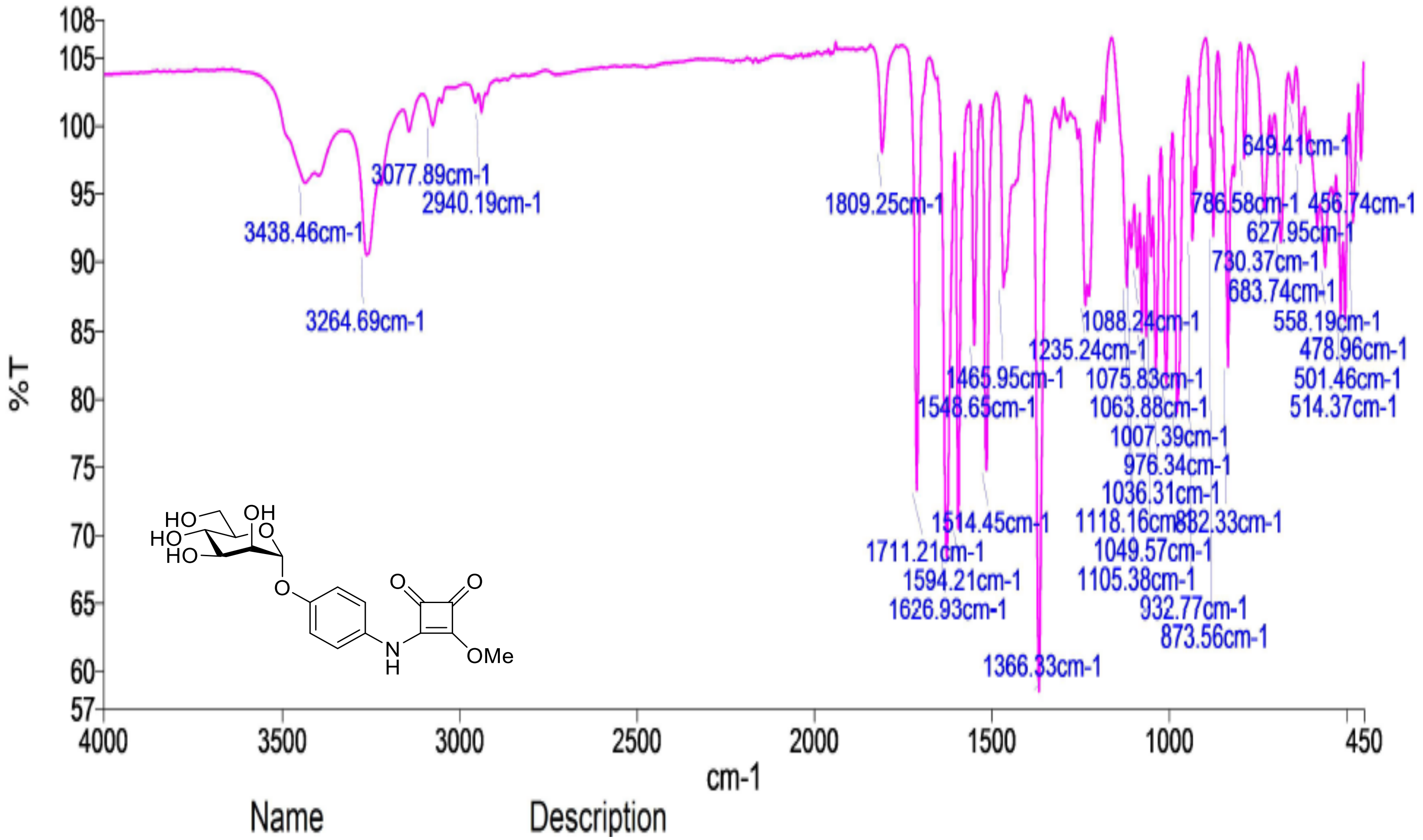

Figure S40. IR spectrum of **3**

Step 5. Synthesis of mannose azide 4

NEt_3 (0.12 mL, 1.3 mmols, 1.5 eq) was added dropwise to a solution of **3** (0.33 g, 0.87 mmols) and 1-amino-2-azidoethane **S7** (0.15 g, 1.7 mmols. 2 eq) in dry MeOH (20 mL). The reaction was stirred at RT for 16 h, affording a light-yellow solution. The solution was neutralized with dowex and concentrated *in vacuo* to yield a semi-crude yellow solid confirmed by mass spectrometry and NMR analysis to contain a 4:1 mixture of **mannose azide 4** to starting material (0.26 g, ≤ 0.75 mmols, ≤ 67%). This solid was used crude in peptide synthesis.

^{1}H NMR peaks for product (500 MHz, DMSO) δ 7.36 (d, J_{HAr} = 8.8 Hz, 2H, HAr), 7.05 (d, J_{HAr} = 8.8 Hz, 2H, HAr), 5.28 (d, $J_{1,2}$ = 1.6 Hz, 1H, H-2), 3.81 (m, 1H, H-2), 3.77 (dd, J_{CH2} = 6.01 Hz, 2H, CH_2), 3.66 (dd, J = 9.3 Hz, J = 3.5 Hz, 1H, H-3), 3.61-3.54 (m, 3H, H-6a, CH_2), 3.51-3.40 (m, 3H, H-6b, H-4, H-5).

(ESI)HRMS – $C_{18}H_{21}N_5NaO_8^+$ ($[M+Na]^+$) requires m/z 458.1288: found m/z 458.1286

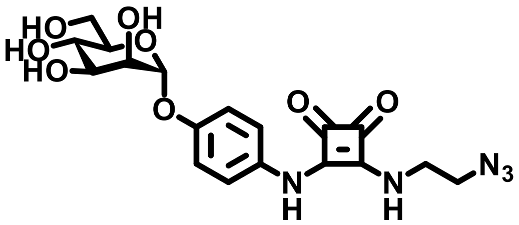

Figure S41. Structure of mannose azide **4**

Hydrogen NMR Spectrum of mannose azide **4**

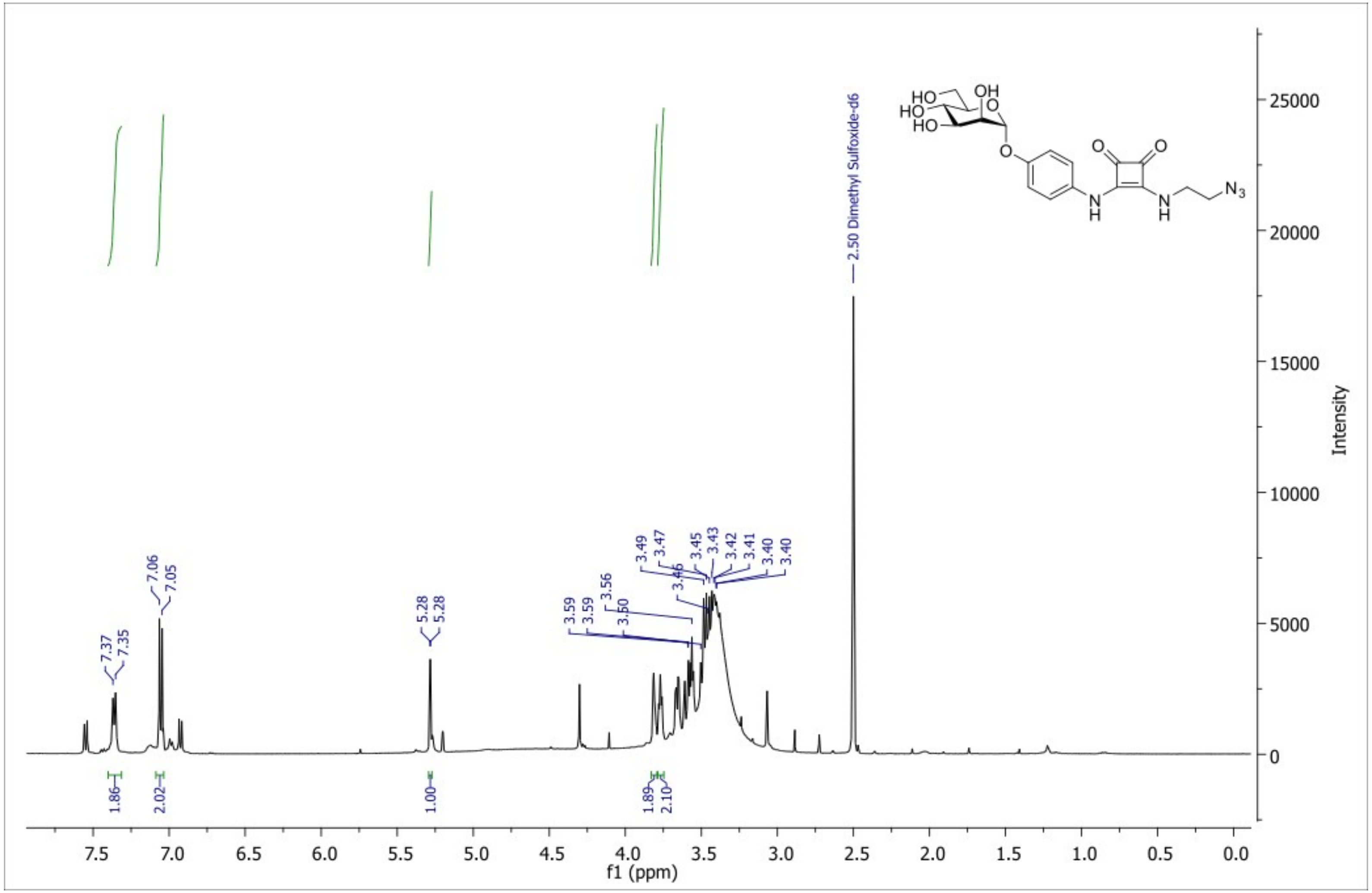

Figure S42. Hydrogen NMR of **4**

Mass Spectrum of mannose azide **4**

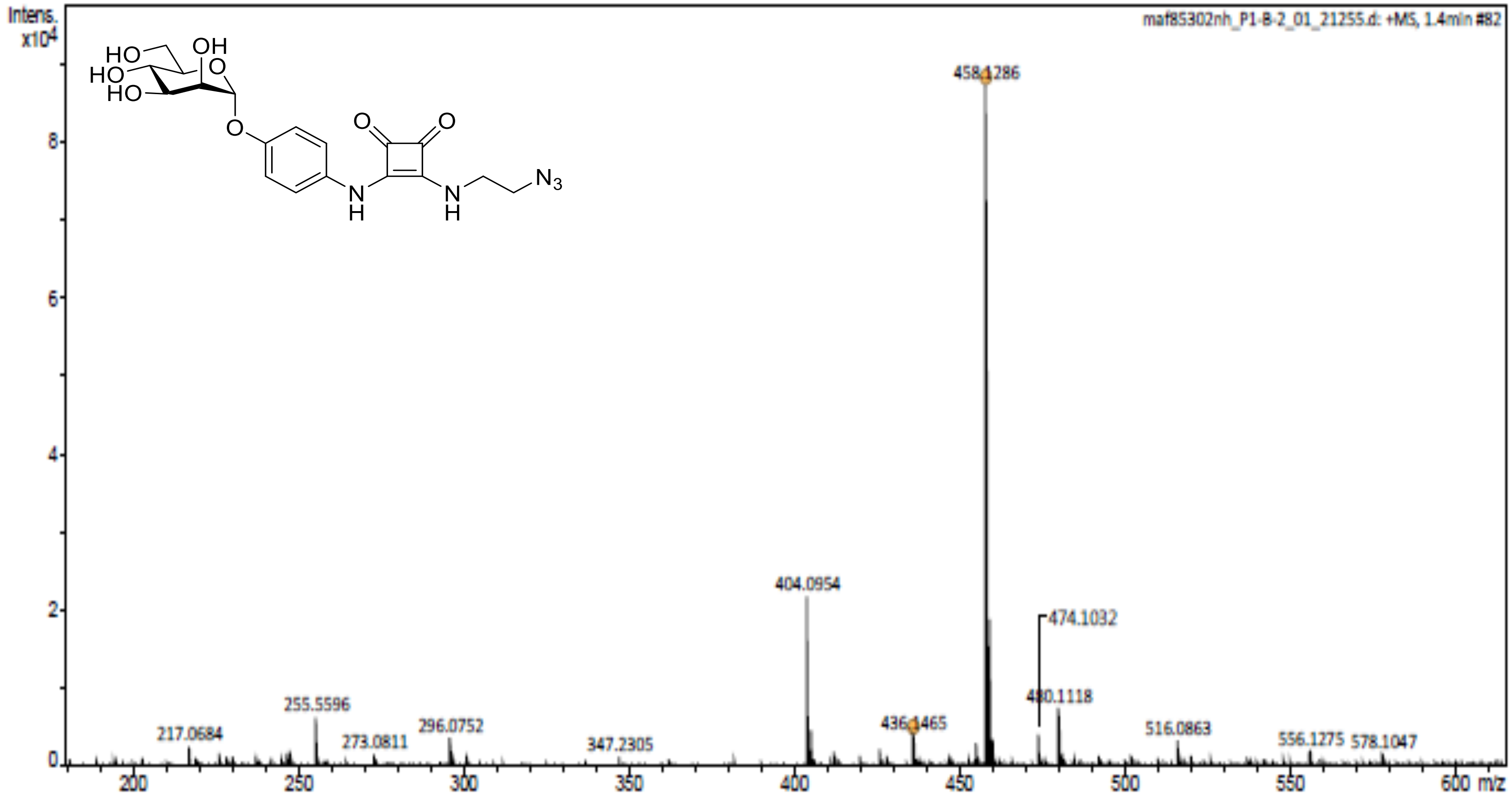

Meas. m/z	#	Ion Formula	m/z	err [ppm]	err [mDa]	mSigma	Mean err [ppm]
436.1465	1	C18H22N5O8	436.1463	-0.6	-0.3	16.3	0.5
458.1286	1	C18H21N5NaO8	458.1282	-0.9	-0.4	1.4	-0.5

Figure S43. Mass spectrum of **4**

Chemical Synthesis of additional compounds

Synthesis of 3,4-dimethoxycyclobut-3-ene-1,2-dione S6[8]

Trimethyl orthoformate (3.9 mL, 36 mmols, 2 eq) was added to a solution of 3,4-dihydroxy-3-cyclobutene-1,2-diol (2 g, 18 mmols, 1 eq) in methanol (100 mL). The reaction mixture was refluxed at 56 °C for 24 h, affording a colourless solution. The solution was concentrated *in vacuo* to yield a crude colourless oil. This oil was purified by column chromatography, eluting with a graduated solvent system of 3:1 Hex : EtOAc to 5 : 1 DCM : MeOH. This yielded white crystals of 3,4-dimethoxycyclobut-3-ene-1,2-dione **S6** (2.0 g, 0.014 mols, 78%).

^{1}H NMR (500 MHz, $CDCl_3$) δ 4.34 (s, 6H, OCH_3).

^{13}C NMR (126 MHz, $CDCl_3$) δ 189.2 (2C, C=O), 184.5 (2C, C-3,4), 61.0 (2C, CH_3).

(ESI)HRMS – $C_6H_6NaO_4^+$ ($[M+Na]^+$) requires m/z 165.0998: found m/z 165.0158

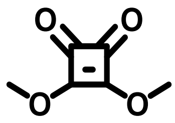

Figure S44. Structure of **S6**

Hydrogen NMR Spectrum of 3,4-dimethoxycyclobut-3-ene-1,2-dione **S6**

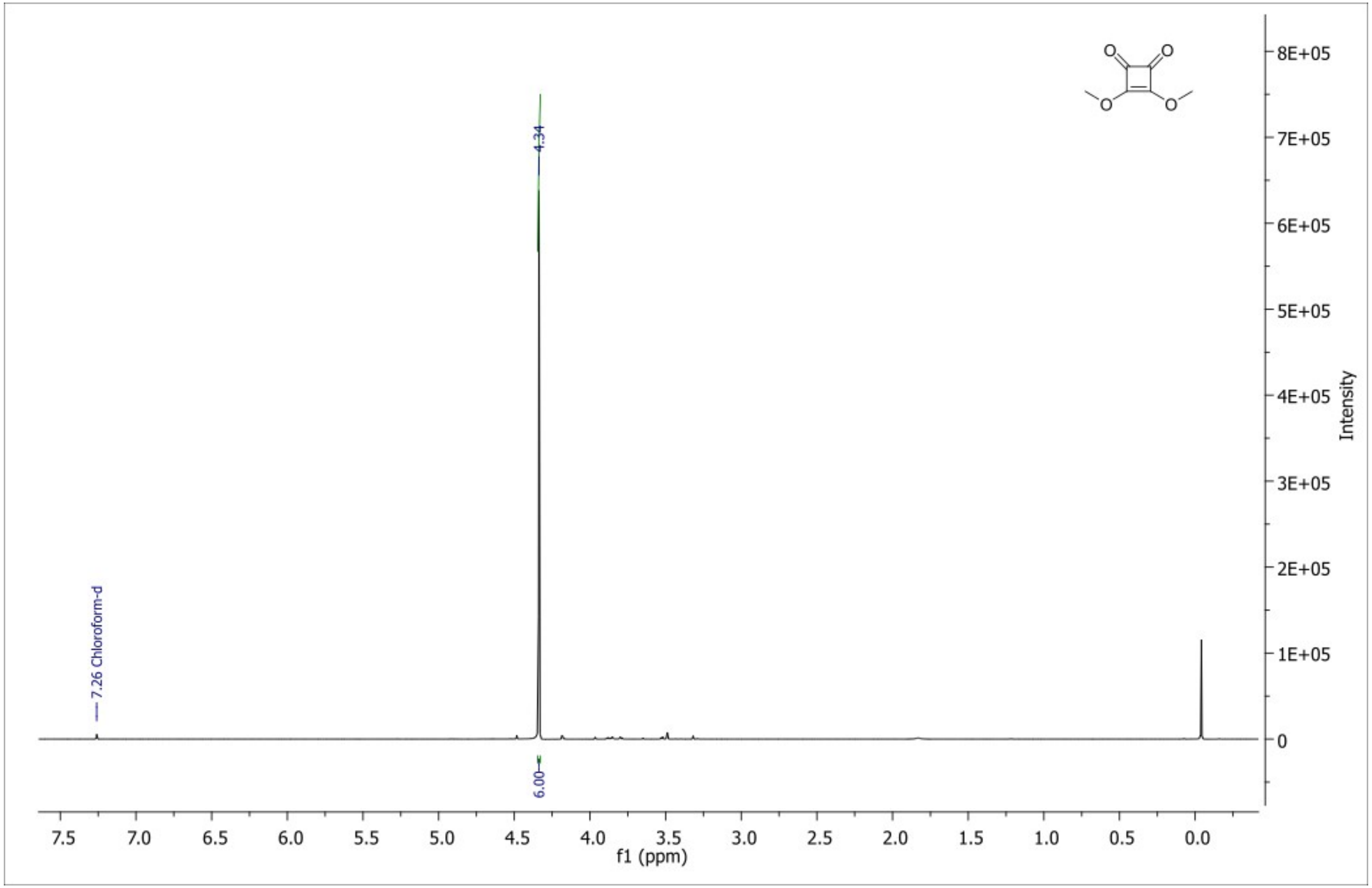

Figure S45. Hydrogen NMR spectrum of **S6**

Carbon NMR Spectrum of 3,4-dimethoxycyclobut-3-ene-1,2-dione **S6**

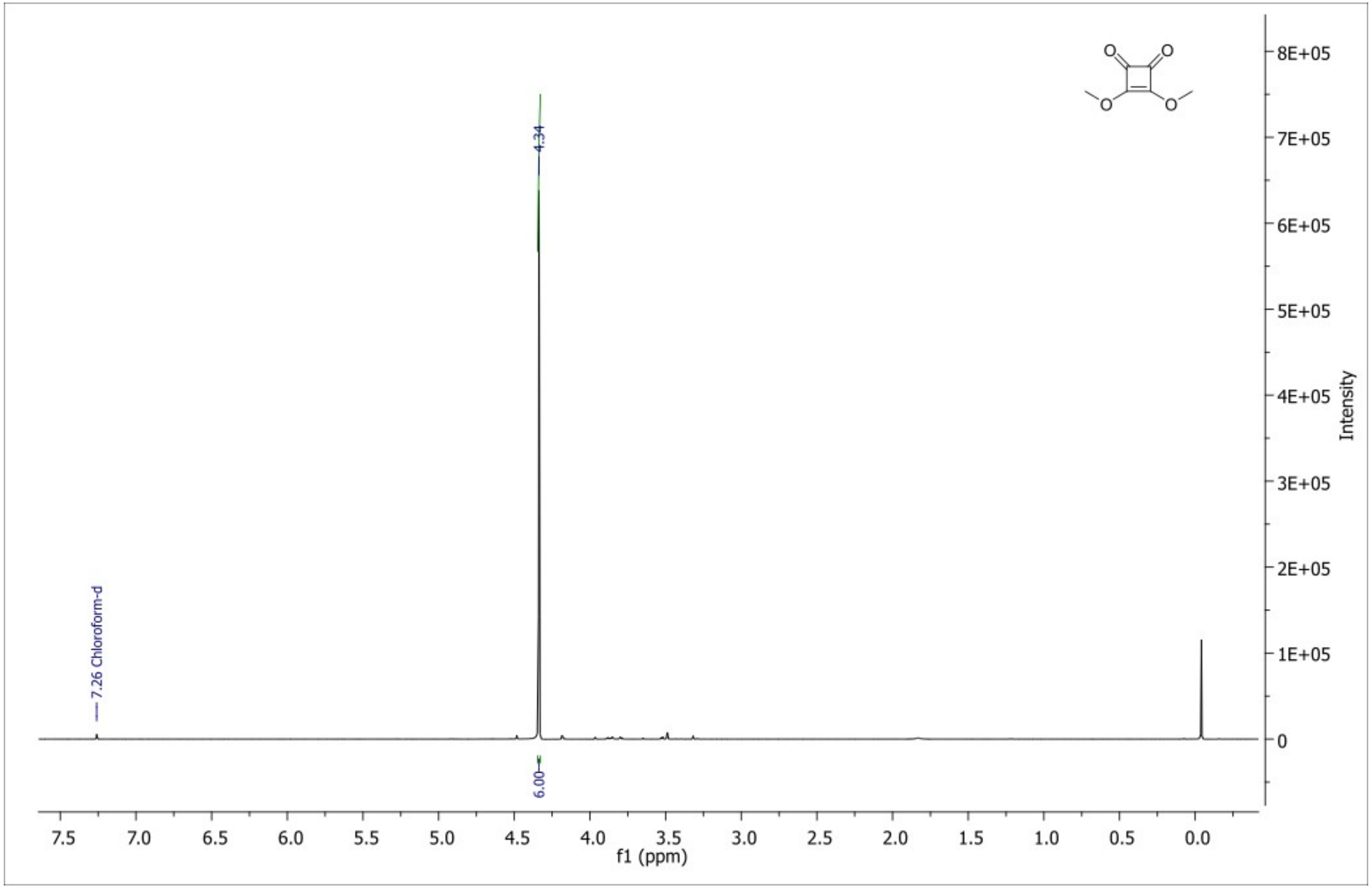

Figure S46. Carbon NMR spectrum of **S6**

Mass Spectrum of 3,4-dimethoxycyclobut-3-ene-1,2-dione **S6**

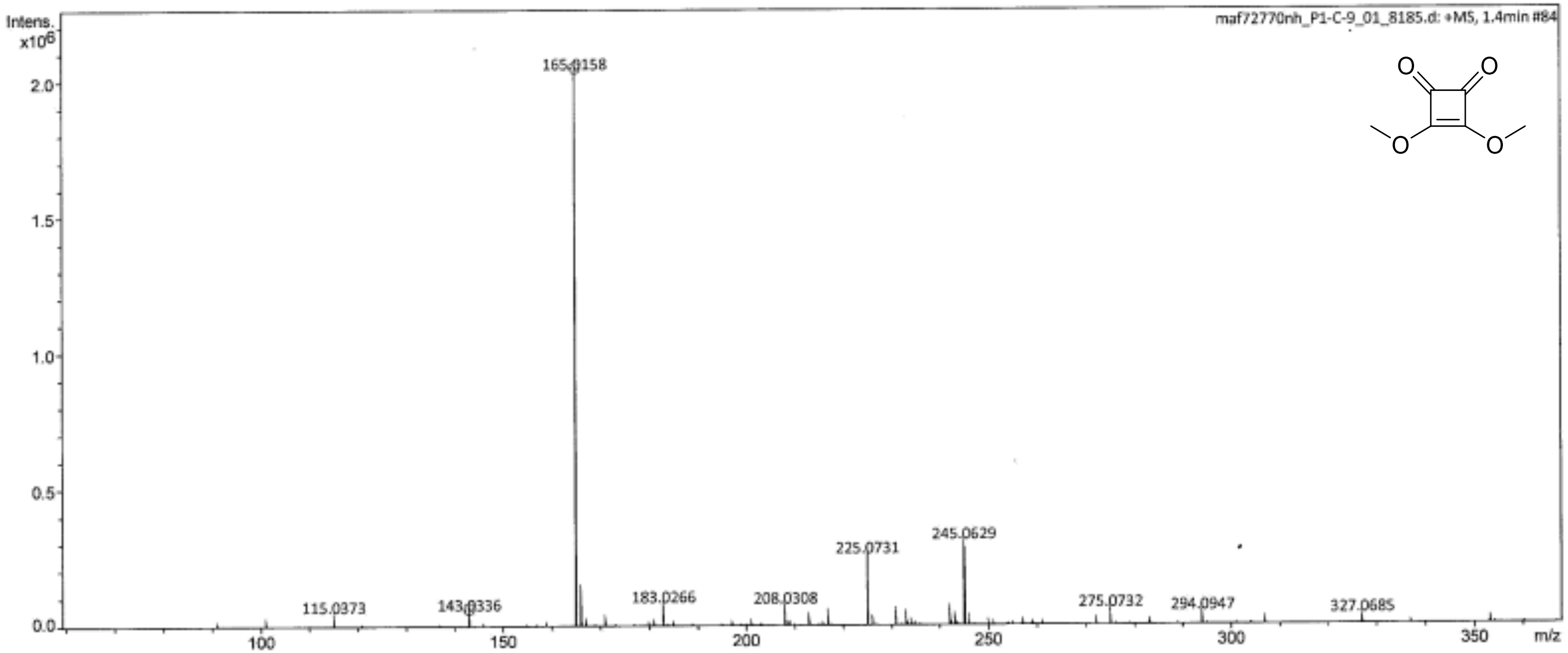

Meas. m/z	#	Ion Formula	m/z	err [ppm]	err [mDa]	mSigma	Mean err [ppm]
143.0336	1	C6H7O4	143.0339	2.2	0.3	11.3	2.5
165.0158	1	C6H6NaO4	165.0158	0.5	0.1	1.2	0.9

Figure S47. Mass spectrum of **S6**

Synthesis of 1-amino-2-azidoethane S7[9]

2-Chloroethanamine-hydrochloride (3.0 g, 26 mmols) was dissolved in water (20 mL) and NaN_3 (5.0 g, 78 mmols, 3 eq) was added. The reaction mixture was stirred at 80 °C for 16 h, affording a clear solution. The solution was cooled to RT and NaOH was added. The solution was then concentrated *in vacuo* to afford a colourless oil. The oil was purified by column chromatography, eluting with a graduated solvent system of 100 % EtOAc to 5 : 1 DCM : MeOH. This afforded a clear oil of 1-amino-2-azidoethane **S7** (0.31 g, 3.6 mmols, 13%); R_f = 0.45 (5 : 1 DCM : MeOH).

^{1}H NMR (500 MHz, MeOD) δ 4.99 (m, 2H, NH_2), 3.68 (dd, *J* = 5.4 Hz, *J* = 5.4 Hz, 2H, CH_2), 3.06 (dd, *J* = *J* = 5.4 Hz, *J* = 5.4 Hz, 2H, CH_2).

^{13}C NMR (126 MHz, MeOD) δ 50.8 (1C, CH_2), 40.4 (1C, CH_2).

(ESI)HRMS – $C_2H_7N_4^+$ ($[M+H]^+$) requires m/z 86.1060: found m/z 87.0661

FT-IR (ATR)- 2925 (C-H), 2101 (N_3).

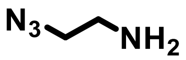

Figure S48. Structure of **S7**

Hydrogen NMR Spectrum of 1-amino-2-azidoethane **S7**

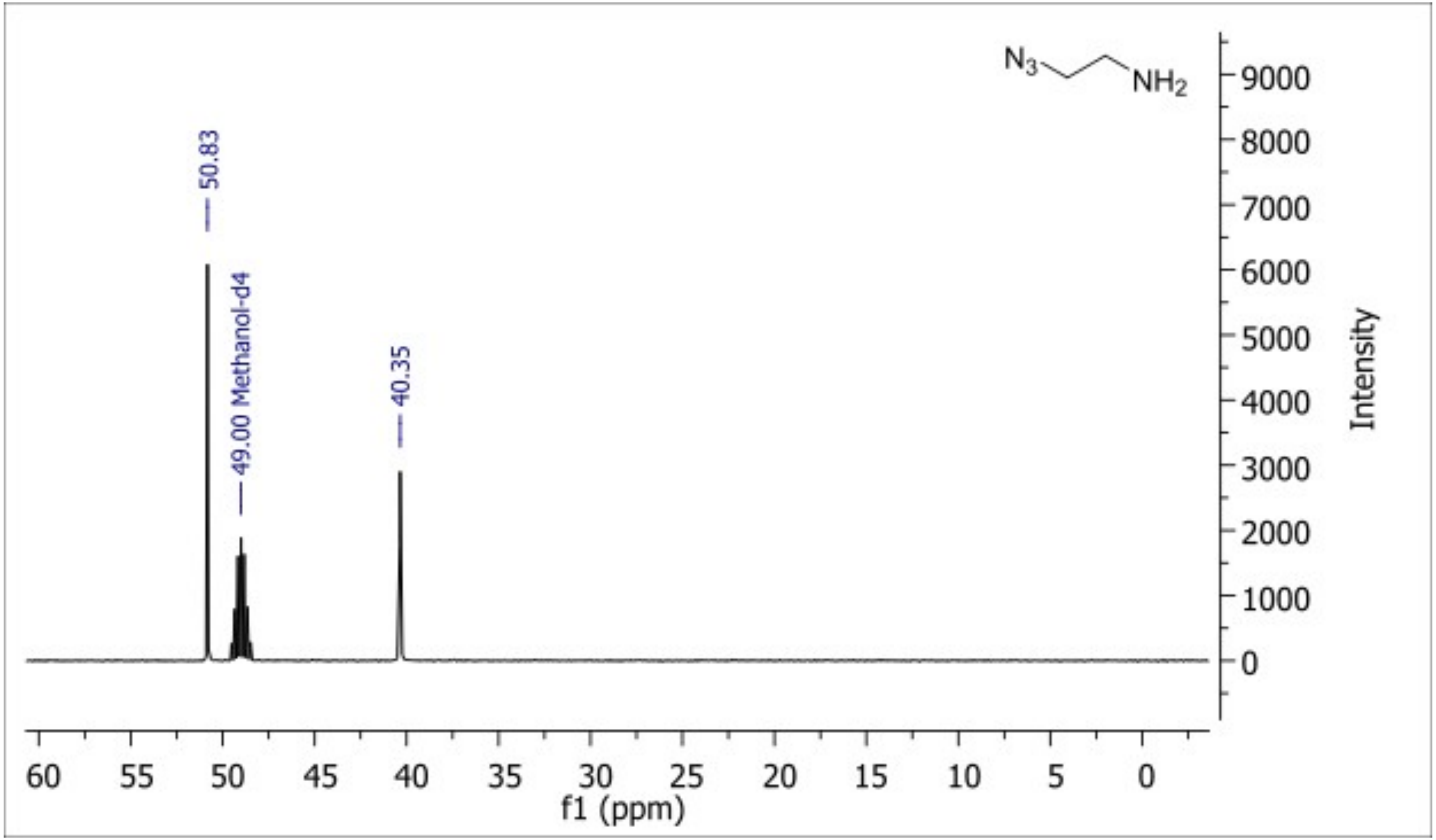

Figure S49. Hydrogen NMR spectrum of **S7**

Carbon NMR Spectrum of 1-amino-2-azidoethane **S7**

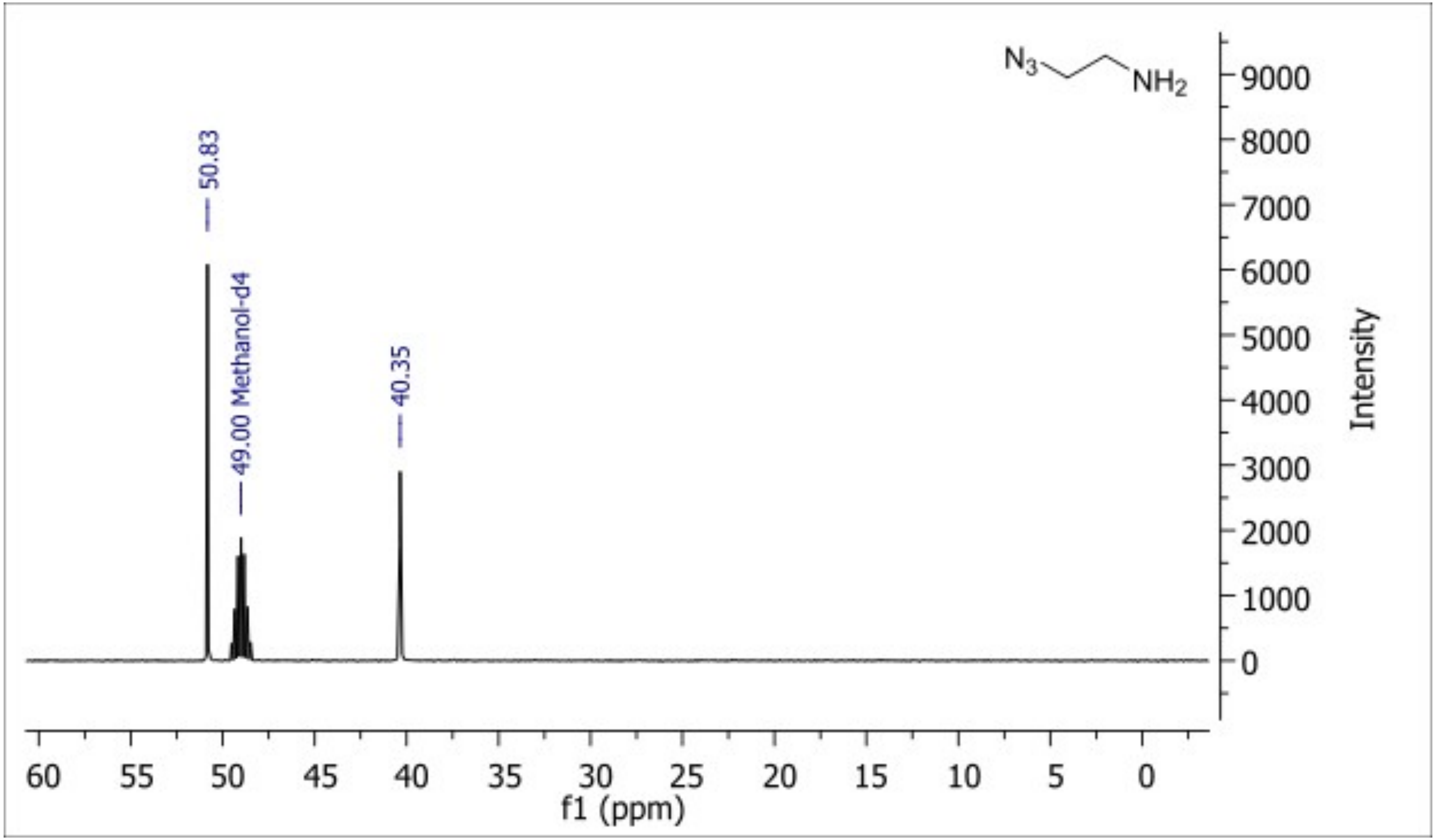

Figure S50. Carbon NMR spectrum of **S7**

Mass Spectrum of 1-amino-2-azidoethane **S7**

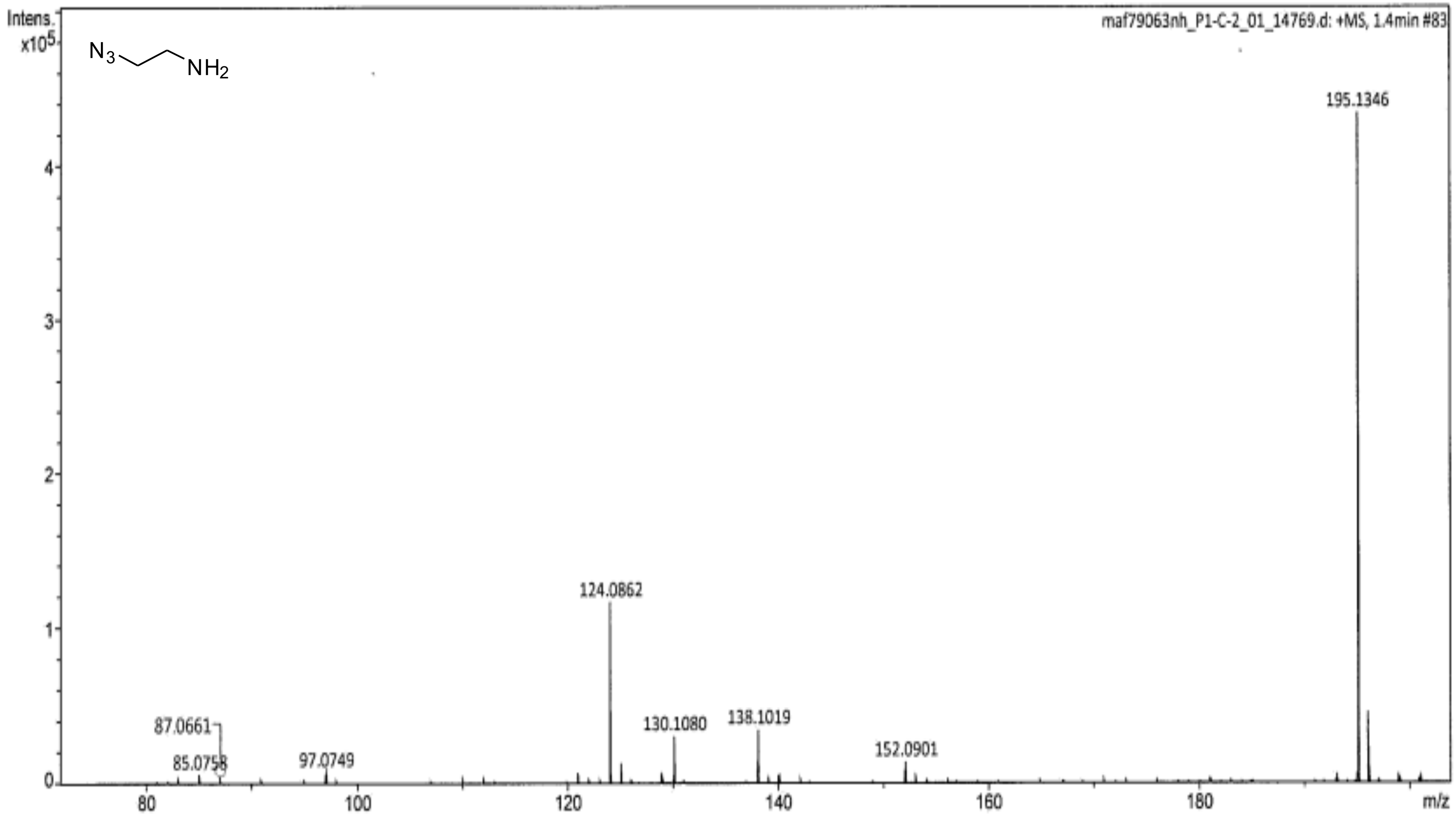

Meas. m/z	#	Ion Formula	m/z	err [ppm]	err [mDa]	mSigma	Mean err [ppm]
87.0661	1	C2H7N4	87.0665	5.4	0.5	n.a.	3.1

Figure S51. Mass spectrum of **S7**

FT-IR (ATR) Spectrum of 1-amino-2-azidoethane **S7**

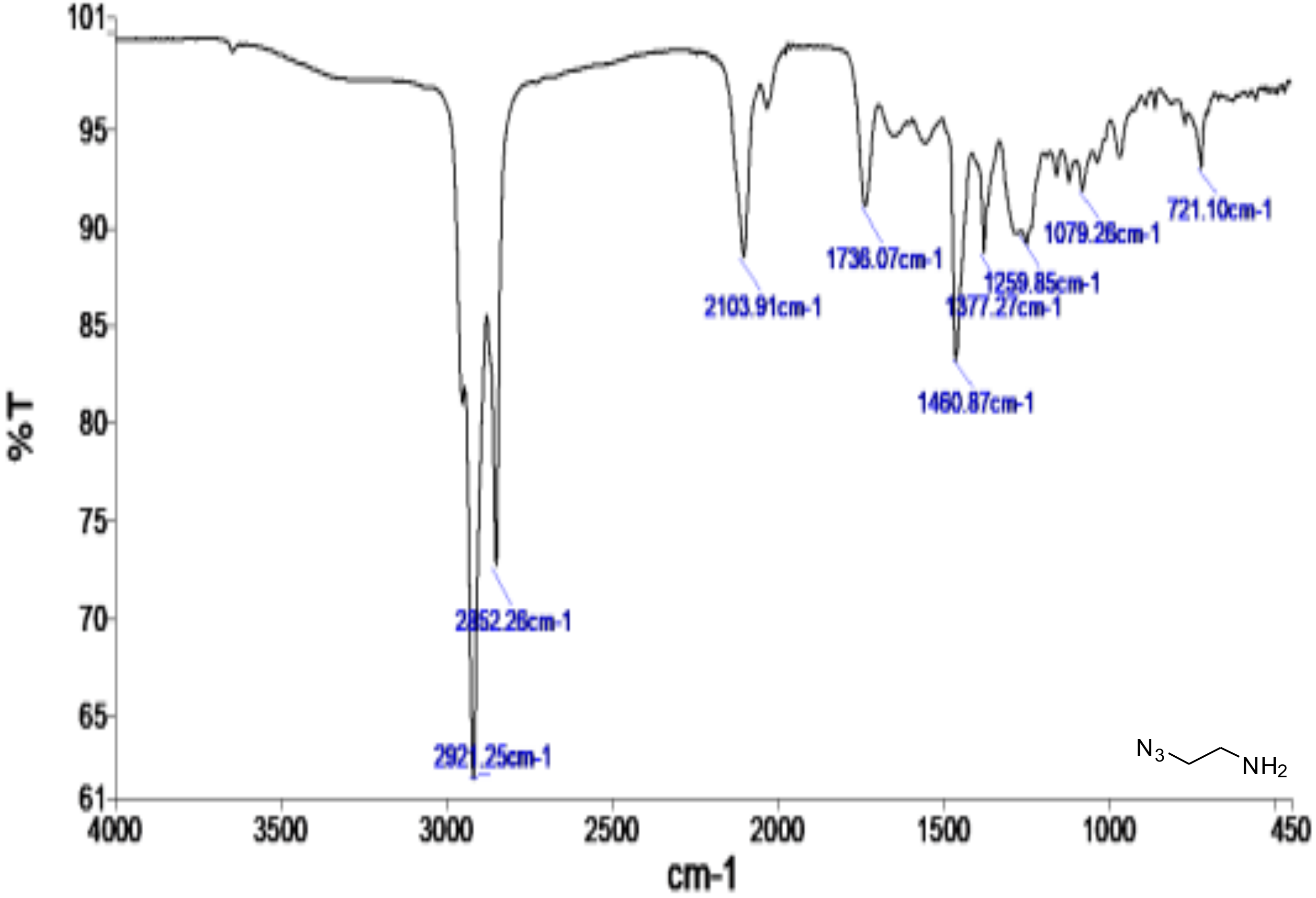

Figure S52. IR spectrum of **S7**

Synthesis of Protected Mannose-(Gly-Ser)$_1$-linked OPAL probe S8

Procedure for the synthesis of protected Mannsoe-(Gly-Ser)$_1$-linked OPAL probe **S8**

H-Gly-2-ClTrt resin was weighed out into an SPPS cartridge fitted with a PTFE stopcock, swollen in DMF for 30 minutes and then filtered.

DIPEA (98 µL, 0.56 mmols, 11 eq) was added to a solution of Fmoc-propargyl-Gly-OH (89 mg, 0.26 mmols, 5 eq) and HTCU (107 mg, 0.26 mmols, 5 eq) dissolved in the minimum volume of DMF. The resultant solution was then immediately added to the resin. The reaction mixture was gently agitated by rotation for 1 h and the resin was filtered off and washed with DMF (3 x 2 minutes with rotation). A solution of 20% piperdine in DMF was added to the resin and gently agitated by rotation for 2 minutes. The resin was filtered off and this process was repeated four more times, followed by washing with DMF (5 x 2 minutes with rotation).

DIPEA (98 µL, 0.56 mmols, 11 eq) was added to a solution of Fmoc-Gly-OH (79 mg, 0.26 mmols, 5 eq) and HTCU (107 mg, 0.26 mmols, 5 eq) dissolved in the minimum volume of DMF. The resultant solution was then immediately added to the resin. The reaction mixture was gently agitated by rotation for 1 h and the resin was filtered off and washed with DMF (3 x 2 minutes with rotation). A solution of 20% piperdine in DMF was added to the resin and gently agitated by rotation for 2 minutes. The resin was filtered off and this process was repeated four more times, followed by washing with DMF (5 x 2 minutes with rotation).

DIPEA (98 µL, 0.56 mmols, 11 eq) was added to a solution of Fmoc-Ser(tBu)-OH (101 mg, 0.26 mmols, 5 eq) and HTCU (107 mg, 0.26 mmols, 5 eq) dissolved in the minimum volume of DMF. The resultant solution was then immediately added to the resin. The reaction mixture was gently agitated by rotation for 1 h and the resin was filtered off and washed with DMF (3 x 2 minutes with rotation). A solution of 20% piperdine in DMF was added to the resin and gently agitated by rotation for 2 minutes. The resin was filtered off and this process was repeated four more times, followed by washing with DMF (5 x 2 minutes with rotation).

DIPEA (98 µL, 0.56 mmols, 11 eq) was added to a solution of OPAL linker building block[10] (102 mg, 0.26 mmols, 5 eq) and HTCU (107 mg, 0.26 mmols, 5 eq) dissolved in the minimum volume of DMF. The resultant solution was then immediately added to the resin. The reaction mixture was gently agitated by rotation for 1 h and the resin was filtered off and washed with DMF (5 x 2 minutes with rotation).

DIPEA (1 mL, 5.75 mmols, 55 eq) was added to a solution of mannose azide **4** (46 mg, 0.105 mmols, 1eq), sodium absorbate (31 mg, 0.156 mmols, 1.5 eq) and copper iodide (60 mg, 0.315 mmols, 3 eq) dissolved in DMF (2.5 mL) and the solution was added to resin. The reaction mixture was gently agitated by rotation for 12 h and the resin was filtered off and washed with H_2O (3 x 2 minutes with rotation), iPrOH (3 x 2 minutes with rotation), DMF (3 x 2 minutes with rotation), iPrOH (3 x 2 minutes with rotation), DMF (3 x 2 minutes with rotation).

The resin was washed with DCM (3 x 2 minutes with rotation) and MeOH (3 x 2 minutes with rotation). The resin was dried on a vacuum manifold and further dried on a high vacuum line overnight. A solution of cleavage cocktail (95:2.5:2.5 TFA:H_2O:triisopropylsilane) was added to the resin and gently agitated by rotation for 1 h. The reaction mixture was drained into ice-cold Et_2O and centrifuged at 4000 rpm at 4 °C until pelleted (ca 5-10 min). The supernatant was carefully decanted and then subsequently resuspended, centrifuged and supernatant decanted three more times. The precipitated peptide pellet was dissolved in water and lyophilised to obtain a powder of probe **S8**.

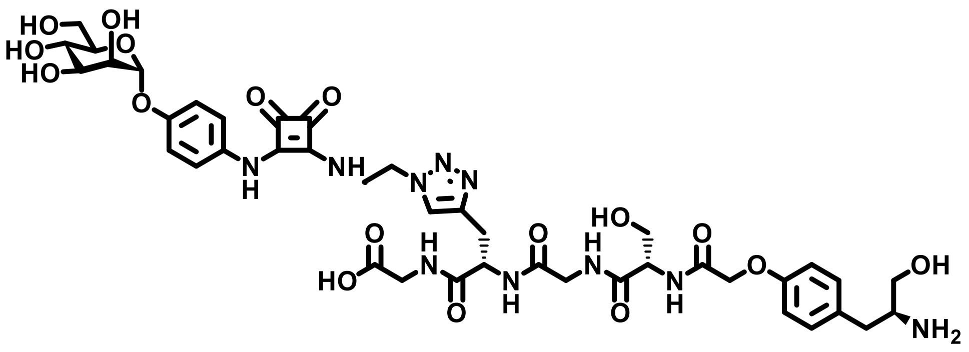

Figure S53. Structure of protected mannose-linked (Gly-Ser)$_1$ OPAL probe **S8**

LC and Mass Spectrum of protected Mannose-(Gly-Ser)$_1$-linked OPAL probe **S8**

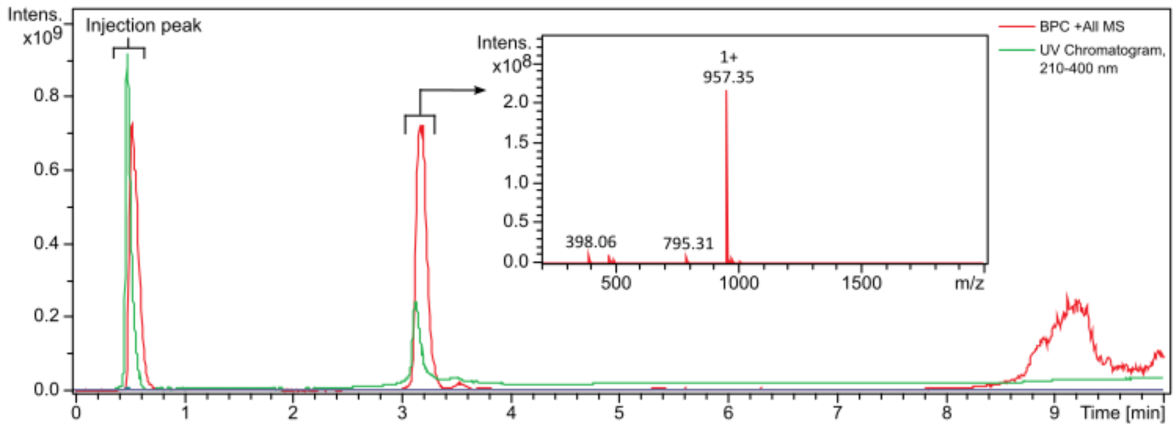

Figure S54. LC trace of protected Mannose-(Gly-Ser)1-linked OPAL probe **S8**

Synthesis of active Mannose-(Gly-Ser)$_1$-linked OPAL probe **5**

To a solution of Mannose-based inhibitor linked (Gly-Ser) click OPAL probe **S8** (500 µL, 14 mM, 6.7 mg, in 0.1 M PB, 0.1 M NaCl, pH = 7.0) was added $NaIO_4$ (63 µL, 112 mM, in 0.1 M PB, 0.1 M NaCl, pH = 7.0). The reaction was mixed thoroughly and allowed to sit for 3 minutes on ice in the dark. The solution was then loaded onto a solid phase extraction cartridge (Grace Davison Extract Clean, 8 ml reservoir, Fisher Scientific) equilibrated with water/acetonitrile. After initial washing with water, the product was eluted over a gradient of acetonitrile. The product was then diluted with water, and subsequently lyophilised to give **5** as a pale yellow, fluffy powder (4 mg, 62%) which was used crude in OPAL ligation.

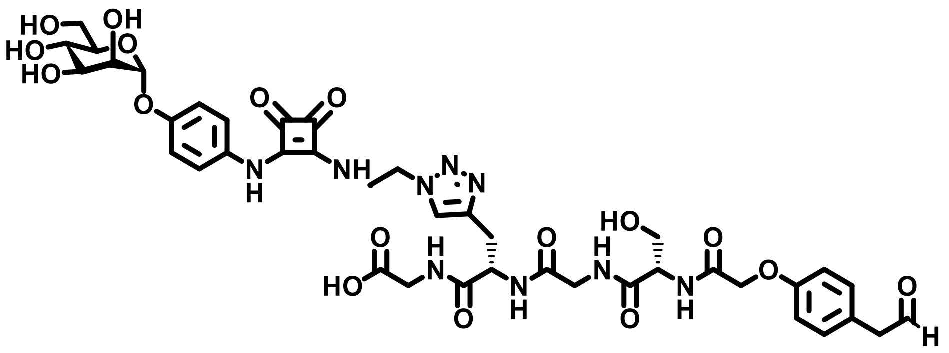

Figure S55. Structure of active mannose-linked (Gly-Ser)$_1$ OPAL probe **5**

Synthesis of a Mannose-(Gly-Ser)$_3$-linked OPAL probe **S9**

H-Gly-2-ClTrt resin was weighed out into an SPPS cartridge fitted with a PTFE stopcock, swollen in DMF for 30 minutes and then filtered.

DIPEA (98 µL, 0.56 mmols, 11 eq) was added to a solution of Fmoc-propargyl-Gly-OH (89 mg, 0.26 mmols, 5 eq) and HTCU (107 mg, 0.26 mmols, 5 eq) dissolved in the minimum volume of DMF. The resultant solution was then immediately added to the resin. The reaction mixture was gently agitated by rotation for 1 h and the resin was filtered off and washed with DMF (3 x 2 minutes with rotation). A solution of 20% piperdine in DMF was added to the resin and gently agitated by rotation for 2 minutes. The resin was filtered off and this process was repeated four more times, followed by washing with DMF (5 x 2 minutes with rotation).

DIPEA (98 µL, 0.56 mmols, 11 eq) was added to a solution of Fmoc-Gly-OH (79 mg, 0.26 mmols, 5 eq) and HTCU (107 mg, 0.26 mmols, 5 eq) dissolved in the minimum volume of DMF. The resultant solution was then immediately added to the resin. The reaction mixture was gently agitated by rotation for 1 h and the resin was filtered off and washed with DMF (3 x 2 minutes with rotation). A solution of 20% piperdine in DMF was added to the resin and gently agitated by rotation for 2 minutes. The resin was filtered off and this process was repeated four more times, followed by washing with DMF (5 x 2 minutes with rotation).

DIPEA (98 µL, 0.56 mmols, 11 eq) was added to a solution of Fmoc-Ser(tBu)-OH (101 mg, 0.26 mmols, 5 eq) and HTCU (107 mg, 0.26 mmols, 5 eq) dissolved in the minimum volume of DMF. The resultant solution was then immediately added to the resin. The reaction mixture was gently agitated by rotation for 1 h and the resin was filtered off and washed with DMF (3 x 2 minutes with rotation). A solution of 20% piperdine in DMF was added to the resin and gently agitated by rotation for 2 minutes. The resin was filtered off and this process was repeated four more times, followed by washing with DMF (5 x 2 minutes with rotation).

DIPEA (98 µL, 0.56 mmols, 11 eq) was added to a solution of Fmoc-Gly-OH (79 mg, 0.26 mmols, 5 eq) and HTCU (107 mg, 0.26 mmols, 5 eq) dissolved in the minimum volume of DMF. The resultant solution was then immediately added to the resin. The reaction mixture was gently agitated by rotation for 1 h and the resin was filtered off and washed with DMF (3 x 2 minutes with rotation). A solution of 20% piperdine in DMF was added to the resin and gently agitated by rotation for 2 minutes. The resin was filtered off and this process was repeated four more times, followed by washing with DMF (5 x 2 minutes with rotation).

DIPEA (98 µL, 0.56 mmols, 11 eq) was added to a solution of Fmoc-Ser(tBu)-OH (101 mg, 0.26 mmols, 5 eq) and HTCU (107 mg, 0.26 mmols, 5 eq) dissolved in the minimum volume of DMF. The resultant solution was then immediately added to the resin. The reaction mixture was gently agitated by rotation for 1 h and the resin was filtered off and washed with DMF (3 x 2 minutes with rotation). A solution of 20% piperdine in DMF was added to the resin and gently agitated by rotation for 2 minutes. The resin was filtered off and this process was repeated four more times, followed by washing with DMF (5 x 2 minutes with rotation).

DIPEA (98 µL, 0.56 mmols, 11 eq) was added to a solution of Fmoc-Gly-OH (79 mg, 0.26 mmols, 5 eq) and HTCU (107 mg, 0.26 mmols, 5 eq) dissolved in the minimum volume of DMF. The resultant solution was then immediately added to the resin. The reaction mixture was gently agitated by rotation for 1 h and the resin was filtered off and washed with DMF (3 x 2 minutes with rotation). A solution of 20% piperdine in DMF was added to the resin and gently agitated by rotation for 2 minutes.

The resin was filtered off and this process was repeated four more times, followed by washing with DMF (5 x 2 minutes with rotation).

DIPEA (98 µL, 0.56 mmols, 11 eq) was added to a solution of Fmoc-Ser(tBu)-OH (101 mg, 0.26 mmols, 5 eq) and HTCU (107 mg, 0.26 mmols, 5 eq) dissolved in the minimum volume of DMF. The resultant solution was then immediately added to the resin. The reaction mixture was gently agitated by rotation for 1 h and the resin was filtered off and washed with DMF (3 x 2 minutes with rotation). A solution of 20% piperdine in DMF was added to the resin and gently agitated by rotation for 2 minutes. The resin was filtered off and this process was repeated four more times, followed by washing with DMF (5 x 2 minutes with rotation).

DIPEA (98 µL, 0.56 mmols, 11 eq) was added to a solution of OPAL linker building block[10] (102 mg, 0.26 mmols, 5 eq) and HTCU (107 mg, 0.26 mmols, 5 eq) dissolved in the minimum volume of DMF. The resultant solution was then immediately added to the resin. The reaction mixture was gently agitated by rotation for 1 h and the resin was filtered off and washed with DMF (5 x 2 minutes with rotation).

DIPEA (1 mL, 5.75 mmols, 55 eq) was added to a solution of mannose azide **4** (46 mg, 0.105 mmols, 1eq), sodium absorbate (31 mg, 0.156 mmols, 1.5 eq) and copper iodide (60 mg, 0.315 mmols, 3 eq) dissolved in DMF (2.5 mL) and the solution was added to resin. The reaction mixture was gently agitated by rotation for 12 h and the resin was filtered off and washed with H_2O (3 x 2 minutes with rotation), iPrOH (3 x 2 minutes with rotation), DMF (3 x 2 minutes with rotation), iPrOH (3 x 2 minutes with rotation), DMF (3 x 2 minutes with rotation).

The resin was washed with DCM (3 x 2 minutes with rotation) and MeOH (3 x 2 minutes with rotation). The resin was dried on a vacuum manifold and further dried on a high vacuum line overnight. A solution of cleavage cocktail (95:2.5:2.5 TFA:H_2O:triisopropylsilane) was added to the resin and gently agitated by rotation for 1 h. The reaction mixture was drained into ice-cold Et_2O and centrifuged at 4000 rpm at 4 °C until pelleted (ca 5-10 minutes). The supernatant was carefully decanted and then subsequently resuspended, centrifuged and supernatant decanted three more times. The precipitated peptide pellet was dissolved in water and lyophilised to obtain a powder of probe **S9**.

Probe **S5** was purified using sephadex LH-20 resin (GE healthcare) in MeOH

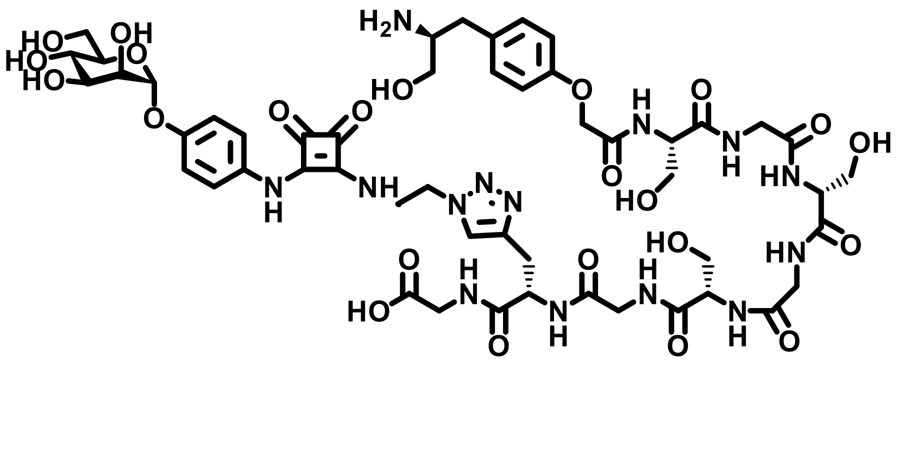

Figure S56. Structure of protected mannose-linked (Gly-Ser)$_3$ OPAL probe **S9**

LC and Mass Spectrum of protected Mannose-(Gly-Ser)$_3$-linked OPAL probe **S9**

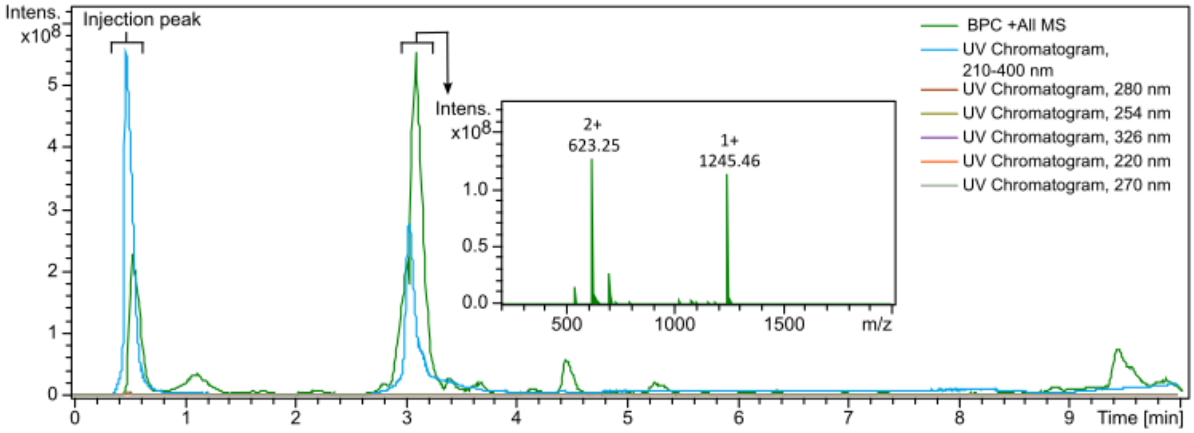

Figure S57. LC trace for protected Mannose-(Gly-Ser)$_3$-linked OPAL probe **S9**

Synthesis of active Mannose-(Gly-Ser)$_3$-linked OPAL probe **6**

To a solution of mannose-based inhibitor linked (Gly-Ser)$_3$ OPAL probe **S9** (500 µL, 16 mM, 10 mg, in 0.1 M PB, 0.1 M NaCl, pH = 7.0) was added $NaIO_4$ (72 µL, 112 mM, in 0.1 M PB, 0.1 M NaCl, pH = 7.0) in 6 × 12 µL increments. The reaction was mixed thoroughly and allowed to sit for 3 minutes on ice in the dark. The solution was then loaded onto a solid phase extraction cartridge (Grace Davison Extract Clean, 8 ml reservoir, Fisher Scientific) equilibrated with water/acetonitrile. After initial washing with water, the product was eluted over a gradient of acetonitrile. The product was then diluted with water, and subsequently lyophilised to give **6** as a pale yellow, fluffy powder (3.5 mg, 36%) which was used crude in OPAL ligation.

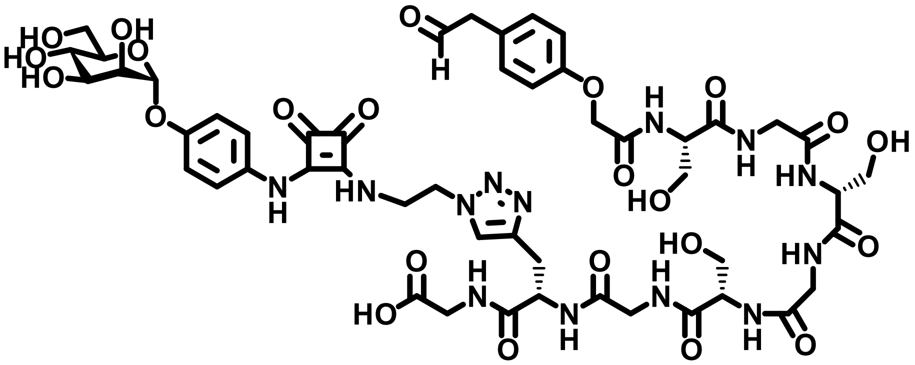

6

Figure S58. Structure of active mannose-linked (Gly-Ser)$_3$ OPAL probe **6**

Synthesis of a Mannose-(Gly-Ser)$_1$-linked colicin E9 conjugate

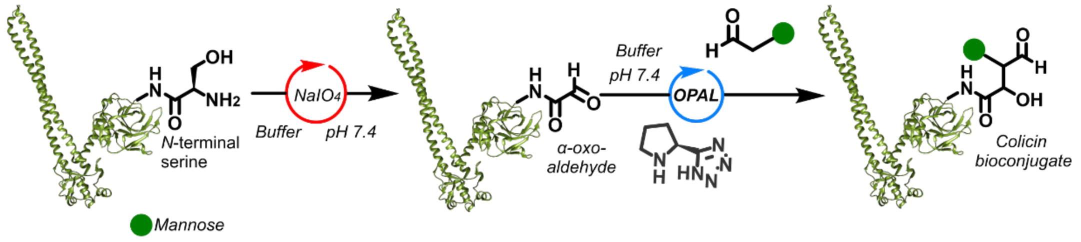

Scheme S4. Shows initial oxidation of the *N*-terminal serine residue of colicin E9 (PDB; 5EW5[1]) with $NaIO_4$ followed by OPAL ligation with a mannose-linked OPAL probe

A solution of colicin E9 (100 µL of 78 µM stock in 25 mM PB pH 7.5) was charged with L-methionine (0.78 µL of 66 mM stock in 0.1 M PB, 0.1 NaCl, pH 7.0) and $NaIO_4$ (0.78 µL of 33 mM stock in 0.1 M PB, 0.1 NaCl, pH 7.0). The solution was mixed by gentle pipette tip swirling and allowed to sit on ice in the dark for 4 minutes. The reaction mixture was immediately purified using a PD SpinTrap G25 desalting column (GE Healthcare Life Sciences), eluting into 100 µL of 25 mM PB pH 7.5. The reaction mixture was charged with (S)-(-)-5-(2-pyrrolidinyl)-H-tetrazole (15.6 µL of 200 mM stock in 25 mM PB pH 7.5) and probe **5** (31.2 µL of 4 mM stock in 25 mM PB pH 7.5). The solution was mixed via pipette tip swirling and incubated for 1 h at 37 °C. The reaction mixture was purified using a PD SpinTrap G25 desalting column (GE Healthcare Life Sciences), eluting into 20 mM K phosphate, 500 mM NaCl pH 7.0 for analysis and further manipulation.

Raw SDS-PAGE gel and Lectin blot

SDS-PAGE gel

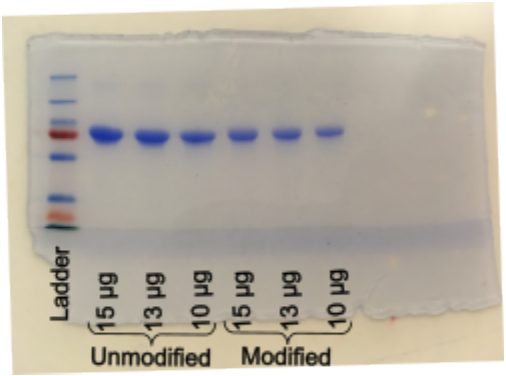

Figure S59. SDS PAGE gel analysis of the Mannose-(Gly-Ser)$_1$-linked colicin E9 conjugate

Lectin Blot

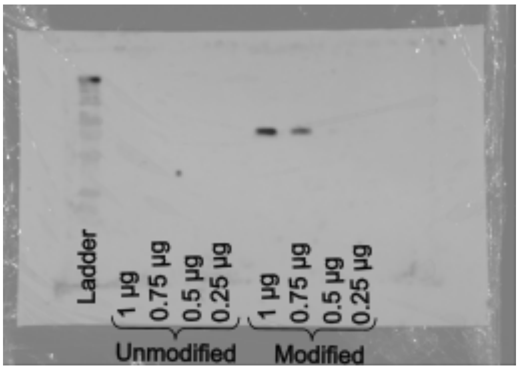

Figure S60. Lectin blot analysis of the Mannose-(Gly-Ser)$_1$-linked colicin E9 conjugate

Synthesis of a Mannose-(Gly-Ser)$_3$-linked colicin E9 conjugate

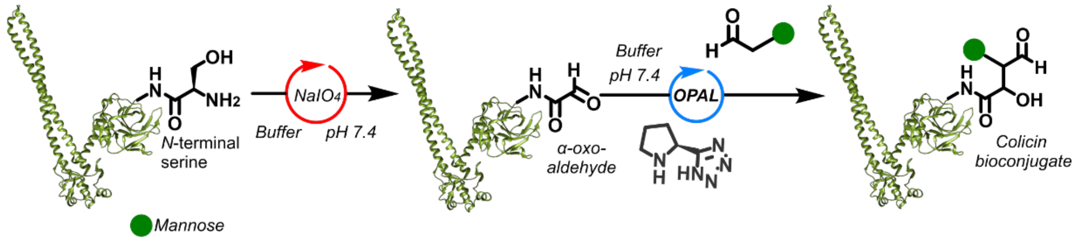

Scheme S5. Shows initial oxidation of the *N*-terminal serine residue of colicin E9 (PDB; 5EW5[1]) with $NaIO_4$ followed by OPAL ligation with a mannose-linked OPAL probe

A solution of colicin E9 (100 µL of 78 µM stock in 25 mM PB pH 7.5) was charged with L-methionine (1 µL of 66 mM stock in 0.1 M PB, 0.1 NaCl, pH 7.0) and $NaIO_4$ (1 µL of 33 mM stock in 0.1 M PB, 0.1 NaCl, pH 7.0). The solution was mixed by gentle pipette tip swirling and allowed to sit on ice in the dark for 4 minutes. The reaction mixture was immediately purified using a PD SpinTrap G25 desalting column (GE Healthcare Life Sciences), eluting into 100 µL of 25 mM PB pH 7.5. The reaction mixture was charged with (S)-(-)-5-(2-pyrrolidinyl)-H-tetrazole (20 µL of 200 mM stock in 25 mM PB pH 7.5) and probe **6** (40 µL of 4 mM stock in 25 mM PB pH 7.5). The solution was mixed via pipette tip swirling and incubated for 1 h at 37 °C. The reaction mixture was purified using a PD SpinTrap G25 desalting column (GE Healthcare Life Sciences), eluting into 20 mM K phosphate, 500 mM NaCl pH 7.0 for analysis and further manipulation.

Raw SDS-PAGE gel and Lectin blot

SDS-PAGE gel

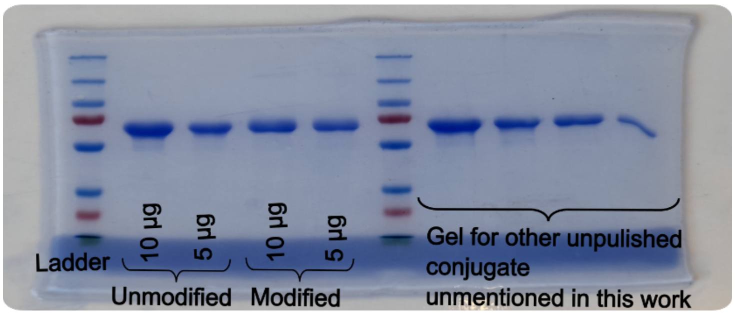

Figure S61. SDS PAGE analysis of Mannose-(Gly-Ser)$_3$-linked colicin E9 conjugate

Lectin Blot

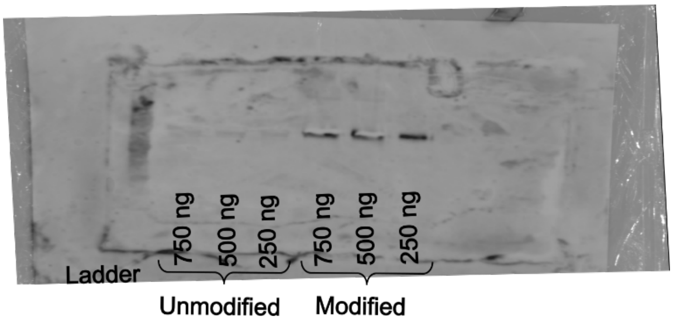

Figure S62. Lectin blot analysis of Mannose-(Gly-Ser)$_3$-linked colicin E9 conjugate

Aggregation experiments

Method

E. coli K12 substr. BW25113, Δ*btuB* and Δ*fimH* cells were incubated in at 37 °C without shaking, in anaerobic conditions for 15 h in supplemented M9 minimal media (plus 30 µg mL^{-1} KAN for Δ*fimH*) using glycerol as a primary carbon source in place of D-glucose without shaking to maximise *fimH* phaseON transitions. Cells were pelleted via centrifugation (8 000 x *g*, 5 min, 4 °C). Pellets were resuspended to an OD_{600nm} of 2.0 in unsupplemented M9 media (plus 30 µg mL^{-1} KAN for Δ*fimH*). ColE9-$(Gly\text{-}Ser)_1$ or ColE9-$(Gly\text{-}Ser)_3$ (for BW25113 WT cells only) was added to samples at final concentrations of 0, 10, 100 and 1000 µM. Samples were incubated for 4 h slowly moving agitation platforms (300 rpm, 25 °C). After incubation 24 µL was removed from each sample and transferred to 500 µL microcentrifuge tubes containing 1 µL 0.5% (w/v) silica bead slurry in unsupplemented M9 medium (pH 7.2). After thorough but gentle mixing 10 µL of this suspension was transferred onto the centre of a 1.0 – 1.2 mm glass slide, covered with a poly-D-lysine coated 18 mm^2 number 1.5 glass coverslip and sealed with a nail varnish bead and left for 10 minutes to set.

z-stack differential interference contrast (DIC) images were collected on a Zeiss 910 LSM upright confocal microscope equipped with 63 x / 1. 46 Na oil immersion objective lens. 10 16-Bit 512 x 512 pixel (0.18 µm px^{-1} resolution) images were collected for each condition during each experimental replicate. 3D depth coded images were produced using ZEN blue software. Aggregation assessments were subsequently carried out using FIJI / ImageJ software.

Briefly, z-stacks slices were combined to form single 2D images. The outlines of all individual and associated cells were then defined using ImageJ 'find edges' tool. The surface area plus two cross sectional measurements (at 90° degrees, defined as 'width' and 'height') were then measured for all individual and associated cell objects. Beeswarm plots summarising widths, heights and areas of BW25113 WT, Δ*fimH* and Δ*btuB* individual and aggregated cells in the presence, and absence of 1 mM ColE9-$(Gly\text{-}Ser)_1$ (and 1 mM ColE9-$(Gly\text{-}Ser)_3$ for BW25113 WT) were used to produce summary beeswarm plots using Origin 2023b software.

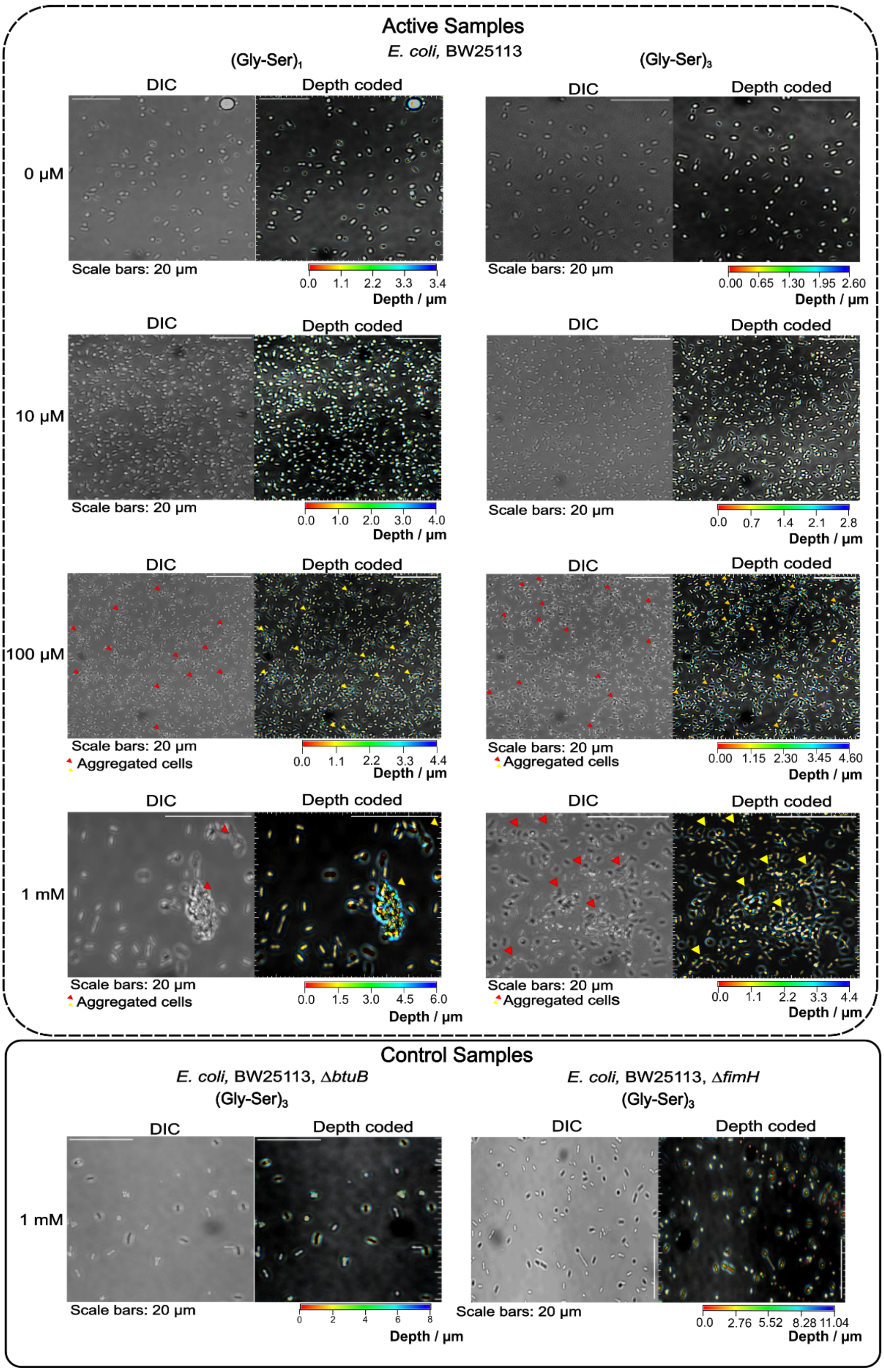

Figure S63. *Top:* DIC and fluorescence confocal microscopy images of samples of *E. coli* BW25113 incubated with increasing concentration of mannose glyco-colicins. *Bottom:* DIC and fluorescence confocal microscopy images of samples of BW25113 ΔbtuB and samples of BW25113 ΔfimH incubated with 1 mM of the mannose linked (Gly-Ser)$_3$ glyco-colicin, demonstrating no aggregation when using control knockout strains.

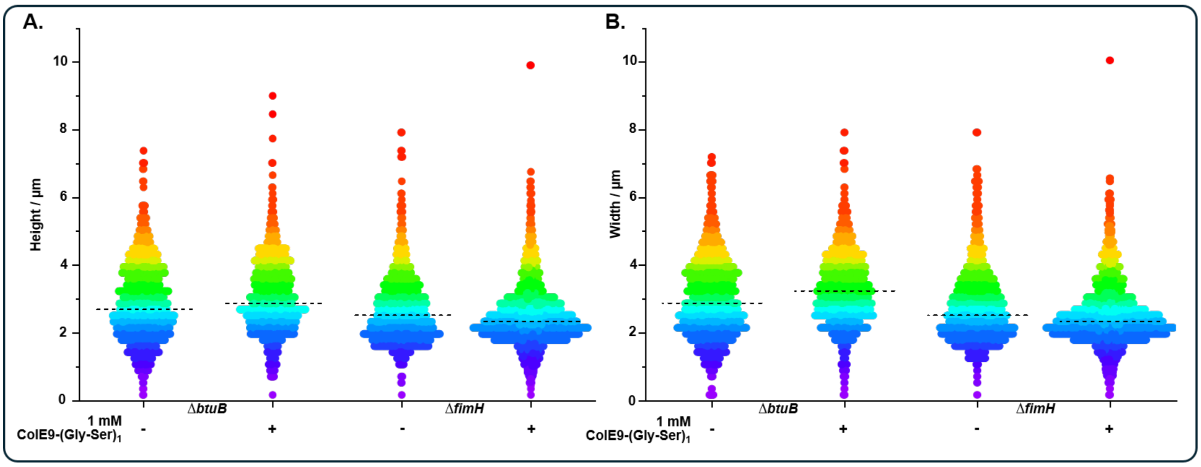

Figure S64. Quantification of individual / aggregated cell object (A.) heights / (B.) widths shows that incubation with 1 mM ColE9-(Gly-Ser)$_1$ does not trigger aggregation in *E. coli* K12 substr. BW25113 Δ*btuB* or Δ*fimH.* Widths and heights were derived from analysis of confocal microscopy images collected over at least 3 experimental replicates (>10 images per replicate) for each condition. *: $p < 0.05$. Dotted lines represent median heights for each condition.

Δ*btuB*

n[0 mM ColE9] = 1290, median height[0 mM ColE9] = 2.7 µm, median width[0 mM ColE9] = 2.8 µm. n[1 mM ColE9(Gly-Ser)$_1$] = 1072, median height[1 mM ColE9(Gly-Ser)$_1$] = 2.9 µm, median width[1 mM ColE9-(Gly-Ser)$_1$ = 3.2 µm.

Δ*fimH*

n[0 mM ColE9] = 1116, median height[0 mM ColE9] = 2.5 µm, median width[0 mM ColE9] = 2.5 µm. n[1 ColE9(Gly-Ser)$_1$] = 1854, median height[1 mM ColE9-(Gly-Ser)$_1$] = 2.3 µm median width[1 mM ColE9(Gly-Ser)$_1$] = 2.3 µm.

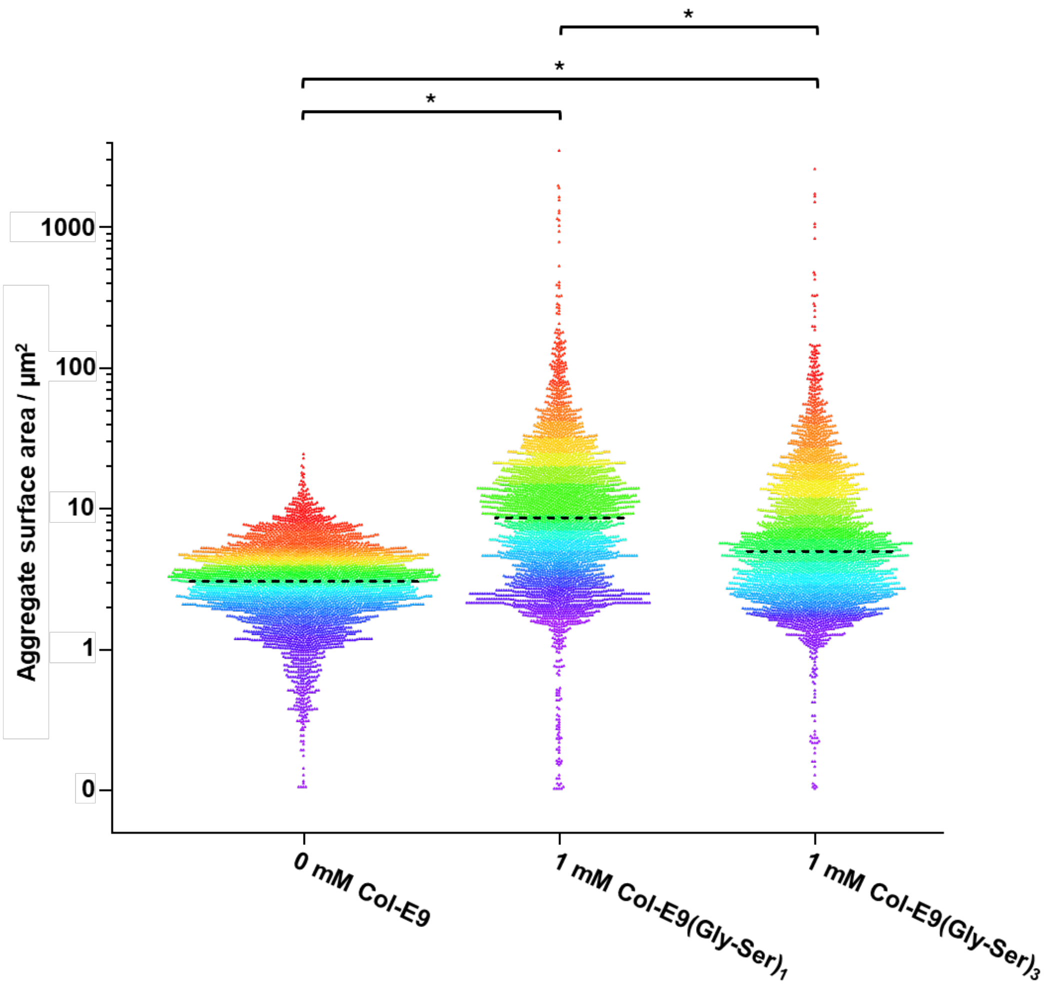

Figure S65. Swarm plots demonstrating incubation with 1 mM Man-Col-E9(Gly-Ser)1 or Man-Col-E9(Gly-Ser)3 induced aggregation in E. coli K12 substr. BW25113 WT cells. Incubation triggered statistically significant positive shifts in the cell / cell aggregate area distributions derived from analysis of fluorescence confocal microscopy images across three experimental replicates for each condition using ZEN Blue / ImageJ software. n[0 mM Col-E9] = 3943, median[0 mM Col-E9] = 3.1 µm^2; n[1 mM Col-E9(Gly-Ser)1 = 3648, median[1 mM Col-E9(Gly-Ser)1] = 8.6 µm^2; n[1 mM Col-E9(Gly-Ser)3] = 3753, median[1 mM Col-E9(Gly-Ser) = 5.0 µm^2. Each point plotted on the swarm plot represents an individual measurement with dotted lines representing median values for the relevant condition.*: $p < 0.05$

Table S1: Mann Whitney U test statistics summaries comparing widths

Condition #1	n(#1)	Median(#1)	Condition #2	n(#2)	Median(#2)	*p*	Z	U
WT-no-treatment	3943	2.302	WT-ColE9(Gly-Ser)$_1$	3648	3.727	0	-40.919	3289084
WT-no-treatment	3943	3.052	WT-ColE9(Gly-Ser)$_3$	3753	3.091	8.159×10^{-256}	-29.459	4528972
WT-ColE9(Gly-Ser)$_1$	3648	3.727	WT-ColE9(Gly-Ser)$_3$	3753	3.091	4.665×10^{-20}	-34.164	4071388

Table S2: Mann Whitney U test statistics summaries comparing heights

Condition #1	n(#1)	Median(#1)	Condition #2	n(#2)	Median(#2)	*p*	Z	U
WT-no-treatment	3943	2.192	WT-ColE9(Gly-Ser)$_1$	3648	3.727	0	-42.897	3100424
WT-no-treatment	3943	2.192	WT-ColE9(Gly-Ser)$_3$	3753	3.157	5.762×10^{-292}	-36.518	3842163
WT-ColE9(Gly-Ser)$_1$	3648	3.727	WT-ColE9(Gly-Ser)$_3$	3753	3.157	3.903×10^{-22}	9.674	7734388

Table S3: Mann Whitney U test statistics summaries comparing areas

Condition #1	n(#1)	Median(#1)	Condition #2	n(#2)	Median(#2)	*p*	Z	U
WT-no-treatment	3943	3.052	WT-ColE9(Gly-Ser)$_1$	3648	8.626	0	-43.462	3045927
WT-no-treatment	3943	3.052	WT-ColE9(Gly-Ser)$_3$	3753	4.997	9.623×10^{-191}	-29.459	4528972
WT-ColE9(Gly-Ser)$_1$	3648	8.626	WT-ColE9(Gly-Ser)$_3$	3753	4.997	9.276×10^{-57}	15.876	8304436

References

(1) Klein, A.; Wojdyla, J. A.; Joshi, A.; Josts, I.; McCaughey, L. C.; Housden, N. G.; Kaminska, R.; Byron, O.; Walker, D.; Kleanthous, C. Structural and biophysical analysis of nuclease protein antibiotics. *Biochem. J.* **2016**, *473*, 2799-2812, Article. DOI: 10.1042/bcj20160544.

(2) Yates, N. D. J.; Akkad, S.; Noble, A.; Keenan, T.; Hatton, N. E.; Signoret, N.; Fascione, M. A. Catalyst-free site-selective cross-aldol bioconjugations. *Green Chem.* **2022**, *24* (20), 8046-8053. DOI: 10.1039/d2gc02292c.

(3) Tufail, A.; Akkad, S.; Hatton, N. E.; Yates, N. D. J.; Spears, R. J.; Keenan, T.; Parkin, A.; Signoret, N.; Fascione, M. A. Cross aldol OPAL bioconjugation outcompetes intramolecular hemiaminal cyclisation of proline adjacent N-terminal α-oxo aldehydes at acidic pH. *RSC Adv.* **2024**, *14* (6), 3723-3729, 10.1039/D3RA08776J. DOI: 10.1039/D3RA08776J.

(4) Su, Y. H.; Xie, J. S.; Wang, Y. G.; Hu, X.; Lin, X. F. Synthesis and antitumor activity of new shikonin glycosides. *Eur. J. Med. Chem.* **2010**, *45* (7), 2713-2718, Article. DOI: 10.1016/j.ejmech.2010.02.002.

(5) Ahmad, M. U.; Ali, S. M.; Ahmad, A.; Sheikh, S.; Chen, P.; Ahmad, I. Carbohydrate mediated drug delivery: Synthesis and characterization of new lipid-conjugates. *Chem. Phys. Lipids* **2015**, *186*, 30-38, Article. DOI: 10.1016/j.chemphyslip.2014.10.003. Zhang, J. B.; Zhang, B.; Zhou, J. F.; Li, J.; Shi, C. J.; Huang, T.; Wang, Z. F.; Tang, J. H2SO4-SiO2: Highly Efficient and Reusable Catalyst for per-O-Acetylation of Carbohydrates Under Solvent-Free Conditions. *J. Carbohydr. Chem.* **2011**, *30* (3), 165-177. DOI: 10.1080/07328303.2011.621042.

(6) Hamed, A.; Osman, R.; Al-Jamal, K. T.; Hoayel, S. M.; Geneidi, A. S. Enhanced antitubercular activity, alveolar deposition and macrophages uptake of mannosylated stable nanoliposomes. *Journal of Drug Delivery Science and Technology* **2019**, *51*, 513-523. DOI: 10.1016/j.jddst.2019.03.032.

(7) Beiroth, F.; Koudelka, T.; Overath, T.; Knight, S. D.; Tholey, A.; Lindhorst, T. K. Diazirine-functionalized mannosides for photoaffinity labeling: trouble with FimH. *Beilstein J. Org.* **2018**, *14*, 1890-1900. DOI: 10.3762/bjoc.14.163.

(8) Lu, M. J.; Lu, Q. B.; Honek, J. F. Squarate-based carbocyclic nucleosides: Syntheses, computational analyses and anticancer/antiviral evaluation. *Bioorg. Med. Chem. Lett.* **2017**, *27* (2), 282-287, Article. DOI: 10.1016/j.bmcl.2016.11.058.

(9) Chauhan, K.; Arun, A.; Singh, S.; Manohar, M.; Chuttani, K.; Konwar, R.; Dwivedi, A.; Soni, R.; Singh, A. K.; Mishra, A. K.; et al. Bivalent Approach for Homodimeric Estradiol Based Ligand: Synthesis and Evaluation for Targeted Theranosis of ER(+) Breast Carcinomas. *Bioconjug. Chem.* **2016**, *27* (4), 961-972. DOI: 10.1021/acs.bioconjchem.6b00024.

(10) Spears, R. J.; Brabham, R. L.; Budhadev, D.; Keenan, T.; McKenna, S.; Walton, J.; Brannigan, J. A.; Brzozowski, A. M.; Wilkinson, A. J.; Plevin, M.; et al. Site-selective C–C modification of proteins at neutral pH using organocatalyst-mediated cross aldol ligations. *Chem. Sci.* **2018**, *9* (25), 5585-5593, 10.1039/C8SC01617H. DOI: 10.1039/C8SC01617H.
